# Under pressure—mechanisms and risk factors for orthodontically induced inflammatory root resorption: a systematic review

**DOI:** 10.1093/ejo/cjad011

**Published:** 2023-06-27

**Authors:** Hassan M Dawood, Annika Kroeger, Vinay Chavda, Iain L C Chapple, Moritz Kebschull

**Affiliations:** Periodontal Research Group, School of Dentistry, Institute of Clinical Sciences, College of Medical & Dental Sciences, The University of Birmingham, Birmingham, UK; Department of Oral Surgery, School of Dentistry, Institute of Clinical Sciences, College of Medical & Dental Sciences, The University of Birmingham, Birmingham, UK; Birmingham Community Healthcare NHS Trust, Birmingham, UK; Birmingham Community Healthcare NHS Trust, Birmingham, UK; Periodontal Research Group, School of Dentistry, Institute of Clinical Sciences, College of Medical & Dental Sciences, The University of Birmingham, Birmingham, UK; Birmingham Community Healthcare NHS Trust, Birmingham, UK; Periodontal Research Group, School of Dentistry, Institute of Clinical Sciences, College of Medical & Dental Sciences, The University of Birmingham, Birmingham, UK; Birmingham Community Healthcare NHS Trust, Birmingham, UK; Division of Periodontics, Section of Oral, Diagnostic and Rehabilitation Sciences, College of Dental Medicine, Columbia University, New York, NY, USA

## Abstract

**Background:**

The application of orthodontic forces causes root resorption of variable severity with potentially severe clinical ramifications.

**Objective:**

To systematically review reports on the pathophysiological mechanisms of orthodontically induced inflammatory root resorption (OIIRR) and the associated risk factors based on *in vitro*, experimental, and *in vivo* studies.

**Search methods:**

We undertook an electronic search of four databases and a separate hand-search.

**Selection criteria:**

Studies reporting on the effect of orthodontic forces with/without the addition of potential risk factors on OIIRR, including (1) gene expression in *in-vitro* studies, the incidence root resorption in (2) animal studies, and (3) human studies.

**Data collection and analysis:**

Potential hits underwent a two-step selection, data extraction, quality assessment, and systematic appraisal performed by duplicate examiners.

**Results:**

One hundred and eighteen articles met the eligibility criteria. Studies varied considerably in methodology, reporting of results, and variable risk of bias judgements.

In summary, the variable evidence identified supports the notion that the application of orthodontic forces leads to (1) characteristic alterations of molecular expression profiles in vitro, (2) an increased rate of OIIRR in animal models, as well as (3) in human studies. Importantly, the additional presence of risk factors such as malocclusion, previous trauma, and medications like corticosteroids increased the severity of OIIRR, whilst other factors decreased its severity, including oral contraceptives, baicalin, and high caffeine.

**Conclusions:**

Based on the systematically reviewed evidence, OIIRR seems to be an inevitable consequence of the application of orthodontic forces—with different risk factors modifying its severity. Our review has identified several molecular mechanisms that can help explain this link between orthodontic forces and OIIRR. Nevertheless, it must be noted that the available eligible literature was in part significantly confounded by bias and was characterized by substantial methodological heterogeneity, suggesting that the results of this systematic review should be interpreted with caution.

**Registration:**

PROSPERO (CRD42021243431).

## Introduction

Orthodontic tooth movement (OTM) is achieved by combining mechanical strain application and subsequent physiological adaptation of the periodontium (1). Crucially, the molecular and cellular responses of the periodontium to both tension and compression are inflammatory in nature, leading to local alterations in the surrounding environment that affect the blood supply, the release of various neurotransmitters, growth factors, and cytokines (2,3).

Periodontal ligament cells (PDLs) are necessary mechanoreceptors and primary shock absorbers that respond to constant and intermittent loading (4). In addition, PDL cells are essential for periodontal remodelling, bone resorption, and tooth movement (4,5). The activation of PDL cells leads to characteristic expression profiles, ultimately driving altered cellular responses (6,7).

Orthodontically induced inflammatory root resorption (OIIRR) is a clinically challenging, well-documented side effect of OTM (8). It is understood as a multifactorial pathological process that results in the loss of permanent root surface structure with varying severity (1,9). In advanced cases, OIIRR can have a detrimental impact on the long-term prognosis of affected teeth leading to tooth loss as a worst-case scenario—impacting patients’ quality of life (10,11).

Therefore, it is of critical importance for clinicians to understand why OIIRR occurs and which patients are, based on these mechanisms, most at-risk of severe OIIRR. A plethora of research focuses either on the underlying mechanisms of OIIRR or the risk factors that can predispose to severe resorption, but typically, the biological mechanisms and the clinical factors are seldom combined (9,12).

Generally, factors can be divided into patient-related risk factors or treatment-related factors. The patient-associated factors can be the race, the age that involves both chronological and dental age (13,14,15), individual susceptibility and sex (21,22,16,18,17,20,14,19,23,15) genetic (38,39,24,25,36,37,29,9,34,32,35,31,33,26,28,27,30), asthma (40,41), systematic and social factors (42,43), diabetes (44), nutrition (45), medications (46,47), and others.

The local factors are type and severity of the malocclusion (49,48), habits including bruxism, tongue thrust finger sucking and nail-biting (50,51), previous trauma (52,19), endodontically treated teeth (12,53), morphological characteristics and development of the root (11,19), and others.

The treatment-related or intervention-related are, for example, duration of treatment (22), direction and magnitude of force (54,19), intermittent versus continuous forces (20), the distance of movement (55), type of appliance (56) a history of tooth extraction (57), treatment mechanics (58), intentionally injected materials or medication to alter root resorption (60,59) and others. These factors can interact with each other and result in the development of the phenotype.

Therefore, we sought to systematically review reports on the pathophysiological mechanisms of OIIRR and the linked systemic and local clinical risk factors for OIIRR based on *in-vitro*, *in-vivo* animal, and human studies.

## Materials and methods

### Study protocol and registration

Before undertaking this review, we created a specific protocol in line with the PRISMA guidelines and checklist (61,62). The study was registered with the International Prospective Register of Systematic Reviews (PROSPERO reference CRD42021243431).

### Research question

(i) The research questions were developed according to the PI/ECOT format; that is, the Population Intervention/Exposure, Control, Outcome, and Time (63).
(ii) **Molecular responses**

**PICOT 1A:** Are molecular expression profiles [O] different in human periodontal ligaments cell culture models of OTM [P] with different active mechanical forces [I] compared to cell culture models without force application?
**PE/ICOTS 1B:** Are molecular expression profiles [O] different in human periodontal ligaments cell culture models of OTM with active mechanical forces [P] with possible risk factors [E] compared to cell culture models without risk factors?

(i) **Pre-clinical responses**

**PICOT 2A:** Is the incidence of root resorption [O] different in teeth exposed to different active mechanical orthodontic forces [I] compared to teeth not exposed to mechanical force [C] after a minimum follow-up of 2 weeks [T] investigated in animal models [P]?
**PE/ICOT 2B:** Is the incidence of root resorption [O] on teeth exposed to orthodontic forces [P] higher in animals/situations with potential systemic or local risk factors [E] compared to animals/situations without such risk factors [C] after a minimum follow-up of 2 weeks [T]?

(ii) **Clinical responses**
(iii) **PICOT 3A:** Is the incidence of root resorption [O] in systemically healthy patients [P] different in teeth exposed to orthodontic treatment with different active mechanical forces [I] compared to teeth not exposed to mechanical force [C] after a minimum follow-up of 4 weeks [T]?

**PE/ICOT 3B:** Is the incidence of root resorption [O] in a population of patients undergoing orthodontic treatment [P] higher in patients/situations with potential systemic or local risk factors [E] compared to patients/conditions without such risk factors [C] after a minimum follow-up of 4 weeks [T]?

### Inclusion criteria

#### General

- Comparative studies (e.g. randomized-controlled trials (RCT), controlled clinical trials (CCT), case-control studies, comparative cohort studies)
- Reporting on high-throughput sequencing methodology
- Written in English language

#### PE/ICOT 1s

- In vitro human PDL cell culture models

#### PE/ICOT 2s and 3s

- Animal studies for PE/ICOT 2
- Group size ≥ 10 participants at the time of measurement of the outcome
- Minimum follows up of two weeks for human studies and 4 weeks for animal studies

### Exclusion criteria

#### General

- Non-English literature
- Non-comparative studies (e.g. case series, case reports, cross-sectional non-comparative studies)
- Systematic reviews
- Not reporting on high-throughput sequencing methodology

#### Specific to PE/ICOT 1

- Non-human, non-PDL cells, non-force culture in vitro models

#### Specific to PE/ICOT 2/3

- Group size less than ten at the time of measurement.
- Internal root resorption.
- Root damage due to placement of a miniscrew or other surgical procedure.
- Root resorption due to ectopic or unerupted teeth.
- Abnormal tooth morphology.
- Deciduous teeth.
- Clefts of lip and palate and other craniofacial disorders
- Auto-transplantation.
- Abnormal tooth morphology.
- Deciduous teeth.
- Clefts of lip and palate and other craniofacial disorders (in human studies).
- Auto-transplantation.

### Search sources and strategy

The following electronic databases were searched electronically for peer-reviewed publications (The searches were performed on 23 March 2021, and an updated search was conducted on 31 May 2022):

- MEDLINE (Medical Literature Analysis and Retrieval System Online via PubMed).
- CENTRAL (The Cochrane Central Register of Controlled Trials).
- Embase.
- SCOPUS.

A manual search for eligible publications:

- The last 20 years of the following journals: European Journal of Orthodontics (volumes 3–43), Journal of Dental Research (volumes 59–100), The Angle Orthodontist, and American Journal of Orthodontics and Dentofacial Orthopedics (77–159).
- Reference lists of included publications.
- Controlled Trials in ClinicalTrials.gov.
- Experts in the topic.

For search terms, please refer to Supplementary Table 1.

### Study selection

First, titles and abstracts were screened by two independent reviewers (HD, VC). Abstracts that—according to the two reviewers—met all inclusion and exclusion criteria were considered. An initial calibration exercise was undertaken. The inter-rater reliability was calculated (percentage of agreement). Any disagreement was referred to a third reviewer (AK) and discussed to reach an agreement. For the next step—the full-text search was conducted independently by HD and VC. Any difference of opinion was consulted with a third reviewer (AK) for the final decision.

### Data extraction

Two reviewers (HD, VC) independently performed duplicate data extraction utilizing a pre-established and trialed spreadsheet as recommended by the Cochrane Handbook (63). In addition, data figure software (Plot digitiser) extracted the numerical data from the figures when it was not available in the text (64).

### Outcomes

#### PE/ICO1a&b

The main outcome is

- The genomic changes of PDL cells after mechanical force application.
- The additional outcomes are: relationship between the measured molecular and genetic expression and root resorption mechanism.

#### PE/ICO2a&b

The main outcome was:

- Incidence of OIIRR

The additional outcomes were:

- Gingival index
- Periodontal disease incidence
- Molecular biomarkers
- Histological findings

#### PICO3a&b

The main outcome was:

- Incidence of OIIRR.

The additional outcomes were:

- Gingival index
- Periodontal disease incidence
- Mobility
- Tooth loss
- Molecular biomarkers
- Histological findings
- Patient-related outcomes: pain and quality of life

### Quality assessment

Two reviewers (HD, VC) independently assessed the risk of bias using multiple tools. Any disagreements were resolved by a third reviewer (AK).

- The Cochrane Collaboration Risk of Bias (RoB 2.0 tool) was used for randomized trials (65).
- Risk Of Bias In Nonrandomised Studies of Interventions (ROBINS-I tool) (66).
- Newcastle-Ottawa-Scale was used for observational studies (67).
- The Systematic Review Centre for Laboratory Animal Experimentation (SYRCLE) was used for the animal studies (68).
- A modified Animal Research: Reporting of In Vivo Experiments (ARRIVE) (69) was used for cellular studies, as it has been previously used successfully in other systematic in vitro reviews (70,71).

### Overall Quality of Evidence Assessment (GRADE)

The quality of evidence was evaluated according to the Grading of Recommendations, Assessment, Development and Evaluations (GRADE) approach (162); Higgins *et al*. (161).

According to the GRADE methodology (161), the certainty level was downgraded by one level for serious concerns and two for very serious concerns in five domains: RoB, inconsistency, indirectness, imprecision, or publication bias. Vice versa, they were upgraded by one or two levels if large magnitudes of effects were recorded, there was evidence of a dose–response gradient, or plausible confounders would have reduced the demonstrated outcomes or all residual confounding and biases would have increased the effect if no effect has been observed.

## Results

### Study selection

**Figure 1.**
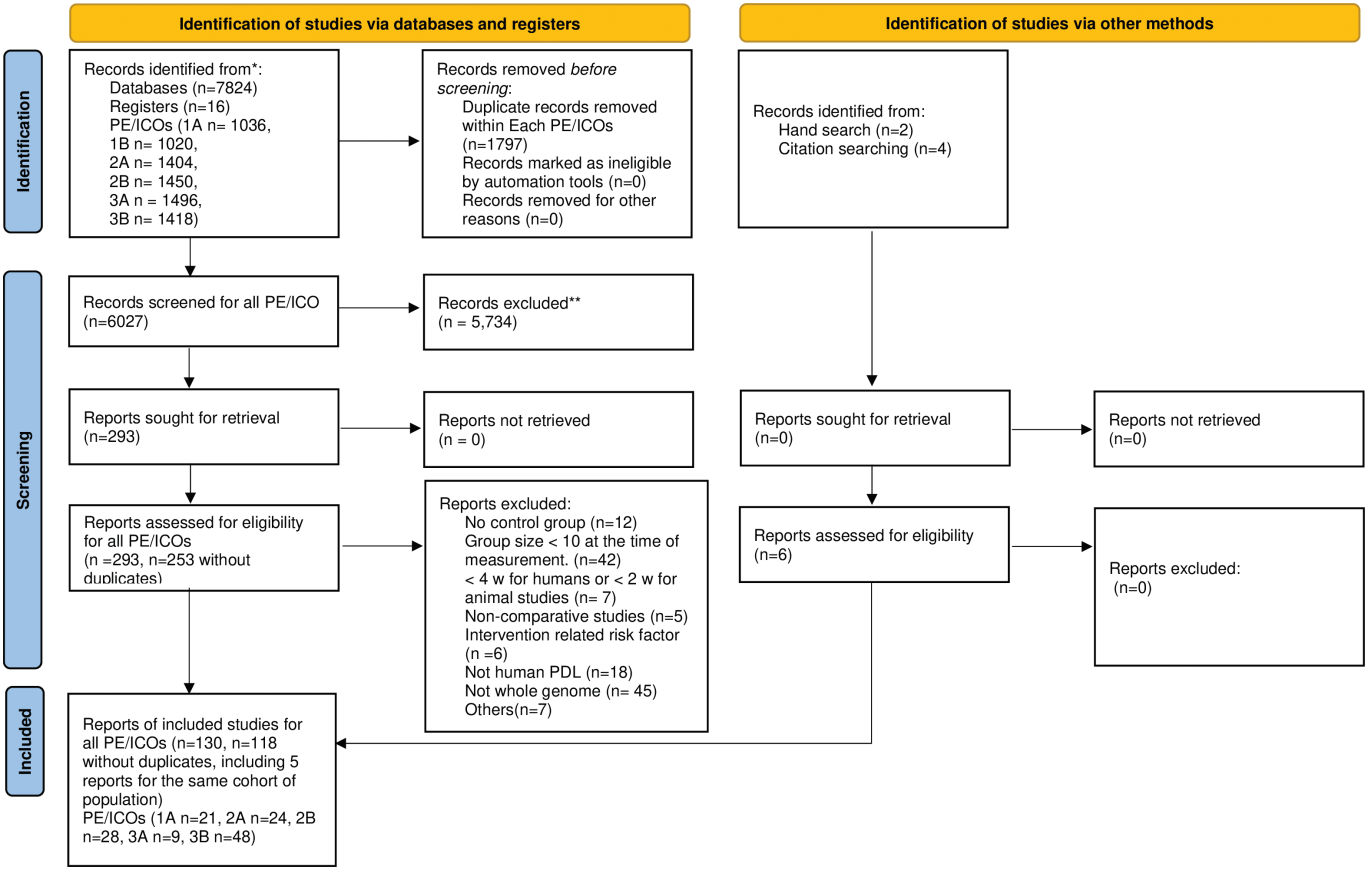
PRISMA flowchart.

A total of 7824 studies were identified through electronic searches. However, 6027 hits remained after the removal of duplicates. After title and abstract screening, a total of 253 full texts were assessed for eligibility using full-text assessment. Overall, 113 individual studies, some applicable for more than one PICO, were included (Figure 1 PRISMA flowchart). A list of excluded articles in the full-text stage, including reasons for exclusion, can be found in Supplementary Table 2.

### Inter-rater agreement

The inter-rater raw agreement rate during the screening stage of the titles and the abstracts was 95.8%.

The Kappa correlation coefficient value was (*k* = 0.817) for the risk of bias analyses (72), which is considered very good. The inter-rater agreement was 89.1%.

### Overall quality of evidence (GRADE)

The quality of evidence was evaluated using the GRADE approach. As no meta-analysis was undertaken, no test for publication bias was performed. Forest and funnel plots were not constructed due to high heterogeneity amongst study designs, methods used, and selected outcome measures.

### General details and study design of the included studies

#### PICO 1A

Are molecular expression profiles [O] different in human periodontal ligaments cell culture models of OTM [P] with different active mechanical forces [I] compared to cell culture models without force application?

### General characteristics

Twenty-one articles were selected (73,74,75,76,3,77,78,79,152,90,80,81,6,82,83,84,7,85,86,87,88). All were published between 2007 and 2021, using total RNA extraction and bulk sequencing. The type of force was compressive (CF) or stretching tensile, constant or cyclical. The loading time ranged from 0.5 to 72 h.

### Risk of bias

Thirteen *in vitro* studies were well-formulated and received high scores; all studies scored between 14 and 19 out of 20 points (Supplementary Table 3).

### Outcomes of included studies

The main outcome was reported as increasing or decreasing in fold change of gene expression compared to the control group. The goal measurement of these studies was total RNA extract, mRNAs, miRNAs, or lncRNAs expressions and their interaction networks and pathway activation.

The summary of the included studies is listed in Supplementary Table 4. Detailed studies’ descriptions and outcomes are presented in Supplementary Table 5. The most upregulated and downregulated genes are shown in Supplementary Tables 6 and 7, respectively. No secondary outcomes were found.

#### PE/ICO 1B

Are molecular expression profiles [O] different in human periodontal ligaments cell culture models of OTM with active mechanical forces [P] with possible risk factors [E] compared to cell culture models without risk factors?

No studies were found related to this PE/ICO.

#### PICO 2A

Is the incidence of root resorption [O] different in teeth exposed to different active mechanical orthodontic forces [I] compared to teeth not exposed to mechanical force [C] after a minimum follow-up of 2 weeks [T] investigated in animal models [P]?

### General characteristics

In this PICO, 24 articles were selected for data extraction, 1 on mice (91) and 23 on three different types of rats (Wister, Sprague-Dawley, and Fischer-344) (92,93,94,95,96,97,98,99,100,10,101,2,102,103,104,105,106,107,108,109,110,111,112). They were published between 1997 and 2021. All used a mesial tipping force on molars with close coil springs, except two utilized an expansion force (98,103). Follow-up times ranged between 14 and 35 days.

### Risk of bias

The (SYRCLE) tool was used for PICO 2A and PI/ECO 2B. However, most authors rarely mentioned if allocation was randomized or if the animals were kept in random houses or commented on the blindness of the assessors, resulting in unclear certainty in many domains (Supplementary Table 8).

### Outcomes of included studies

All results showed that force-tested groups have a significantly higher risk of OIIRR than the no-force control groups (92,93,94,95,91,96,97,98,99,100,10,2,102,103,104,105,106,107,108,109,110,111,112). Only one study reported no significant difference in OIIRR between the intermittent force and control Groups (103). Eight studies had data for the secondary outcomes; histological and molecular findings (91,100,10,101,2,102,104,106). The summaries of the included studies are listed in Supplementary Table 9. Detailed studies’ descriptions and outcomes are presented in Supplementary Table 10.

#### PE/ICO 2B

Is the incidence of root resorption [O] on teeth exposed to orthodontic forces [P] higher in animals/situations with potential systemic or local risk factors [E] compared to animals/situations without such risk factors [C] after a minimum follow-up of 2 weeks [T]?

### General characteristics

Twenty-eight articles were included for data extraction, 1 on mice (91) and 27 on three different types of rats (Wister, Sprague-Dawley, and Fischer-344) (92,93,113,114,115,95,116,117,97,118,10,119,2,120,102,121,122,123,124,105,125,106,126,128,108,109,127). All articles were published between 2003 and 2021.

### Risk of bias

Please refer to section PICO 2A RoB, as the same tool was used for PE/ICO 2B.

### Outcomes of included studies

Higher risks of OIIRR were found in groups with high bone turnover, acute corticosteroid intake than chronic corticosteroid intake or control groups, 4-hexylresorcinol medication intake, hypofunctional periodontium, nicotine, and strontium ranelate (118,119,126,128,108,109).

Significantly less OIIRR was reported in animals with a chronic combined oral contraceptive, baicalin intake, sites with previous bone ceramic or bio osseous graft, systemic sodium fluoride, immature roots, osteopontin (OPN) deficiency, angiogenic cytokines, high caffeine, ovariectomy, and orchiectomy rats (91,97,2,120,102,123,124,105,125,127).

Studies investigating the effects of a diet with high casein phosphopeptides (CPP), high zinc in the diet, carboxymethyl cellulose and atorvastatin medication, lacunae size in amitriptyline medication, cetirizine intake, zoledronic acid, etoricoxib medicine, fluoxetine, budesonide, ovalbumin and in systemic fluoride did not report significant differences between test and control groups (92,93,113,114,95,116,117,10,121,122,106).

In terms of secondary outcomes, 19 studies reported histological and/or biological findings (93,114,95,91,118,10,119,2,120,102,121,122,105,125,106,126,127). The summaries of the included studies are listed in Supplementary Table 11. Detailed studies’ descriptions and outcomes are presented in Supplementary Table 12.

High quality of evidence assessed via GRADE Methodology indicates that orthodontic forces in PICO 2A and/or intervention or exposure in PI/ECO 2B are associated with the incidence or increased severity of OIIRR in animal studies (Supplementary Table 13).

#### PICO 3A

Is the incidence of root resorption [O] in systemically healthy patients [P] different in teeth exposed to orthodontic treatment with different active mechanical forces [I] compared to teeth not exposed to mechanical force [C] after a minimum follow-up of 4 weeks [T]?

### General characteristics

Nine articles were selected for data extraction (13,129,134,135,130,36,131,132,133). All articles were published between 1986 and 2021. The follow-up varied from 28 days to 6 months.

### Risk of bias

Six studies were RCTs (129,130,131,133), two with low risk (131,133), and four were of some concern. One CCT was assessed to have a moderate RoB (135). The last two were case-control studies with a low RoB (13,36) (Supplementary Table 14).

### Outcomes of included studies

The experimental group in all studies with force application showed an increased incidence of resorption and/or severity of OIIRR. The reported OIIRR was the resorption crater volume (129,133), the ratio of reduction in root length (135), volume of cervical RR (130), and the percentage of RR severity between the groups (131), as compared to the control group with no-force application.

Two studies reported relevant secondary outcomes (131,132): no significant association was found between OIIRR and patient hygiene level or periodontal parameters (PI, GI, BOP, PPD) The summary of the included studies is listed in Supplementary Table 15. Detailed studies’ descriptions and outcomes are shown in Supplementary Table 16.

#### PE/ICO 3B

Is the incidence of root resorption [O] in a population of patients undergoing orthodontic treatment [P] higher in patients/situations with potential systemic or local risk factors [E] compared to patients/conditions without such risk factors [C] after a minimum follow-up of 4 weeks [T]?

### General characteristics

Forty-eight articles were selected for data extraction (38,39,136,137,13,138,24,25,139,36,37,29,18,140,9,34,32,35,31,33,45,12,141,142,11,26,53,143,144,150,14,40,41,145,49,19,23,146,50,28,27,15,147,30,148,149,48,47). All articles were published between 1982 and 2022. Treatment times varied from 2 weeks to 47 months.

### Risk of bias

Two studies were RCTs, one with low RoB (45) and the other with some concerns (53). Three were CCT studies, two were medium (12), and the other was critical (47). The remaining 43 were either cohort or case-control studies; 28 were assessed with low, 9 with medium, and 6 with a high RoB. (38,39,136,137,138,24,25,139,37,29,18,140,9,32,34,35,31,33,78,142,11,26,144,14,40,41,145,49,19,23,146,50,28,27,15,147,30,148,48) (Supplementary Table 14).

Analysis for reporting bias across the studies revealed that nine reports were on the same cohort of the population (137,26,28,27,34,35,33,24,24). This was confirmed by emails from some authors of these publications (Linhartova p. and Iglesias-Linares A.). Therefore, each was evaluated as a different report of the same study, including the measurable outcomes.

### Outcomes of included studies

The main finding of these studies was an immediate significant increase in the risk of OIIRR with:

- Asthma (40),
- Atopy (allergies) (19,23,146)
- Cortical thickness (15),
- Incisor proclination (24)
- Increase in age (25,36,140,41,23,15),
- Initial degree of RR (14,15),
- Initial root length (25,145,23,15),
- Initial size of the root (36),
- Males (23,28,27,148),
- Occlusion; overbite (49,23),
- Overjet (23) and Class II (48),
- Presence of periapical pathology (142),
- Previous trauma (13,23).
- RFT with increased age (142),
- Root shape and width (145),
- Small or peg shape tooth (141,23)

Concerning genetic studies, a significantly increased risk of OIIRR was found in:

- Specific genotypes as in purinergic-receptor-P2X, ligand-gated ion channel 7 SNP rs208294 (30).
- IL1RN*12, *22 genotypes, the short two alleles (IL1RN*2, with two repeats compared to three to six repeats in long alleles) in the subgroup of girls and VNTR variants in girls (26).
- IL-1A variation with genotype 2-2 (41).
- GG genotype of rs1718119 from P2RX7 gene (27).
- Specific haplotype of P2RX7 (rs208294) and IL1RN (rs419598) with SNPs (24).
- RANK polymorphisms, in rs12455775, rs3102724, rs2875845, rs1032128, and rs3102728, osteoprotegerin polymorphisms, (25).
- The microsatellite marker D18S64 (tightly linked to TNFRSF11A) (38).
- Association of IL-1B polymorphism with Max CI EARR, IL-1B (1,1), which have a 5.6 fold increased risk of EARR greater than 2 mm, compared with subjects who were not homozygous for the IL-1B allele 1 (39).
- Homozygous [2/2(TT)] for the IL1B gene in root-filled teeth (35).
- IL1B gene in the comparative analysis of homozygous subjects (2/2[TT]) and (1/1[CC]), which led to an increased risk of experiencing post orthodontic EARR in root-filled teeth (32).
- In root-filled teeth in subjects with homozygous [1/1(TT)] for the IL1RN gene (33).
- IL-1B[1/1(CC)] and IL-1RN [1/1[(TT)] gene (32).
- The rs1143634 in allelic model, the A allele of IL-1β SNP (136), and X chromosome located genes in men: STAG 2 gene, stromal antigen 2 genes, rs151184635, and RP1-30E17.2 gene, rs55839915 (145).

Some studies also identified possible protective factors in reducing OIIRR severity, such as

- Root-filled teeth compared to vital, immature teeth, and systemic fluoridation (45,12,142,11,150,148).
- Homozygosity/hemizygosity for variant C from the IRAK1 gene and the heterozygous and homozygous osteopontin gene allele at position 89261521 (33,27).

In contrast, some studies found no significant differences between experimental and control groups regarding age, gender, tooth length, root shape, tooth type, asthma, root-filled teeth, habits, nail-biting, gingivitis, medications, and overbite (138,139,29,18,142,53,143,14,41,50,147). only two studies mentioned data related to the secondary outcomes (50,47). the summaries of the included studies are listed in Supplementary Table 17 with more details are shown in Supplementary Table 18.

High quality of evidence according to applied GRADE methodology indicates that orthodontic forces in PICO 3A and/or intervention or exposure in PI/ECO 3B are associated with the incidence or increased severity of OIIRR in human studies (Supplementary Table 19).

## Discussion

### Summary of evidence

This work aimed to systematically review the existing literature on the effect of mechanical force (± patient-related risk factors) on the incidence of root resorption from *in vivo* studies. Further, we aimed to elucidate the underlying pathophysiological process of OIIRR by assessing cellular ‘-omics’ changes *in vitro* studies.

The systematic review of current literature demonstrated that OIIRR occurs under mechanical stress in humans and animals. Further, it can be concluded that different forms of pressure application are associated with differing severities of OIIRR.

#### PICO1

All *in vitro* studies reported a stimulating effect of mechanical force on human PDL cells in an artificial environment. However, these investigations primarily focused on the interaction at the cellular level whilst not considering the interaction with the environment. These effects are expressed either as increased or decreased expression of genes and pathways, which differed between studies due to the magnitude of applied force, application method, extraction devices, and sequencing methodology. The results of the expression profiles were reposted using fold change compared to controls. Only the significant expression data were collected.

##### mRNAs expression profiles

It was found that the CF elevated interleukin 6(IL-6), interleukin-1b, ADAMTS1, cyclooxygenase (COX)-2, and osteocalcin (76,78,6). Both constant and intermittent CF increases SOST, TGFB1, and HEY1 mRNA expression. In contrast, the HES1 mRNA and TGF-β signalling, which affect osteogenic differentiation, are increased in intermittent CF but not in constant CF. Other experiments found a reduction in alkaline phosphatase (ALP), -a marker for new bone formation under different forces. Reduction in the Expression of FOXM1 under CF enhanced the osteoclastic differentiation and the receptor activator nuclear factor-kB ligand (RANKL)/OPG ratio. An increase in RANKL was also found after COX-2 activation and prostaglandin E2 (PGE2) induction. OPG mRNA was found to be upregulated by cyclic tension, which has a negative effect on osteoclastogenesis and transferring this gene to the periodontal area results in the reduction of OTM (75,76,78,79,80,6,82,83,86,88). These findings might shed light on the role of PDL cells in the pathophysiological mechanisms of OIIRR.

##### snRNAs expression profiles

Wei *et al*. identified 53 differentially expressed miRNAs from PDL cells under pressure compared to PDL cells without mechanical force; 26 were upregulated; and 27 were downregulated (85). Evidence suggested that miRNAs expression profiles were related to osteogenesis activation or inhibition by their effect on transcriptomes and osteogenic related proteins production (among them miRNA-221-3p, miRNA-138-5p, miRNA-132-3p, miRNA-218-5p, miRNA-133a-3p, miRNA-145-3p, miRNA-143-5p, miRNA-486-3p, miRNA-29, miRNA-101, and miRNA-21-3p as well as osteogenic related signalling pathways) (73,74,7,85,87). Tension load affects miRNA-195-5p, miRNA-424-5p, miRNA-1297, miRNA-3607-5p, miRNA-145-5p, miRNA-4328, miRNA-29, and miRNA-224-5p expression, which downregulate several genes expression including FGF2, PAG1, ERBB3, HSPH1, IKBKB, WDR33, RIF1, and CREB1, and, in turn, downregulate bone formation (73,74,7,87). The discovery of the differential miRNA expression under mechanical loading and the transcription factor can provide clues about osteogenic differentiation and fill part of the jigsaw puzzle for the mechanisms involved in bone remodelling, root resorption, and OTM.

##### lncRNAs expression profile

Crucial evidence was discovered regarding the lncRNAs’ involvement in cell differentiation and development and the osteogenesis pathways and force effect on their expression levels (3,89,90,151). The lncRNA has been reported to act as sponges for microRNAs (miRNAs) and may interact with miRNAs to control their downstream destinations (153). Under pressure, the lncRNAs promote osteogenesis through the focal adhesion kinase pathway (FAK), the mitogen-activated protein kinase pathway, the TGF-β1/Smad3/HDAC signalling pathway and the downregulation of ANCR expression. It can also regulate the mRNAs’ expression by enhancing the binding ability of miRNAs. Wang *et al*. novelty reported on the network of the lncRNAs-miRNAs-mRNAs relationship of the PDL stem cells under tension (3,7). These studies provide insight into the role of the lncRNA and its association with the mRNAs and miRNAs expression in osteogenic differentiation and bone and root resorption.

#### PICO 2 and 3

##### Evidence in both human and animal studies

Many risk factors were identified in the current review and can be divided into systemic and local factors. As stated earlier, root resorption has an inflammatory origin; increasing the impact of inflammation leads to more severe OIIRR. This may explain the increase in OIIRR in many studies with low to moderate risks of bias in human studies, including roots with periapical pathology (142), hypofunctional teeth and periodontium in human (49) and animal studies (126), and previous trauma of teeth and the surrounding periodontium in human reports (13,23).

The overall certainty of the evidence using the GRADE tool for each PI/ECO was determined to be highly variable, ranging from low to very low, with few studies being graded at moderate and high certainty (Supplementary Tables 13 and 19).

Immature roots have less OIIRR volume than mature teeth, which may be due to the decreased expression of Jagged1, Notch2, IL-6, and RANKL signalling in both low-risk human studies but higher-risk animal one (2,11).

Unspecific allergic reactions were amongst the prominent systemic risk factors of OIIRR, with human studies of moderate evidence (19,23,146), insufficient evidences (50), and unclear evidences of an animal study (92).

Furthermore, systematic fluoride is a protective factor against RR in two high-quality RCT and CCT human studies (45,150) and two animal studies (97,120). Only one animal report stated that this effect is insignificant (117).

##### PI/ECO 2 animal studies

There is moderate evidence for a reduction in the volume of OIIRR of teeth adjacent to an extraction site of rats grafted with bone-ceramics, which might be explained by a better osteoinductive potential than naturally recovered sites or natural bovine cancellous bone particles graft (123).

There is moderate evidence for a reduction in the volume of OIIRR of teeth adjacent to an extraction site of rats grafted with bone-ceramics, which might be explained by a better osteoinductive potential than naturally recovered sites or natural bovine cancellous bone particles graft (123).

Medication intakes pose an additional risk factor in the development of OIIRR in animal studies, including ones that increase the severity of OIIRR with strong evidence like strontium ranelate intake (119) or with moderate to low evidence like Corticosteroids (109) and 4-hexylresorcinol medication (118).

Oral contraceptive intake, on the other hand, decreases the severity of OIIRR due to the effect on the hormonal level that affects periodontal tissue and cementum metabolism (102,127) suggests that assessment of individual bone metabolism pre-orthodontic treatment may give insight into the rate and severity of OIIRR (108).

Similar associations were shown in rats with decreased steroid-sex hormone plasma levels due to bone turnover reduction (124).

Another study with moderate evidence found that increasing caffeine intake might also reduce OIIRR occurrence, which might be due to the inhibition of the formation of osteoclasts that leads to decreased bone turnover and resorption (125).

##### PI/ECO 3 human studies

Occlusal abnormalities such as an increased overjet and Angle’s class II malocclusion are of substantial evidences to be associated with more severe OIIRR; this may be caused by increased treatment times or the amount of applied force (23,48).

In addition, increased incisal inclination and the presence of an obstacle obstructing the path of root movement (e.g. impacted teeth, increase in cortical thickness) may lead to more stress on the root and subsequently to an increase in odontoclastic effects, with high-quality human studies (138,54,24,15).

Biological sex seems to have a controversial effect on the OIIRR among studies; Four studies with strong evidence reported increased OIIRR in male compared to female participants (32,23,28,27,148). In comparison, others with medium evidence do not find an effect of a sex difference (29,142,14).

Higher age was associated with increased OIIRR in human observational studies, 4 of which are of high quality (25,36,140,41,23,15), whilst three reported no association with low to medium risks (18,142,14). Age may increase OIIRR due to the increased risk of iatrogenic damage by orthodontic forces (154). Consequently, special consideration and measures should be contemplated to manage root resorption risk in adults, such as increasing the gap between visits, less force, and maintaining oral hygiene during treatment (155,156). High-quality RCT studies are needed to solve the controversy.

Data from low risks observational studies conclude that the association between asthma and OIIRR is inconclusive; one shows a link to increase OIIRR risk (40), and the other shows no link (41).

The initial size of the root, initial root length, shape, and width are significant factors in some observational variable qualities studies (25,36,145,23,15). In contrast, other low-evidence retrospective studies reported no effect (50,147). These conflicting outcomes may be linked to the different methodologies, treatment time, and measurement techniques.

Genetic considerations may be an important perspective for the incidence and severity of OIIRR. These genetic variations might be associated with increasing or decreasing the incidence of OIIRR. It is important to note that all of the included genetic studies were only observational.

Polymorphisms in some with substantial evidence studies were reported as an indicator for a higher chance of more severe OIIRR; one of the most common is the IL polymorphism, where several studies reported its effect on the severity of the OIIRR, such as IL1RN polymorphism, IL-1A variations, IL-1B, IL-6, and IL1RN (39,136,24,29,32,35,33,26,41). other genetic polymorphisms expected to affect the OIIRR in the included studies are vitamin D receptor TaqI polymorphism rs3102724, polymorphism of the OPG gene, specific genotypes for P2RX7 SNP rs208294, polymorphism of RANK (rs12455775) and osteoprotegerin (24,25,36,28,30). Also, an increase in the incidence rate of OIIRR with X chromosome located genes in men: STAG 2 gene, stromal antigen two-gene, rs151184635 and RP1-30E17.2 gene, and marginal association with SSP1: rs11730582, P2RX7, and TNFRSF11A: rs8086340 (9). Lower evidence of genetic linkages were TNFRSF11A, stromal antigen two genes, microsatellite marker D18S64 (linked to TNFRSF11A) and RP1-30E17.2 gene (38,39,145).

Conversely, according to other low risks studies, some genes may have a protective effect, such as variations in the OPN gene and the P2RX7 gene, osteopontin (OPN) deficient subjects, polymorphisms of osteoprotegerin and IRAK1 (31,33,28,27).

Whilst other studies found there was no relationship between the severity of OIIRR and genetic variations in IL1b (rs1143634), P2RX7 (rs1718119), CASP1/ICE (rs530537), P2RX7 (rs208294), IL-1RN SNP (rs419598), polymorphisms in the RANKL gene, polymorphisms in TNFa and TNSALP, the haplotype of P2RX7 (rs208294 and rs1718119), SPP1 or TNFRSF11B (38,137,25,29,30).

Many articles discussing the genetic effect, both included and not included in our work, supported the belief of a possible relationship between genetic variations and OIIRR, which explains the difference in the amount of root resorption between individuals with other factors held constant.

### Limitations

Multiple aspects of this systematic review accentuate the limitations of the currently available literature dealing with the question posed.

Results of *in vitro* studies are generally questionable regarding their generalizability and applicability to the natural environment. For example, in some studies with the force application model device, direct contact between the glass and cells may generate an electrical charge and gas exchange. In addition, using different devices and software may also lead to variability in outcomes. Furthermore, using a two-dimensional or three-dimensional medium is questionable; some studies used both, showing different cellular and molecular responses (77). In addition, different methodologies, homemade devices, and software not verified by a standardized technology cause high variability in outcomes.

Most animal studies were unclear regarding the randomization and blinding of the assessors. Another big issue for the *in vivo* studies is the selection of radiographic techniques for reporting the OIIRR. As tooth resorption is a three-dimensional process, it may affect not only the length of the root, which is assigned as apical resorption (which is the reported outcome of most 2D radiographic studies) but may also exhibit itself as the reduction in thickness, cervical resorption, creation of coves, lacunae, and mini cracks. These increase with biomechanics application and cannot be detected by 2D radiographs (periapical (PA), panoramic, or cephalometric). Another drawback of the none standardized radiograph includes different magnification levels, angulation and superimposition of other structures, limiting the usefulness of this data (157), and PA results may be overestimated (158). In contrast, the 3D imaging (CBCT) and scanning electron microscope studies provide more relevant data on root resorption (159). Since these methods are becoming easier to access nowadays, this systematic review suggests that future *in vivo* observations should consider utilizing them.

Also, histological studies using a light microscope offer less information than scanning electron microscopes. Nevertheless, the main limitation of both methodologies is the fact that they can only be utilized with extracted teeth. Additionally, some studies report results by grouping outcomes, leading to generalizing results and data loss.

For human studies, it is well known that randomized clinical trials deliver the most evidence-based results: Due to our inclusion and exclusion criteria, we included only five randomized-controlled trials for this systematic review (129,130,131,45,23). Three additional papers did not comment on randomization methodology in their text and were considered nonrandomised clinical interventions (135,143,47). All other studies were either extended cohort or case-control studies; some exhibited a significant delay between conducting experiments and publication of results. And some used a four-point grading system (Malmgren Levander Index) or other indecies to classify the severity of OIIRR, without an exact numerical measurement, which decreased the ability for comparison. Nevertheless, more RCTs and observational studies in a controlled environment are needed to support this hypothesis.

Although these are exciting results of the genetic studies, the findings remain controversial. Newer evidence suggests that not only one SNP within a gene can act as an absolute determinant for the disease or increase the severity of the OIIRR, but other genetic factors such as (gene–gene interactions) and environmental and lifestyle risk factors (gene-lifestyle, gene-environment, and lifestyle interactions-environmental) may play a role (160).

Only a tiny portion of genes could be identified in genetic studies, and genetic variance may have structural variations, leading to difficulties in discovering the missing SNP risk factor unless the main contributing factors are fully understood. According to Schäfer *et al*., genetic-association studies have four main problems First, careful case selection is essential to minimise phenotypic heterogeneity. Also, sufficient participants should contribute to the study’s statistical power. Further, replication, which can be considered the gold standard, and the complete capturing of the genetic information is essential. If all these factors were applied, this should result in a significant finding, but unfortunately, many OIIRR genetic studies miss one or more (160). The small and underpowered SNPs’ studies available resulted in controversial and questionable findings.

Variability in methodology, sample sizes, and baseline characteristics may contribute to the observed high heterogeneity of included studies. Missing or unclear information around the randomization process, sample size calculation, blinding of the patient and/or assessors, and methodology on outcome measurements impact the risk of bias assessments and reduce the reproducibility of included studies.

We have generalized our research question to include mechanical forces. Therefore, we have not compared different treatment mechanics or interventions, even though the choice of the appliance with different force direction and strength may have a relevant impact on OIIRR incidence and severity; this can be considered a limitation of our work. Last, we refrained from performing a meta-analysis due to the high heterogeneity of the studies included regarding methodology, outcomes measures, and reporting strategies.

## Conclusions

1. Available evidence uniformly demonstrates a linkage between varying mechanical orthodontic forces and the occurrence of OIIRR.
2. Risks for increasing OIIRR should be viewed with caution, including occlusion, previous trauma, tooth shape, allergy, low bone turnover, and medication like corticosteroids, strontium ranelate, 4-hexylresorcinol and genetic polymorphism of IL-1B, IL-1RN, RANK, osteoprotegerin, and vitamin D receptor TaqI polymorphisms. Simultaneously, other factors seem to decrease the rate of OIIRR, such as oral contraceptives, baicalin, high caffeine, root-filled teeth, and polymorphism of osteoprotegerin and IRAK1.
3. Evidence from *in vitro* studies on different force types and mechanical regimes on HPDL cells, which affect biological behaviour and genetic expression, provided a possible explanation for their extracellular matrix gene expression and adhesion, osteotropic cytokines, growth factors, controlling PDLC osteoblastic/cementoblasts differentiation and proliferation capacity, and autophagy which provides a novel mechanism in the regulation of the clinical OTM process and root resorption.

Overall, this work highlights the need for genetic studies with an ‘-omics# basis for PDL cells under pressure. Further, this systematic review has also highlighted the shortcomings of currently available literature. Eligible studies were of high heterogeneity in methodology and often had a different risk of bias and small sample sizes, and therefore need to be interpreted with caution. More profound research is needed to understand the pathophysiological mechanisms underlying OIIRR, especially concerning the host response.

## Supplementary material

Supplementary material is available at *European Journal of Orthodontics* online.

Supplementary Table 1. Search terms.

Supplementary Table 2. List of excluded studies and justification.

Supplementary Table 3. Quality assessment of *in-vitro* studies (PICO1).

Supplementary Table 4. PICO 1A, studies’ summary

Supplementary Table 5. PICO 1, detailed study descriptions and outcomes.

Supplementary Table 6. PICO1 most upregulated genetic expression profile.

Supplementary Table 7. PICO1 most downregulated genetic expression profile.

Supplementary Table 8. Risk of bias in animal studies (SYRCLE Tool, PICO 2A and 2B).

Supplementary Table 9. PICO 2A, studies’ summary

Supplementary Table 10. PICO 2A, detailed studies description and outcomes.

Supplementary Table 11. PIECO 2B studies’ summary.

Supplementary Table 12. PICO 2B, detailed studies description and outcomes.

Supplementary Table 13. GRADE summary of findings for OIIRR outcome for animal studies PICO 2A and 2B.

Supplementary Table 14. Risk of bias human studies (PICO 3A and 3B).

Supplementary Table 15. PICO 3A, studies’ summary

Supplementary Table 16. PICO 3A, detailed study descriptions and outcomes.

Supplementary Table 17. PIECO 3B studies’ summary.

Supplementary Table 18. PICO 3B, detailed study descriptions and outcomes.

Supplementary Table 19. GRADE summary of findings for OIIRR outcome for human studies PICO 3A and 3B.

Supplementary Table 20. List of abbreviations.

cjad011_suppl_Supplementary_Table_S1

cjad011_suppl_Supplementary_Table_S2

cjad011_suppl_Supplementary_Table_S3

cjad011_suppl_Supplementary_Table_S4

cjad011_suppl_Supplementary_Table_S5

cjad011_suppl_Supplementary_Table_S6

cjad011_suppl_Supplementary_Table_S7

cjad011_suppl_Supplementary_Table_S8

cjad011_suppl_Supplementary_Table_S9

cjad011_suppl_Supplementary_Table_S10

cjad011_suppl_Supplementary_Table_S11

cjad011_suppl_Supplementary_Table_S12

cjad011_suppl_Supplementary_Table_S13

cjad011_suppl_Supplementary_Table_S14

cjad011_suppl_Supplementary_Table_S15

cjad011_suppl_Supplementary_Table_S16

cjad011_suppl_Supplementary_Table_S17

cjad011_suppl_Supplementary_Table_S18
