# Supplementary material for: Under pressure—mechanisms and risk factors for orthodontically induced inflammatory root resorption: a systematic review
Source: Eur J Orthod. 2023 Jun 27;45(5):612–26. doi: 10.1093/ejo/cjad011 (PMC10505745; doi:10.1093/ejo/cjad011)
Supplement: cjad011_suppl_Supplementary_Table_S1 [file cjad011_suppl_supplementary_table_s1.docx]

Supplemental Table 1: Search Terms

1.Embase through Ovid:

| **DataBase Search: Embase (Through Ovid)** | | | **Limit** |
| --- | --- | --- | --- |
| **PICO 1a** | #1 | exp *orthodontics/ OR exp *orthodontic procedure/ OR exp orthodontic tooth movement/ OR exp orthodontist/ OR exp orthodontic device/ OR  (Orthodontic* OR Periodont* OR tooth movement OR teeth movement OR root movement).mp | -Resource from: -Embase.  -English |
|  | #2 | exp biomechanics/ OR exp mechanical stress/ OR exp mechanics/ OR  mechan* force* OR orthodont* force* OR pressure OR strain OR stress OR mechanical stress OR compressi* OR tension OR biomechanics OR tensile OR pathophysiolog* OR mechanism OR physiology* OR biolog* OR mechanics.).mp. |  |
|  | #3 | exp *tooth disease/co, di, et, pc, si [Complication, Diagnosis, Etiology, Prevention, Side Effect] OR exp "tooth root"/ OR (root* resor?tion* OR root* crack* OR root* dissolution OR root* shortening OR root blunting OR root erosion OR tooth* resor?tion* OR tooth* crack* OR tooth* dissolution OR tooth* shortening OR tooth blunting OR tooth erosion OR teeth* resor?tion* OR teeth crack* OR teeth dissolution OR teeth shortening OR teeth blunting OR teeth erosion OR dent* resorption* OR dent* blunting OR dent* erosion OR dent* crack* OR dent* dissolution OR dent* shortening OR apex resorption* OR apex crack* OR apex dissolution OR apex shortening OR apex blunting OR apex erosion OR apical resor?tion* OR apical crack* OR apical dissolution OR apical shortening OR apical blunting OR apical erosion OR ortho* resor?tion* OR ortho* crack* OR ortho* dissolution OR ortho* shortening OR ortho* blunting OR ortho* erosion) |  |
|  | #4 | in vitro.mp. OR exp in vitro study/  OR exp preclinical study/ OR exp model/  cell culture.mp. OR exp cell culture/ OR  tissue culture.mp. OR exp tissue culture/ OR  exp genetics/ or exp molecular genetics/ OR  Molecular Biology.mp. or exp molecular biology/ OR  biomarkers.mp. or exp biological marker/ OR  exp tissue scaffold/ OR  (tooth movement model or cell* model* or tissue model* or culture model or scaffold or in vitro cell research or simulat* ortho* force*) .mp. |  |
|  | #5 | #1 AND #2 AND #3 AND #4 |  |

| **DataBase Search: Embase (Through Ovid)** | | | **Limit** |
| --- | --- | --- | --- |
| **PICO 1b** | #1 | exp *orthodontics/ OR exp *orthodontic procedure/ OR exp orthodontic tooth movement/ OR exp orthodontist/ OR exp orthodontic device/ OR  (Orthodontic* OR Periodont* OR tooth movement OR teeth movement OR root movement).mp | -Resource from: -Embase.  -English |
|  | #2 | risk factors.mp. or exp risk factor/ OR intrinsic factor/ or initiation factor/ OR systemic diseases.mp. or exp systemic disease/ OR exp drug therapy/ OR hormonal.mp. or exp hormonal therapy/ OR hormon.mp. OR risk factor* OR risk agent* OR risk effect* OR risk emergent* OR risk reaction OR risk outcome* OR affecting factor* OR affecting agent* OR affecting effect* OR affecting event OR affecting emergent OR affecting reaction OR affecting outcome* OR inducing factors OR inducing agent OR inducing effect OR inducing emergent OR inducing reaction OR inducing outcome OR related factor OR related agent OR related effect* OR related event* OR related emergent* OR related reaction* OR related outcome* OR increasing factor* OR increasing agent* OR increasing effects OR increasing event* OR increasing emergent OR increasing reaction* OR increasing outcome* OR decreasing factor* OR decreasing agent* OR decreasing effect* OR decreasing event* OR decreasing emergent OR decreasing reaction* OR decreasing outcome* OR modif* factor* OR modif* agent* OR modif* effect* OR modif* event* OR modif* emergent OR modif* reaction* OR modif* outcome* |  |
|  | #3 | exp *tooth disease/co, di, et, pc, si [Complication, Diagnosis, Etiology, Prevention, Side Effect] OR exp "tooth root"/ OR (root* resor?tion* OR root* crack* OR root* dissolution OR root* shortening OR root blunting OR root erosion OR tooth* resor?tion* OR tooth* crack* OR tooth* dissolution OR tooth* shortening OR tooth blunting OR tooth erosion OR teeth* resor?tion* OR teeth crack* OR teeth dissolution OR teeth shortening OR teeth blunting OR teeth erosion OR dent* resorption* OR dent* blunting OR dent* erosion OR dent* crack* OR dent* dissolution OR dent* shortening OR apex resorption* OR apex crack* OR apex dissolution OR apex shortening OR apex blunting OR apex erosion OR apical resor?tion* OR apical crack* OR apical dissolution OR apical shortening OR apical blunting OR apical erosion OR ortho* resor?tion* OR ortho* crack* OR ortho* dissolution OR ortho* shortening OR ortho* blunting OR ortho* erosion) .mp. |  |
|  | #4 | in vitro.mp. OR exp in vitro study/ OR exp preclinical study/ OR exp model/  cell culture.mp. OR exp cell culture/ OR tissue culture.mp. OR exp tissue culture/ OR exp genetics/ or exp molecular genetics/ OR Molecular Biology.mp. or exp molecular biology/ OR biomarkers.mp. or exp biological marker/ OR exp tissue scaffold/ OR (tooth movement model or cell* model* or tissue model* or culture model or scaffold or in vitro cell research or simulat* ortho* force*) .mp. |  |
|  | #5 | #1 AND #2 AND #3 AND #4 |  |

| **DataBase Search: Embase (Through Ovid)** | | | **Limit** |
| --- | --- | --- | --- |
| **2nd PICO a** | #1 | exp *orthodontics/ OR exp *orthodontic procedure/ OR exp orthodontic tooth movement/ OR exp orthodontist/ OR exp orthodontic device/ OR  (Orthodontic* OR Periodont* OR tooth movement OR teeth movement OR root movement).mp | -Resource from: -Embase.  -English  -animal  -animals studies |
|  | #2 | exp biomechanics/ OR exp mechanical stress/ OR exp mechanics/ OR  mechan* force* OR orthodont* force* OR pressure OR strain OR stress OR mechanical stress OR compressi* OR tension OR biomechanics OR tensile OR pathophysiolog* OR mechanism OR physiology* OR biolog* OR mechanics.).mp. |  |
|  | #3 | exp *tooth disease/co, di, et, pc, si [Complication, Diagnosis, Etiology, Prevention, Side Effect] OR exp "tooth root"/ OR (root* resor?tion* OR root* crack* OR root* dissolution OR root* shortening OR root blunting OR root erosion OR tooth* resor?tion* OR tooth* crack* OR tooth* dissolution OR tooth* shortening OR tooth blunting OR tooth erosion OR teeth* resor?tion* OR teeth crack* OR teeth dissolution OR teeth shortening OR teeth blunting OR teeth erosion OR dent* resorption* OR dent* blunting OR dent* erosion OR dent* crack* OR dent* dissolution OR dent* shortening OR apex resorption* OR apex crack* OR apex dissolution OR apex shortening OR apex blunting OR apex erosion OR apical resor?tion* OR apical crack* OR apical dissolution OR apical shortening OR apical blunting OR apical erosion OR ortho* resor?tion* OR ortho* crack* OR ortho* dissolution OR ortho* shortening OR ortho* blunting OR ortho* erosion) |  |
|  | #4 | #1 AND #2 AND #3 |  |

| **DataBase Search: Embase (Through Ovid)** | | | **Limit** |
| --- | --- | --- | --- |
| **2nd PICO b** | #1 | exp *orthodontics/ OR exp *orthodontic procedure/ OR exp orthodontic tooth movement/ OR exp orthodontist/ OR exp orthodontic device/ OR  (Orthodontic* OR Periodont* OR tooth movement OR teeth movement OR root movement).mp | -Resource from: -Embase.  -English  -animal  -animals studies |
|  | #2 | risk factors.mp. or exp risk factor/ OR intrinsic factor/ or initiation factor/ OR systemic diseases.mp. or exp systemic disease/ OR exp drug therapy/ OR hormonal.mp. or exp hormonal therapy/ OR hormon.mp. OR (risk factor* OR risk agent* OR risk effect* OR risk emergent* OR risk reaction OR risk outcome* OR affecting factor* OR affecting agent* OR affecting effect* OR affecting event OR affecting emergent OR affecting reaction OR affecting outcome* OR inducing factors OR inducing agent OR inducing effect OR inducing emergent OR inducing reaction OR inducing outcome OR related factor OR related agent OR related effect* OR related event* OR related emergent* OR related reaction* OR related outcome* OR increasing factor* OR increasing agent* OR increasing effects OR increasing event* OR increasing emergent OR increasing reaction* OR increasing outcome* OR decreasing factor* OR decreasing agent* OR decreasing effect* OR decreasing event* OR decreasing emergent OR decreasing reaction* OR decreasing outcome* OR modif* factor* OR modif* agent* OR modif* effect* OR modif* event* OR modif* emergent OR modif* reaction* OR modif* outcome*) .mp. |  |
|  | #3 | exp *tooth disease/co, di, et, pc, si [Complication, Diagnosis, Etiology, Prevention, Side Effect] OR exp "tooth root"/ OR (root* resor?tion* OR root* crack* OR root* dissolution OR root* shortening OR root blunting OR root erosion OR tooth* resor?tion* OR tooth* crack* OR tooth* dissolution OR tooth* shortening OR tooth blunting OR tooth erosion OR teeth* resor?tion* OR teeth crack* OR teeth dissolution OR teeth shortening OR teeth blunting OR teeth erosion OR dent* resorption* OR dent* blunting OR dent* erosion OR dent* crack* OR dent* dissolution OR dent* shortening OR apex resorption* OR apex crack* OR apex dissolution OR apex shortening OR apex blunting OR apex erosion OR apical resor?tion* OR apical crack* OR apical dissolution OR apical shortening OR apical blunting OR apical erosion OR ortho* resor?tion* OR ortho* crack* OR ortho* dissolution OR ortho* shortening OR ortho* blunting OR ortho* erosion) .mp. |  |
|  | #4 | #1 AND #2 AND #3 |  |
| 1608 after limitation 319 results remain. | | | |

| **Database Search: Embase (Through Ovid)** | | | **Limit** |
| --- | --- | --- | --- |
| **PICO 3a** | #1 | exp *orthodontics/ OR exp *orthodontic procedure/ OR exp orthodontic tooth movement/ OR exp orthodontist/ OR exp orthodontic device/ OR  (Orthodontic* OR Periodont* OR tooth movement OR teeth movement OR root movement).mp | -Resource from: -Embase.  -English  -human |
|  | #2 | exp biomechanics/ OR exp mechanical stress/ OR exp mechanics/ OR  mechan* force* OR orthodont* force* OR pressure OR strain OR stress OR mechanical stress OR compressi* OR tension OR biomechanics OR tensile OR pathophysiolog* OR mechanism OR physiology* OR biolog* OR mechanics.).mp. |  |
|  | #3 | exp *tooth disease/co, di, et, pc, si [Complication, Diagnosis, Etiology, Prevention, Side Effect] OR exp "tooth root"/ OR (root* resor?tion* OR root* crack* OR root* dissolution OR root* shortening OR root blunting OR root erosion OR tooth* resor?tion* OR tooth* crack* OR tooth* dissolution OR tooth* shortening OR tooth blunting OR tooth erosion OR teeth* resor?tion* OR teeth crack* OR teeth dissolution OR teeth shortening OR teeth blunting OR teeth erosion OR dent* resorption* OR dent* blunting OR dent* erosion OR dent* crack* OR dent* dissolution OR dent* shortening OR apex resorption* OR apex crack* OR apex dissolution OR apex shortening OR apex blunting OR apex erosion OR apical resor?tion* OR apical crack* OR apical dissolution OR apical shortening OR apical blunting OR apical erosion OR ortho* resor?tion* OR ortho* crack* OR ortho* dissolution OR ortho* shortening OR ortho* blunting OR ortho* erosion) |  |
|  | #4 | #1 AND #2 AND #3 |  |

| **DataBase Search: Embase (Through Ovid)** | | | **Limit** |
| --- | --- | --- | --- |
| **PICO 3b** | #1 | exp *orthodontics/ OR exp *orthodontic procedure/ OR exp orthodontic tooth movement/ OR exp orthodontist/ OR exp orthodontic device/ OR  (Orthodontic* OR Periodont* OR tooth movement OR teeth movement OR root movement).mp | -Resource from: -Embase.  -English  -Human |
|  | #2 | risk factors.mp. or exp risk factor/ OR intrinsic factor/ or initiation factor/ OR systemic diseases.mp. or exp systemic disease/ OR exp drug therapy/ OR hormonal.mp. or exp hormonal therapy/ OR hormon.mp. OR exp drug/ OR exp nutrition supplement/ OR exp dietary supplement/ OR (risk factor* OR risk agent* OR risk effect* OR risk emergent* OR risk reaction OR risk outcome* OR affecting factor* OR affecting agent* OR affecting effect* OR affecting event OR affecting emergent OR affecting reaction OR affecting outcome* OR inducing factors OR inducing agent OR inducing effect OR inducing emergent OR inducing reaction OR inducing outcome OR related factor OR related agent OR related effect* OR related event* OR related emergent* OR related reaction* OR related outcome* OR increasing factor* OR increasing agent* OR increasing effects OR increasing event* OR increasing emergent OR increasing reaction* OR increasing outcome* OR decreasing factor* OR decreasing agent* OR decreasing effect* OR decreasing event* OR decreasing emergent OR decreasing reaction* OR decreasing outcome* OR modif* factor* OR modif* agent* OR modif* effect* OR modif* event* OR modif* emergent OR modif* reaction* OR modif* outcome*) .mp. |  |
|  | #3 | exp *tooth disease/co, di, et, pc, si [Complication, Diagnosis, Etiology, Prevention, Side Effect] OR exp "tooth root"/ OR (root* resor?tion* OR root* crack* OR root* dissolution OR root* shortening OR root blunting OR root erosion OR tooth* resor?tion* OR tooth* crack* OR tooth* dissolution OR tooth* shortening OR tooth blunting OR tooth erosion OR teeth* resor?tion* OR teeth crack* OR teeth dissolution OR teeth shortening OR teeth blunting OR teeth erosion OR dent* resorption* OR dent* blunting OR dent* erosion OR dent* crack* OR dent* dissolution OR dent* shortening OR apex resorption* OR apex crack* OR apex dissolution OR apex shortening OR apex blunting OR apex erosion OR apical resor?tion* OR apical crack* OR apical dissolution OR apical shortening OR apical blunting OR apical erosion OR ortho* resor?tion* OR ortho* crack* OR ortho* dissolution OR ortho* shortening OR ortho* blunting OR ortho* erosion) .mp. |  |
|  | #4 | #1 AND #2 AND #3 |  |
| 827 after limitation 303 results remains | | | |

2.Cochrane:

| **PICO**  **1a** | #1 | MeSH descriptor: [Orthodontics] explode all trees | English  All dates  All years |  |
| --- | --- | --- | --- | --- |
|  | #2 | MeSH descriptor: [Tooth Movement Techniques] explode all trees |  |  |
|  | #3 | MeSH descriptor: [Periodontics] explode all trees |  |  |
|  | #4 | MeSH descriptor: [Dentistry] explode all trees |  |  |
|  | #5 | Orthodontic* OR Periodont* OR tooth movement OR teeth movement OR root movement |  |  |
|  | #6 | MeSH descriptor: [Biomechanical Phenomena] explode all trees |  |  |
|  | #7 | MeSH descriptor: [Mechanical Phenomena] explode all trees |  |  |
|  | #8 | MeSH descriptor: [Stress, Mechanical] explode all trees |  |  |
|  | #9 | mechan* force* OR orthodont* force* OR pressure OR strain OR stress OR mechanical stress OR compressi* OR tension OR biomechanics OR tensile OR pathophysiolog* OR mechanism OR physiology* OR biolog* OR mechanics |  |  |
|  | #10 | MeSH descriptor: [Tooth Resorption] explode all trees |  |  |
|  | #11 | MeSH descriptor: [Root Resorption] explode all trees |  |  |
|  | #12 | MeSH descriptor: [Pathology, Oral] explode all trees |  |  |
|  | #13 | MeSH descriptor: [Tooth Diseases] explode all trees |  |  |
|  | #14 | root* resor?tion* OR root* crack* OR root* dissolution OR root* shortening OR root blunting OR root erosion OR apex resorption* OR apex crack* OR apex dissolution OR apex shortening OR apex blunting OR apex erosion OR apical resor?tion* OR apical crack* OR apical dissolution OR apical shortening OR apical blunting |  |  |
|  | #15 | MeSH descriptor: [In Vitro Techniques] explode all trees |  |  |
|  | #16 | MeSH descriptor: [Cells, Cultured] explode all trees |  |  |
|  | #17 | MeSH descriptor: [Cytological Techniques] explode all trees |  |  |
|  | #18 | MeSH descriptor: [Dental Models] explode all trees |  |  |
|  | #19 | cytology OR Cell stud* OR culture* OR molecular |  |  |
|  | #20 | #1 OR #2 OR #3 OR #4 OR #5 |  |  |
|  | #21 | #6 OR #7 OR #8 OR #9 |  |  |
|  | #22 | #10 OR #11 OR #12 OR #13 OR #14 |  |  |
|  | #23 | #15 OR #16 OR #17 OR #18 OR #19 |  |  |
|  | #24 | #20 AND #21 AND #22 AND #23 |  |  |

| **PICO**  **1b** | #1 | MeSH descriptor: [Orthodontics] explode all trees | English  All dates  All years |  |
| --- | --- | --- | --- | --- |
|  | #2 | MeSH descriptor: [Tooth Movement Techniques] explode all trees |  |  |
|  | #3 | MeSH descriptor: [Periodontics] explode all trees |  |  |
|  | #4 | MeSH descriptor: [Dentistry] explode all trees |  |  |
|  | #5 | Orthodontic* OR Periodont* OR tooth movement OR teeth movement OR root movement |  |  |
|  | #6 | MeSH descriptor: [Long Term Adverse Effects] explode all trees |  |  |
|  | #7 | MeSH descriptor: [Risk Factors] explode all trees |  |  |
|  | #8 | risk factor* OR risk agent* OR risk effect* OR risk outcome* OR affecting factor* OR affecting agent* OR affecting outcome* OR inducing factors OR inducing agent OR inducing effect OR related factor OR related agent OR related effect* OR related reaction* OR related outcome* |  |  |
|  | #9 | MeSH descriptor: [Tooth Resorption] explode all trees |  |  |
|  | #10 | MeSH descriptor: [Root Resorption] explode all trees |  |  |
|  | #11 | MeSH descriptor: [Pathology, Oral] explode all trees |  |  |
|  | #12 | MeSH descriptor: [Tooth Diseases] explode all trees |  |  |
|  | #13 | root* resor?tion* OR root* crack* OR root* dissolution OR root* shortening OR root blunting OR root erosion OR apex resorption* OR apex crack* OR apex dissolution OR apex shortening OR apex blunting OR apex erosion OR apical resor?tion* OR apical crack* OR apical dissolution OR apical shortening OR apical blunting |  |  |
|  | #14 | MeSH descriptor: [In Vitro Techniques] explode all trees |  |  |
|  | #15 | MeSH descriptor: [Cells, Cultured] explode all trees |  |  |
|  | #16 | MeSH descriptor: [Cytological Techniques] explode all trees |  |  |
|  | #17 | MeSH descriptor: [Dental Models] explode all trees |  |  |
|  | #18 | cytology OR Cell stud* OR culture* OR molecular |  |  |
|  | #19 | #1 OR #2 OR #3 OR #4 OR #5 |  |  |
|  | #20 | #6 OR #7 OR #8 |  |  |
|  | #21 | #9 OR #10 OR #11 OR #12 OR #13 |  |  |
|  | #22 | #14 OR #15 OR #16 OR #17 OR #18 |  |  |
|  | #23 | #19 AND #20 AND #21 AND #22 |  |  |

| **PICO**  **2a** | #1 | MeSH descriptor: [Orthodontics] explode all trees | Language: English  All dates  All years |  |
| --- | --- | --- | --- | --- |
|  | #2 | MeSH descriptor: [Tooth Movement Techniques] explode all trees |  |  |
|  | #3 | MeSH descriptor: [Periodontics] explode all trees |  |  |
|  | #4 | MeSH descriptor: [Dentistry] explode all trees |  |  |
|  | #5 | MeSH descriptor: [Biomechanical Phenomena] explode all trees |  |  |
|  | #6 | MeSH descriptor: [Mechanical Phenomena] explode all trees |  |  |
|  | #7 | MeSH descriptor: [Long Term Adverse Effects] explode all trees |  |  |
|  | #8 | MeSH descriptor: [Stress, Mechanical] explode all trees |  |  |
|  | #9 | MeSH descriptor: [Mechanics] explode all trees |  |  |
|  | #10 | MeSH descriptor: [Tooth Resorption] explode all trees |  |  |
|  | #11 | MeSH descriptor: [Root Resorption] explode all trees |  |  |
|  | #12 | MeSH descriptor: [Pathology, Oral] explode all trees |  |  |
|  | #13 | MeSH descriptor: [Tooth Diseases] explode all trees |  |  |
|  | #14 | MeSH descriptor: [Animals] explode all trees |  |  |
|  | #15 | MeSH descriptor: [Animal Experimentation] explode all trees |  |  |
|  | #16 | MeSH descriptor: [Models, Animal] explode all trees |  |  |
|  | #17 | #1 OR #2 OR #3 OR #4 OR #5 |  |  |
|  | #18 | #4 OR #5 OR #6 OR #7 OR #8 OR #9 |  |  |
|  | #19 | #10 OR #11 OR #12 OR #13 |  |  |
|  | #20 | #14 OR #15 OR #16 |  |  |
|  | #21 | #17 AND #18 AND #19 AND #20 |  |  |
|  |  | 593 results retrived |  |  |

| **PICO**  **2b** | #1 | MeSH descriptor: [Orthodontics] explode all trees | English  All dates  All years |  |
| --- | --- | --- | --- | --- |
|  | #2 | MeSH descriptor: [Tooth Movement Techniques] explode all trees |  |  |
|  | #3 | MeSH descriptor: [Periodontics] explode all trees |  |  |
|  | #4 | MeSH descriptor: [Dentistry] explode all trees |  |  |
|  | #5 | Orthodontic* OR tooth movement OR teeth movement OR root movement |  |  |
|  | #6 | MeSH descriptor: [Long Term Adverse Effects] explode all trees |  |  |
|  | #7 | MeSH descriptor: [Risk Factors] explode all trees |  |  |
|  | #8 | risk factor* OR risk agent* OR risk effect* OR risk outcome* OR affecting factor* OR affecting agent* OR affecting outcome* OR inducing factors OR inducing agent OR inducing effect OR related factor OR related agent OR related effect* OR related reaction* OR related outcome* |  |  |
|  | #9 | MeSH descriptor: [Tooth Resorption] explode all trees |  |  |
|  | #10 | MeSH descriptor: [Root Resorption] explode all trees |  |  |
|  | #11 | MeSH descriptor: [Pathology, Oral] explode all trees |  |  |
|  | #12 | MeSH descriptor: [Tooth Diseases] explode all trees |  |  |
|  | #13 | root* resor?tion* OR root* crack* OR root* dissolution OR root* shortening OR root blunting OR root erosion OR apex resorption* OR apex crack* OR apex dissolution OR apex shortening OR apex blunting OR apex erosion OR apical resor?tion* OR apical crack* OR apical dissolution OR apical shortening OR apical blunting |  |  |
|  | #14 | MeSH descriptor: [Animals] explode all trees |  |  |
|  | #15 | MeSH descriptor: [Animal Experimentation] explode all trees |  |  |
|  | #16 | MeSH descriptor: [Models, Animal] explode all trees |  |  |
|  | #17 | MeSH descriptor: [In Vitro Techniques] explode all trees |  |  |
|  | #18 | MeSH descriptor: [Cell Culture Techniques] explode all trees |  |  |
|  | #19 | MeSH descriptor: [Cytological Techniques] explode all trees |  |  |
|  | #20 | MeSH descriptor: [Dental Models] explode all trees |  |  |
|  | #21 | cytology OR Cell stud* OR culture* OR molecular |  |  |
|  | #22 | Human research* OR clinical research or clinical stud* or patient* or human stud* OR person* |  |  |
|  | #23 | #1 OR #2 OR #3 OR #4 OR #5 |  |  |
|  | #24 | #6 OR #7 OR #8 |  |  |
|  | #25 | #9 OR #10 OR #11 OR #12 OR #13 |  |  |
|  | #26 | #14 OR #15 OR #16 |  |  |
|  | #27 | #17 OR #18 OR #19 OR #20 OR 21 OR #22 |  |  |
|  | #28 | #23 AND #24 AND #25 AND #26 |  |  |
|  | #29 | #28 NOT #27 |  |  |
|  |  | 500 trials results |  |  |

| **DataBase Search: CENTRAL (The Cochrane Central Register of Controlled Trials(** | | | **Limit** |
| --- | --- | --- | --- |
| **PICO**  **3a** | #1 | MeSH descriptor: [Orthodontics] explode all trees | English  All dates  All years |
|  | #2 | MeSH descriptor: [Tooth Movement Techniques] explode all trees |  |
|  | #3 | MeSH descriptor: [Periodontics] explode all trees |  |
|  | #4 | MeSH descriptor: [Biomechanical Phenomena] explode all trees |  |
|  | #5 | MeSH descriptor: [Mechanical Phenomena] explode all trees |  |
|  | #6 | MeSH descriptor: [Long Term Adverse Effects] explode all trees |  |
|  | #7 | MeSH descriptor: [Stress, Mechanical] explode all trees |  |
|  | #8 | MeSH descriptor: [Mechanics] explode all trees |  |
|  | #9 | MeSH descriptor: [Tooth Resorption] explode all trees |  |
|  | #10 | MeSH descriptor: [Root Resorption] explode all trees |  |
|  | #11 | MeSH descriptor: [Pathology, Oral] explode all trees |  |
|  | #13 | #1 OR #2 OR #3 OR #4 OR #5 |  |
|  | #14 | #6 OR #7 OR #8 |  |
|  | #15 | #9 OR #10 OR #11 |  |
|  | #16 | #13 AND #14 AND #15 |  |
|  |  | 261 Results |  |

| **PICO**  **3b** | #1 | MeSH descriptor: [Orthodontics] explode all trees | English  All dates  All years |
| --- | --- | --- | --- |
|  | #2 | MeSH descriptor: [Tooth Movement Techniques] explode all trees |  |
|  | #3 | MeSH descriptor: [Biomechanical Phenomena] explode all trees |  |
|  | #4 | MeSH descriptor: [Mechanical Phenomena] explode all trees |  |
|  | #5 | MeSH descriptor: [Periodontics] explode all trees |  |
|  | #6 | MeSH descriptor: [Long Term Adverse Effects] explode all trees |  |
|  | #7 | MeSH descriptor: [Risk Factors] explode all trees |  |
|  | #8 | MeSH descriptor: [Disease] explode all trees |  |
|  | #9 | MeSH descriptor: [Hormones] explode all trees |  |
|  | #10 | MeSH descriptor: [Pharmaceutical Preparations] explode all trees |  |
|  | #11 | MeSH descriptor: [Dietary Supplements] explode all trees |  |
|  | #12 | MeSH descriptor: [Tooth Resorption] explode all trees |  |
|  | #13 | MeSH descriptor: [Root Resorption] explode all trees |  |
|  | #14 | MeSH descriptor: [Pathology, Oral] explode all trees |  |
|  | #15 | MeSH descriptor: [Tooth Diseases] explode all trees |  |
|  | #16 | #1 OR #2 OR #3 OR #4 OR #5 |  |
|  | #17 | #6 OR #7 OR #8 OR #9 OR #10 OR #11 |  |
|  | #18 | #12 OR #13 OR #14 OR #15 |  |
|  | #19 | #16 AND #17 AND #18 | 462 results |

3. Pubmed:

| **PICO 1a** | #1 | (("Cell Culture Techniques"[Mesh]) OR ("Primary Cell Culture"[Mesh]) OR ("Genetics"[Mesh]) OR ("Molecular Biology"[Mesh]) OR ("Tissue Culture Techniques"[Mesh]) OR (in vitro[MeSH Terms]) OR (molecul*) OR (in vitro) or (genetic*) OR (biolog*) OR (cell* Culture) OR (tissue* Culture) OR (biomarker) OR (tooth movement model) OR (cell* model*) OR (tissue model*) OR (culture model) OR (scaffold) OR (in vitro cell research) OR (simulat* ortho* force*)) | Language: English |
| --- | --- | --- | --- |
|  | #2 | (("Orthodontics/adverse effects"[Mesh]) OR ("Orthodontics/complications"[Mesh]) OR ("Orthodontics/diagnosis"[Mesh]) OR ("Orthodontics/methods"[Mesh]) OR ("Orthodontics/prevention and control"[Mesh]) OR ("Orthodontics/therapeutic use"[Mesh]) OR ("Orthodontics/therapy"[Mesh]) OR ("Orthodontics/trends"[Mesh]) OR ("Orthodontics, Corrective/adverse effects"[Mesh]) OR ("Orthodontics, Corrective/classification"[Mesh]) OR ("Orthodontics, Corrective/methods"[Mesh]) OR ("Orthodontics, Corrective/pharmacology"[Mesh]) OR ("Orthodontics, Corrective/prevention and control"[Mesh]) OR ("Orthodontics, Corrective/standards"[Mesh]) OR ("Orthodontics, Corrective/therapeutic use"[Mesh]) OR ("Orthodontics, Corrective/therapy"[Mesh]) OR ("Orthodontics, Corrective/trends"[Mesh]) OR ("Tooth Movement Techniques/adverse effects"[Mesh]) OR ("Tooth Movement Techniques/classification"[Mesh]) OR ("Tooth Movement Techniques/ethics"[Mesh]) OR ("Tooth Movement Techniques/instrumentation"[Mesh]) OR ("Tooth Movement Techniques/methods"[Mesh]) OR ("Tooth Movement Techniques/pharmacology"[Mesh]) OR ("Tooth Movement Techniques/prevention and control"[Mesh]) OR ("Tooth Movement Techniques/standards"[Mesh]) OR ("Tooth Movement Techniques/statistics and numerical data"[Mesh]) OR ("Tooth Movement Techniques/therapeutic use"[Mesh]) OR ("Tooth Movement Techniques/therapy"[Mesh]) OR ("Tooth Movement Techniques/trends"[Mesh]) OR (orthodon*) OR (periodont*) OR (tooth movement) OR (Teeth Movement*)) |  |
|  | #3 | (("Long Term Adverse Effects/chemically induced"[Mesh]) OR ("Long Term Adverse Effects/classification"[Mesh]) OR ("Long Term Adverse Effects/analysis"[Mesh]) OR ("Long Term Adverse Effects/diagnosis"[Mesh]) OR ("Long Term Adverse Effects/pathology"[Mesh]) OR ("Long Term Adverse Effects/physiology"[Mesh]) OR ("Long Term Adverse Effects/physiopathology"[Mesh]) OR ("Long Term Adverse Effects/prevention and control"[Mesh]) OR ("Long Term Adverse Effects/therapy"[Mesh]) OR ("adverse effects" [Subheading]) OR ("Biomechanical Phenomena/chemistry"[Mesh]) OR ("Biomechanical Phenomena/classification"[Mesh]) OR ("Biomechanical Phenomena/drug effects"[Mesh]) OR ("Biomechanical Phenomena/etiology"[Mesh]) OR ("Biomechanical Phenomena/genetics"[Mesh]) OR ("Biomechanical Phenomena/methods" [Mesh]) OR ("Biomechanical Phenomena/physiology"[Mesh]) OR ("Biomechanical Phenomena/standards"[Mesh]) OR ("Biomechanical Phenomena/statistics and numerical data"[Mesh]) OR ("Biomechanical Phenomena/trends"[Mesh]) OR (inflammatory) OR (external) OR (induce*) OR (associated) OR (complicat*) OR (mechanism) OR (pathophysiolog*) Or (physiology*) OR (biolog*)) |  |
|  | #4 | ((mechan* force*) OR (orthodont* force*) OR (force*) (pressure) OR (strain) OR (stress) OR (compressi*) OR (tension) OR (Biomechanics) OR (tensile)) |  |
|  | #5 | (("Root Resorption"[Mesh]) OR ("Tooth Resorption"[Mesh]) OR (root* resor?tion*) OR (root* crack*) OR (root* dissolution) OR (root* shortening) OR (root blunting) OR (root erosion) OR (tooth* resor?tion*) OR (tooth* crack*) OR (tooth* dissolution) OR (tooth* shortening) OR (tooth blunting) OR (tooth erosion) OR (teeth* resor?tion*) OR (teeth crack*) OR (teeth dissolution) OR (teeth shortening) OR (teeth blunting) OR (teeth erosion) OR (dent* resorption*) OR (dent* blunting) OR (dent* erosion) OR (dent* crack*) OR (dent* dissolution) OR (dent* shortening) OR (apex resorption*) OR (apex crack*) OR (apex dissolution) OR (apex shortening) OR (apex blunting) OR (apex erosion) OR (apical resor?tion*) OR (apical crack*) OR (apical dissolution) OR (apical shortening) OR (apical blunting) OR (apical erosion) OR (ortho* resor?tion*) OR (ortho* crack*) OR (ortho* dissolution) OR (ortho* shortening) OR (ortho* blunting) OR (ortho* erosion)) |  |
|  | #6 | ((maxillary sinus[MeSH Terms]) OR (sinus[Title/Abstract]) OR (case reports[MeSH Terms]) OR (case study[Title/Abstract]) OR (case studies[Title/Abstract]) OR (case series[Title/Abstract]) OR ("Systematic Review" [Publication Type]) OR (orthop*[Title/Abstract]) OR (zygomatic*[Title/Abstract]) OR (syndrome[Title/Abstract]) OR (osteonecrosis[Title/Abstract]) OR (osteoradionecrosis[Title/Abstract]) OR (osteoclastogenesis[Title/Abstract]) OR (bleaching[Title/Abstract]) OR (whiten*[Title/Abstract]) OR (traumat*[Title/Abstract]) OR (surg*[Title/Abstract]) OR (prothetic*[Title/Abstract]) OR (peridont* lesion[Title/Abstract]) Or (internal resorption[Title/Abstract]) OR (screw*[Title/Abstract]) OR (periimplant*[Title/Abstract]) OR (peri-implant*[Title/Abstract]) OR (reimplant[Title/Abstract]) OR (implant*[Title/Abstract]) OR (cleft lip[Title/Abstract]) OR (cleft palate[Title/Abstract]) OR (systematic review[Title/Abstract]) OR(in vivo[Title/Abstract])) |  |
|  | #7 | #1 AND #2 AND #3 AND #4 AND #5 NOT #6 |  |

| **PICO 1b** | #1 | (("Cell Culture Techniques"[Mesh]) OR ("Primary Cell Culture"[Mesh]) OR ("Genetics"[Mesh]) OR ("Molecular Biology"[Mesh]) OR ("Tissue Culture Techniques"[Mesh]) OR (in vitro[MeSH Terms]) OR (molecul*) OR (in vitro) or (genetic*) OR (biolog*) OR (cell* Culture) OR (tissue* Culture) OR (biomarker) OR (tooth movement model) OR (cell* model*) OR (tissue model*) OR (culture model) OR (scaffold) OR (in vitro cell research) OR (simulat* ortho* force*)) | Language: English |
| --- | --- | --- | --- |
|  | #2 | (("Orthodontics/adverse effects"[Mesh]) OR ("Orthodontics/complications"[Mesh]) OR ("Orthodontics/diagnosis"[Mesh]) OR ("Orthodontics/methods"[Mesh]) OR ("Orthodontics/prevention and control"[Mesh]) OR ("Orthodontics/therapeutic use"[Mesh]) OR ("Orthodontics/therapy"[Mesh]) OR ("Orthodontics/trends"[Mesh]) OR ("Orthodontics, Corrective/adverse effects"[Mesh]) OR ("Orthodontics, Corrective/classification"[Mesh]) OR ("Orthodontics, Corrective/methods"[Mesh]) OR ("Orthodontics, Corrective/pharmacology"[Mesh]) OR ("Orthodontics, Corrective/prevention and control"[Mesh]) OR ("Orthodontics, Corrective/standards"[Mesh]) OR ("Orthodontics, Corrective/therapeutic use"[Mesh]) OR ("Orthodontics, Corrective/therapy"[Mesh]) OR ("Orthodontics, Corrective/trends"[Mesh]) OR ("Tooth Movement Techniques/adverse effects"[Mesh]) OR ("Tooth Movement Techniques/classification"[Mesh]) OR ("Tooth Movement Techniques/ethics"[Mesh]) OR ("Tooth Movement Techniques/instrumentation"[Mesh]) OR ("Tooth Movement Techniques/methods"[Mesh]) OR ("Tooth Movement Techniques/pharmacology"[Mesh]) OR ("Tooth Movement Techniques/prevention and control"[Mesh]) OR ("Tooth Movement Techniques/standards"[Mesh]) OR ("Tooth Movement Techniques/statistics and numerical data"[Mesh]) OR ("Tooth Movement Techniques/therapeutic use"[Mesh]) OR ("Tooth Movement Techniques/therapy"[Mesh]) OR ("Tooth Movement Techniques/trends"[Mesh]) OR (orthodon*) OR (periodont*) OR (tooth movement) OR (Teeth Movement*)) |  |
|  | #3 | (("Long Term Adverse Effects/chemically induced"[Mesh]) OR ("Long Term Adverse Effects/classification"[Mesh]) OR ("Long Term Adverse Effects/analysis"[Mesh]) OR ("Long Term Adverse Effects/diagnosis"[Mesh]) OR ("Long Term Adverse Effects/pathology"[Mesh]) OR ("Long Term Adverse Effects/physiology"[Mesh]) OR ("Long Term Adverse Effects/physiopathology"[Mesh]) OR ("Long Term Adverse Effects/prevention and control"[Mesh]) OR ("Long Term Adverse Effects/therapy"[Mesh]) OR ("adverse effects" [Subheading]) OR ("Biomechanical Phenomena/chemistry"[Mesh]) OR ("Biomechanical Phenomena/classification"[Mesh]) OR ("Biomechanical Phenomena/drug effects"[Mesh]) OR ("Biomechanical Phenomena/etiology"[Mesh]) OR ("Biomechanical Phenomena/genetics"[Mesh]) OR ("Biomechanical Phenomena/methods" [Mesh]) OR ("Biomechanical Phenomena/physiology"[Mesh]) OR ("Biomechanical Phenomena/standards"[Mesh]) OR ("Biomechanical Phenomena/statistics and numerical data"[Mesh]) OR ("Biomechanical Phenomena/trends"[Mesh]) OR (inflammatory) OR (external) OR (induce*) OR (associated) OR (complicat*) OR (mechanism) OR (pathophysiolog*) Or (physiology*) OR (biolog*)) |  |
|  | #4 | (("Risk Factors"[Mesh]) OR (risk factor*)) OR (risk agent*) OR (risk effect*) OR (risk emergent*) OR (risk reaction) OR (risk outcome*) OR (affecting factor*) OR (affecting agent*) OR (affecting effect*) OR ("affecting event") OR (affecting emergent) OR (affecting reaction) OR (affecting outcome*) OR (inducing factors) OR (inducing agent) OR (inducing effect) OR (inducing emergent) OR (inducing reaction) OR (inducing outcome) OR (related factor) OR (related agent) OR (related effect*) OR (related event*) OR (related emergent*) OR (related reaction*) OR (related outcome*) OR (increasing factor*) OR (increasing agent*) OR (increasing effects) OR (increasing event*) OR (increasing emergent) OR (increasing reaction*) OR (increasing outcome*) OR (decreasing factor*) OR (decreasing agent*) OR (decreasing effect*) OR (decreasing event*) OR (decreasing emergent) OR (decreasing reaction*) OR (decreasing outcome*) OR (modif* factor*) OR (modif* agent*) OR (modif* effect*) OR (modif* event*) OR (modif* emergent) OR (modif* reaction*) OR (modif* outcome*)) |  |
|  | #5 | (("Root Resorption"[Mesh]) OR ("Tooth Resorption"[Mesh]) OR (root* resor?tion*) OR (root* crack*) OR (root* dissolution) OR (root* shortening) OR (root blunting) OR (root erosion) OR (tooth* resor?tion*) OR (tooth* crack*) OR (tooth* dissolution) OR (tooth* shortening) OR (tooth blunting) OR (tooth erosion) OR (teeth* resor?tion*) OR (teeth crack*) OR (teeth dissolution) OR (teeth shortening) OR (teeth blunting) OR (teeth erosion) OR (dent* resorption*) OR (dent* blunting) OR (dent* erosion) OR (dent* crack*) OR (dent* dissolution) OR (dent* shortening) OR (apex resorption*) OR (apex crack*) OR (apex dissolution) OR (apex shortening) OR (apex blunting) OR (apex erosion) OR (apical resor?tion*) OR (apical crack*) OR (apical dissolution) OR (apical shortening) OR (apical blunting) OR (apical erosion) OR (ortho* resor?tion*) OR (ortho* crack*) OR (ortho* dissolution) OR (ortho* shortening) OR (ortho* blunting) OR (ortho* erosion)) |  |
|  | #6 | ((maxillary sinus[MeSH Terms]) OR (sinus[Title/Abstract]) OR (case reports[MeSH Terms]) OR (case study[Title/Abstract]) OR (case studies[Title/Abstract]) OR (case series[Title/Abstract]) OR ("Systematic Review" [Publication Type]) OR (orthop*[Title/Abstract]) OR (zygomatic*[Title/Abstract]) OR (syndrome[Title/Abstract]) OR (osteonecrosis[Title/Abstract]) OR (osteoradionecrosis[Title/Abstract]) OR (osteoclastogenesis[Title/Abstract]) OR (bleaching[Title/Abstract]) OR (whiten*[Title/Abstract]) OR (traumat*[Title/Abstract]) OR (surg*[Title/Abstract]) OR (prothetic*[Title/Abstract]) OR (peridont* lesion[Title/Abstract]) Or (internal resorption[Title/Abstract]) OR (screw*[Title/Abstract]) OR (periimplant*[Title/Abstract]) OR (peri-implant*[Title/Abstract]) OR (reimplant[Title/Abstract]) OR (implant*[Title/Abstract]) OR (cleft lip[Title/Abstract]) OR (cleft palate[Title/Abstract]) OR (systematic review[Title/Abstract]) OR(in vivo[Title/Abstract])) |  |
|  | #7 | #1 AND #2 AND #3 AND #4 AND #5 NOT #6 |  |

| **PICO**  **2a** | #1 | (("Animal Experimentation/adverse effects"[Mesh]) OR (animal experimentation[MeSH]) OR (animal model[MeSH]) OR (animal experiment [MeSH]) OR (animal trial[Title/Abstract]) OR (animal stud*[Title/Abstract]) OR ("Animal Experimentation/education"[Mesh]) OR ("Animal Experimentation/methods"[Mesh]) OR ("Animal Experimentation/pharmacology"[Mesh]) OR ("Animal Experimentation/standards"[Mesh]) OR ("Animal Experimentation/therapeutic use"[Mesh]) OR ("Animal Experimentation/veterinary"[Mesh]) OR ( "Animals/adverse effects"[Mesh]) OR ("Animals/classification"[Mesh]) OR ("Animals/complications"[Mesh]) OR ("Animals/diagnosis"[Mesh]) OR ("Animals/drug effects"[Mesh]) OR ("Animals/drug therapy"[Mesh]) OR ("Animals/genetics"[Mesh]) OR ("Animals/pathogenicity"[Mesh]) OR ("Animals/pharmacokinetics"[Mesh]) OR ("Animals/pharmacology"[Mesh]) OR ("Animals/physiopathology"[Mesh]) OR ("Animals/poisoning"[Mesh]) OR ("Animals/prevention and control"[Mesh]) OR ("Animals/therapeutic use"[Mesh]) OR ("Animals/therapy"[Mesh]) OR ("Animals/trends"[Mesh]) OR ("Animals/veterinary"[Mesh]) OR (dog[MeSH]) OR (mouse[Title/Abstract]) OR (mice[Title/Abstract]) OR (dog*[Title/Abstract]) OR (animal disease model[Title/Abstract])) | Language: English  Species Other Animals |
| --- | --- | --- | --- |
|  | #2 | (("Orthodontics/adverse effects"[Mesh]) OR ("Orthodontics/complications"[Mesh]) OR ("Orthodontics/diagnosis"[Mesh]) OR ("Orthodontics/methods"[Mesh]) OR ("Orthodontics/prevention and control"[Mesh]) OR ("Orthodontics/therapeutic use"[Mesh]) OR ("Orthodontics/therapy"[Mesh]) OR ("Orthodontics/trends"[Mesh]) OR ("Orthodontics, Corrective/adverse effects"[Mesh]) OR ("Orthodontics, Corrective/classification"[Mesh]) OR ("Orthodontics, Corrective/methods"[Mesh]) OR ("Orthodontics, Corrective/pharmacology"[Mesh]) OR ("Orthodontics, Corrective/prevention and control"[Mesh]) OR ("Orthodontics, Corrective/standards"[Mesh]) OR ("Orthodontics, Corrective/therapeutic use"[Mesh]) OR ("Orthodontics, Corrective/therapy"[Mesh]) OR ("Orthodontics, Corrective/trends"[Mesh]) OR ("Tooth Movement Techniques/adverse effects"[Mesh]) OR ("Tooth Movement Techniques/classification"[Mesh]) OR ("Tooth Movement Techniques/ethics"[Mesh]) OR ("Tooth Movement Techniques/instrumentation"[Mesh]) OR ("Tooth Movement Techniques/methods"[Mesh]) OR ("Tooth Movement Techniques/pharmacology"[Mesh]) OR ("Tooth Movement Techniques/prevention and control"[Mesh]) OR ("Tooth Movement Techniques/standards"[Mesh]) OR ("Tooth Movement Techniques/statistics and numerical data"[Mesh]) OR ("Tooth Movement Techniques/therapeutic use"[Mesh]) OR ("Tooth Movement Techniques/therapy"[Mesh]) OR ("Tooth Movement Techniques/trends"[Mesh]) OR (orthodon*) OR (periodont*) OR (tooth movement) OR (Teeth Movement*)) |  |
|  | #3 | (("Long Term Adverse Effects/chemically induced"[Mesh]) OR ("Long Term Adverse Effects/classification"[Mesh]) OR ("Long Term Adverse Effects/analysis"[Mesh]) OR ("Long Term Adverse Effects/diagnosis"[Mesh]) OR ("Long Term Adverse Effects/pathology"[Mesh]) OR ("Long Term Adverse Effects/physiology"[Mesh]) OR ("Long Term Adverse Effects/physiopathology"[Mesh]) OR ("Long Term Adverse Effects/prevention and control"[Mesh]) OR ("Long Term Adverse Effects/therapy"[Mesh]) OR ("adverse effects" [Subheading]) OR ("Biomechanical Phenomena/chemistry"[Mesh]) OR ("Biomechanical Phenomena/classification"[Mesh]) OR ("Biomechanical Phenomena/drug effects"[Mesh]) OR ("Biomechanical Phenomena/etiology"[Mesh]) OR ("Biomechanical Phenomena/genetics"[Mesh]) OR ("Biomechanical Phenomena/methods" [Mesh]) OR ("Biomechanical Phenomena/physiology"[Mesh]) OR ("Biomechanical Phenomena/standards"[Mesh]) OR ("Biomechanical Phenomena/statistics and numerical data"[Mesh]) OR ("Biomechanical Phenomena/trends"[Mesh]) OR (inflammatory) OR (external) OR (induce*) OR (associated) OR (complicat*) OR (mechanism) OR (pathophysiolog*) Or (physiology*) OR (biolog*) OR (adverse effect*) OR (disadvantage) or (side effect*) or (deterior* effect*) or (unpleasant effect)) |  |
|  | #4 | ((mechan* force*) OR (orthodont* force*) OR (force*) (pressure) OR (strain) OR (stress) OR (compressi*) OR (tension) OR (Biomechanics) OR (tensile)) |  |
|  | #5 | (("Root Resorption"[Mesh]) OR ("Tooth Resorption"[Mesh]) OR (root* resor?tion*) OR (root* crack*) OR (root* dissolution) OR (root* shortening) OR (root blunting) OR (root erosion) OR (tooth* resor?tion*) OR (tooth* crack*) OR (tooth* dissolution) OR (tooth* shortening) OR (tooth blunting) OR (tooth erosion) OR (teeth* resor?tion*) OR (teeth crack*) OR (teeth dissolution) OR (teeth shortening) OR (teeth blunting) OR (teeth erosion) OR (dent* resorption*) OR (dent* blunting) OR (dent* erosion) OR (dent* crack*) OR (dent* dissolution) OR (dent* shortening) OR (apex resorption*) OR (apex crack*) OR (apex dissolution) OR (apex shortening) OR (apex blunting) OR (apex erosion) OR (apical resor?tion*) OR (apical crack*) OR (apical dissolution) OR (apical shortening) OR (apical blunting) OR (apical erosion) OR (ortho* resor?tion*) OR (ortho* crack*) OR (ortho* dissolution) OR (ortho* shortening) OR (ortho* blunting) OR (ortho* erosion)) |  |
|  | #6 | ((in vitro[MeSH Terms]) OR (maxillary sinus[MeSH Terms]) OR (sinus[Title/Abstract]) OR (case reports[MeSH Terms]) OR (case study[Title/Abstract]) OR (case studies[Title/Abstract]) OR (case series[Title/Abstract]) OR (orthop*[Title/Abstract]) OR (zygomatic*[Title/Abstract]) OR (syndrome[Title/Abstract]) OR (osteonecrosis[Title/Abstract]) OR (osteoradionecrosis[Title/Abstract]) OR (osteoclastogenesis[Title/Abstract]) OR (bleaching[Title/Abstract]) OR (whiten*[Title/Abstract]) OR (traumat*[Title/Abstract]) OR (surg*[Title/Abstract]) OR (prothetic*[Title/Abstract]) OR (peridont* lesion[Title/Abstract]) Or (internal resorption[Title/Abstract]) OR (screw*[Title/Abstract]) OR (periimplant*[Title/Abstract]) OR (peri-implant*[Title/Abstract]) OR (reimplant[Title/Abstract]) OR (implant*[Title/Abstract]) OR (cleft lip[Title/Abstract]) OR (cleft palate[Title/Abstract]) OR (in vitro[Title/Abstract])) |  |
|  | #7 | #1 AND #2 AND #3 AND #4 AND#5 NOT #6 |  |

| **PICO**  **2b** | #1 | (("Animal Experimentation/adverse effects"[Mesh]) OR (animal experimentation[MeSH]) OR (animal model[MeSH]) OR (animal experiment [MeSH]) OR (animal trial[Title/Abstract]) OR (animal stud*[Title/Abstract]) OR ("Animal Experimentation/education"[Mesh]) OR ("Animal Experimentation/methods"[Mesh]) OR ("Animal Experimentation/pharmacology"[Mesh]) OR ("Animal Experimentation/standards"[Mesh]) OR ("Animal Experimentation/therapeutic use"[Mesh]) OR ("Animal Experimentation/veterinary"[Mesh]) OR ( "Animals/adverse effects"[Mesh]) OR ("Animals/classification"[Mesh]) OR ("Animals/complications"[Mesh]) OR ("Animals/diagnosis"[Mesh]) OR ("Animals/drug effects"[Mesh]) OR ("Animals/drug therapy"[Mesh]) OR ("Animals/genetics"[Mesh]) OR ("Animals/pathogenicity"[Mesh]) OR ("Animals/pharmacokinetics"[Mesh]) OR ("Animals/pharmacology"[Mesh]) OR ("Animals/physiopathology"[Mesh]) OR ("Animals/poisoning"[Mesh]) OR ("Animals/prevention and control"[Mesh]) OR ("Animals/therapeutic use"[Mesh]) OR ("Animals/therapy"[Mesh]) OR ("Animals/trends"[Mesh]) OR ("Animals/veterinary"[Mesh]) OR (dog[MeSH]) OR (mouse[Title/Abstract]) OR (mice[Title/Abstract]) OR (dog*[Title/Abstract]) OR (animal disease model[Title/Abstract])) | Language: English  Species Other Animals |
| --- | --- | --- | --- |
|  | #2 | (("Orthodontics/adverse effects"[Mesh]) OR ("Orthodontics/complications"[Mesh]) OR ("Orthodontics/diagnosis"[Mesh]) OR ("Orthodontics/methods"[Mesh]) OR ("Orthodontics/prevention and control"[Mesh]) OR ("Orthodontics/therapeutic use"[Mesh]) OR ("Orthodontics/therapy"[Mesh]) OR ("Orthodontics/trends"[Mesh]) OR ("Orthodontics, Corrective/adverse effects"[Mesh]) OR ("Orthodontics, Corrective/classification"[Mesh]) OR ("Orthodontics, Corrective/methods"[Mesh]) OR ("Orthodontics, Corrective/pharmacology"[Mesh]) OR ("Orthodontics, Corrective/prevention and control"[Mesh]) OR ("Orthodontics, Corrective/standards"[Mesh]) OR ("Orthodontics, Corrective/therapeutic use"[Mesh]) OR ("Orthodontics, Corrective/therapy"[Mesh]) OR ("Orthodontics, Corrective/trends"[Mesh]) OR ("Tooth Movement Techniques/adverse effects"[Mesh]) OR ("Tooth Movement Techniques/classification"[Mesh]) OR ("Tooth Movement Techniques/ethics"[Mesh]) OR ("Tooth Movement Techniques/instrumentation"[Mesh]) OR ("Tooth Movement Techniques/methods"[Mesh]) OR ("Tooth Movement Techniques/pharmacology"[Mesh]) OR ("Tooth Movement Techniques/prevention and control"[Mesh]) OR ("Tooth Movement Techniques/standards"[Mesh]) OR ("Tooth Movement Techniques/statistics and numerical data"[Mesh]) OR ("Tooth Movement Techniques/therapeutic use"[Mesh]) OR ("Tooth Movement Techniques/therapy"[Mesh]) OR ("Tooth Movement Techniques/trends"[Mesh]) OR (orthodon*) OR (periodont*) OR (tooth movement) OR (Teeth Movement*)) |  |
|  | #3 | (("Long Term Adverse Effects/chemically induced"[Mesh]) OR ("Long Term Adverse Effects/classification"[Mesh]) OR ("Long Term Adverse Effects/analysis"[Mesh]) OR ("Long Term Adverse Effects/diagnosis"[Mesh]) OR ("Long Term Adverse Effects/pathology"[Mesh]) OR ("Long Term Adverse Effects/physiology"[Mesh]) OR ("Long Term Adverse Effects/physiopathology"[Mesh]) OR ("Long Term Adverse Effects/prevention and control"[Mesh]) OR ("Long Term Adverse Effects/therapy"[Mesh]) OR ("adverse effects" [Subheading]) OR ("Biomechanical Phenomena/chemistry"[Mesh]) OR ("Biomechanical Phenomena/classification"[Mesh]) OR ("Biomechanical Phenomena/drug effects"[Mesh]) OR ("Biomechanical Phenomena/etiology"[Mesh]) OR ("Biomechanical Phenomena/genetics"[Mesh]) OR ("Biomechanical Phenomena/methods" [Mesh]) OR ("Biomechanical Phenomena/physiology"[Mesh]) OR ("Biomechanical Phenomena/standards"[Mesh]) OR ("Biomechanical Phenomena/statistics and numerical data"[Mesh]) OR ("Biomechanical Phenomena/trends"[Mesh]) OR (inflammatory) OR (external) OR (induce*) OR (associated) OR (complicat*) OR (mechanism) OR (pathophysiolog*) Or (physiology*) OR (biolog*) OR (adverse effect*) OR (disadvantage) or (side effect*) or (deterior* effect*) or (unpleasant effect)) |  |
|  | #4 | (("Risk Factors"[Mesh]) OR (risk factor*)) OR (risk agent*) OR (risk effect*) OR (risk emergent*) OR (risk reaction) OR (risk outcome*) OR (affecting factor*) OR (affecting agent*) OR (affecting effect*) OR (affecting event) OR (affecting emergent) OR (affecting reaction) OR (affecting outcome*) OR (inducing factors) OR (inducing agent) OR (inducing effect) OR (inducing emergent) OR (inducing reaction) OR (inducing outcome) OR (related factor) OR (related agent) OR (related effect*) OR (related event*) OR (related emergent*) OR (related reaction*) OR (related outcome*) OR (increasing factor*) OR (increasing agent*) OR (increasing effects) OR (increasing event*) OR (increasing emergent) OR (increasing reaction*) OR (increasing outcome*) OR (decreasing factor*) OR (decreasing agent*) OR (decreasing effect*) OR (decreasing event*) OR (decreasing emergent) OR (decreasing reaction*) OR (decreasing outcome*) OR (modif* factor*) OR (modif* agent*) OR (modif* effect*) OR (modif* event*) OR (modif* emergent) OR (modif* reaction*) OR (modif* outcome*) OR (sys* disease) OR (medication*) Or (supplement*) OR (drug*) OR (hormon*)) |  |
|  | #5 | (("Root Resorption"[Mesh]) OR ("Tooth Resorption"[Mesh]) OR (root* resor?tion*) OR (root* crack*) OR (root* dissolution) OR (root* shortening) OR (root blunting) OR (root erosion) OR (tooth* resor?tion*) OR (tooth* crack*) OR (tooth* dissolution) OR (tooth* shortening) OR (tooth blunting) OR (tooth erosion) OR (teeth* resor?tion*) OR (teeth crack*) OR (teeth dissolution) OR (teeth shortening) OR (teeth blunting) OR (teeth erosion) OR (dent* resorption*) OR (dent* blunting) OR (dent* erosion) OR (dent* crack*) OR (dent* dissolution) OR (dent* shortening) OR (apex resorption*) OR (apex crack*) OR (apex dissolution) OR (apex shortening) OR (apex blunting) OR (apex erosion) OR (apical resor?tion*) OR (apical crack*) OR (apical dissolution) OR (apical shortening) OR (apical blunting) OR (apical erosion) OR (ortho* resor?tion*) OR (ortho* crack*) OR (ortho* dissolution) OR (ortho* shortening) OR (ortho* blunting) OR (ortho* erosion)) |  |
|  | #6 | ((in vitro[MeSH Terms]) OR (maxillary sinus[MeSH Terms]) OR (sinus[Title/Abstract]) OR (case reports[MeSH Terms]) OR (case study[Title/Abstract]) OR (case studies[Title/Abstract]) OR (case series[Title/Abstract]) OR (orthop*[Title/Abstract]) OR (zygomatic*[Title/Abstract]) OR (syndrome[Title/Abstract]) OR (osteonecrosis[Title/Abstract]) OR (osteoradionecrosis[Title/Abstract]) OR (osteoclastogenesis[Title/Abstract]) OR (bleaching[Title/Abstract]) OR (whiten*[Title/Abstract]) OR (traumat*[Title/Abstract]) OR (surg*[Title/Abstract]) OR (prothetic*[Title/Abstract]) OR (peridont* lesion[Title/Abstract]) Or (internal resorption[Title/Abstract]) OR (screw*[Title/Abstract]) OR (periimplant*[Title/Abstract]) OR (peri-implant*[Title/Abstract]) OR (reimplant[Title/Abstract]) OR (implant*[Title/Abstract]) OR (cleft lip[Title/Abstract]) OR (cleft palate[Title/Abstract]) OR (in vitro[Title/Abstract])) |  |
|  | #7 | #1 AND #2 AND #3 AND #4 AND#5 NOT #6 |  |

| **PICO**  **3a** | #1 | (("Orthodontics/adverse effects"[Mesh]) OR ("Orthodontics/complications"[Mesh]) OR ("Orthodontics/diagnosis"[Mesh]) OR ("Orthodontics/methods"[Mesh]) OR ("Orthodontics/prevention and control"[Mesh]) OR ("Orthodontics/therapeutic use"[Mesh]) OR ("Orthodontics/therapy"[Mesh]) OR ("Orthodontics/trends"[Mesh]) OR ("Orthodontics, Corrective/adverse effects"[Mesh]) OR ("Orthodontics, Corrective/classification"[Mesh]) OR ("Orthodontics, Corrective/methods"[Mesh]) OR ("Orthodontics, Corrective/pharmacology"[Mesh]) OR ("Orthodontics, Corrective/prevention and control"[Mesh]) OR ("Orthodontics, Corrective/standards"[Mesh]) OR ("Orthodontics, Corrective/therapeutic use"[Mesh]) OR ("Orthodontics, Corrective/therapy"[Mesh]) OR ("Orthodontics, Corrective/trends"[Mesh]) OR ("Tooth Movement Techniques/adverse effects"[Mesh]) OR ("Tooth Movement Techniques/classification"[Mesh]) OR ("Tooth Movement Techniques/ethics"[Mesh]) OR ("Tooth Movement Techniques/instrumentation"[Mesh]) OR ("Tooth Movement Techniques/methods"[Mesh]) OR ("Tooth Movement Techniques/pharmacology"[Mesh]) OR ("Tooth Movement Techniques/prevention and control"[Mesh]) OR ("Tooth Movement Techniques/standards"[Mesh]) OR ("Tooth Movement Techniques/statistics and numerical data"[Mesh]) OR ("Tooth Movement Techniques/therapeutic use"[Mesh]) OR ("Tooth Movement Techniques/therapy"[Mesh]) OR ("Tooth Movement Techniques/trends"[Mesh]) OR (orthodon*) OR (periodont*) OR (tooth movement) OR (Teeth Movement*)) | Language: English  Medline  Species: Human |
| --- | --- | --- | --- |
|  | #2 | (("Long Term Adverse Effects/chemically induced"[Mesh]) OR ("Long Term Adverse Effects/classification"[Mesh]) OR ("Long Term Adverse Effects/analysis"[Mesh]) OR ("Long Term Adverse Effects/diagnosis"[Mesh]) OR ("Long Term Adverse Effects/pathology"[Mesh]) OR ("Long Term Adverse Effects/physiology"[Mesh]) OR ("Long Term Adverse Effects/physiopathology"[Mesh]) OR ("Long Term Adverse Effects/prevention and control"[Mesh]) OR ("Long Term Adverse Effects/therapy"[Mesh]) OR ("adverse effects" [Subheading]) OR ("Biomechanical Phenomena/chemistry"[Mesh]) OR ("Biomechanical Phenomena/classification"[Mesh]) OR ("Biomechanical Phenomena/drug effects"[Mesh]) OR ("Biomechanical Phenomena/etiology"[Mesh]) OR ("Biomechanical Phenomena/genetics"[Mesh]) OR ("Biomechanical Phenomena/methods" [Mesh]) OR ("Biomechanical Phenomena/physiology"[Mesh]) OR ("Biomechanical Phenomena/standards"[Mesh]) OR ("Biomechanical Phenomena/statistics and numerical data"[Mesh]) OR ("Biomechanical Phenomena/trends"[Mesh]) OR (inflammatory) OR (external) OR (induce*) OR (associated) OR (complicat*) OR (mechanism) OR (pathophysiolog*) Or (physiology*) OR (biolog*)) |  |
|  | #3 | ((mechan* force*) OR (orthodont* force*) OR (force*) (pressure) OR (strain) OR (stress) OR (compressi*) OR (tension) OR (Biomechanics) OR (tensile)) |  |
|  | #4 | (("Root Resorption"[Mesh]) OR ("Tooth Resorption"[Mesh]) OR (root* resor?tion*) OR (root* crack*) OR (root* dissolution) OR (root* shortening) OR (root blunting) OR (root erosion) OR (tooth* resor?tion*) OR (tooth* crack*) OR (tooth* dissolution) OR (tooth* shortening) OR (tooth blunting) OR (tooth erosion) OR (teeth* resor?tion*) OR (teeth crack*) OR (teeth dissolution) OR (teeth shortening) OR (teeth blunting) OR (teeth erosion) OR (dent* resorption*) OR (dent* blunting) OR (dent* erosion) OR (dent* crack*) OR (dent* dissolution) OR (dent* shortening) OR (apex resorption*) OR (apex crack*) OR (apex dissolution) OR (apex shortening) OR (apex blunting) OR (apex erosion) OR (apical resor?tion*) OR (apical crack*) OR (apical dissolution) OR (apical shortening) OR (apical blunting) OR (apical erosion) OR (ortho* resor?tion*) OR (ortho* crack*) OR (ortho* dissolution) OR (ortho* shortening) OR (ortho* blunting) OR (ortho* erosion)) |  |
|  | #5 | ((in vitro[MeSH Terms]) OR (maxillary sinus[MeSH Terms]) OR (sinus[Title/Abstract]) OR (animal experimentation[MeSH Terms]) OR (animal model[MeSH Terms]) OR (animal experiment[MeSH Terms]) OR (animal trial[Title/Abstract]) OR (animal stud*[Title/Abstract]) OR (case reports[MeSH Terms]) OR (case study[Title/Abstract]) OR (case studies[Title/Abstract]) OR (case series[Title/Abstract]) OR (dog[MeSH Terms]) OR (mouse[Title/Abstract]) OR (mice[Title/Abstract]) OR (dog*[Title/Abstract]) OR (animal disease model[Title/Abstract]) OR (photo*[Title/Abstract]) OR (orthop*[Title/Abstract]) OR (orthod*[Title/Abstract]) OR (zygomatic*[Title/Abstract]) OR (syndrome[Title/Abstract]) OR (osteoclastogenesis[Title/Abstract]) OR (osteonecrosis[Title/Abstract] OR (osteoradionecrosis[Title/Abstract]) OR (bleaching[Title/Abstract]) OR (traumat*[Title/Abstract]) OR (surg*[Title/Abstract]) OR (prothetic*[Title/Abstract]) OR (lesion[Title/Abstract]) Or (internal[Title/Abstract]) OR (screw*[Title/Abstract]) OR (periimplant*[Title/Abstract]) OR (peri-implant*[Title/Abstract]) OR (reimplant[Title/Abstract]) OR (implant*[Title/Abstract]) OR (cleft lip[Title/Abstract]) OR (cleft palate[Title/Abstract])) |  |
|  | #6 | #1 AND #2 AND #3 AND #4 NOT #5 |  |

| **PICO**  **3b** | #1 | (("Orthodontics/adverse effects"[Mesh]) OR ("Orthodontics/complications"[Mesh]) OR ("Orthodontics/diagnosis"[Mesh]) OR ("Orthodontics/methods"[Mesh]) OR ("Orthodontics/prevention and control"[Mesh]) OR ("Orthodontics/therapeutic use"[Mesh]) OR ("Orthodontics/therapy"[Mesh]) OR ("Orthodontics/trends"[Mesh]) OR ("Orthodontics, Corrective/adverse effects"[Mesh]) OR ("Orthodontics, Corrective/classification"[Mesh]) OR ("Orthodontics, Corrective/methods"[Mesh]) OR ("Orthodontics, Corrective/pharmacology"[Mesh]) OR ("Orthodontics, Corrective/prevention and control"[Mesh]) OR ("Orthodontics, Corrective/standards"[Mesh]) OR ("Orthodontics, Corrective/therapeutic use"[Mesh]) OR ("Orthodontics, Corrective/therapy"[Mesh]) OR ("Orthodontics, Corrective/trends"[Mesh]) OR ("Tooth Movement Techniques/adverse effects"[Mesh]) OR ("Tooth Movement Techniques/classification"[Mesh]) OR ("Tooth Movement Techniques/ethics"[Mesh]) OR ("Tooth Movement Techniques/instrumentation"[Mesh]) OR ("Tooth Movement Techniques/methods"[Mesh]) OR ("Tooth Movement Techniques/pharmacology"[Mesh]) OR ("Tooth Movement Techniques/prevention and control"[Mesh]) OR ("Tooth Movement Techniques/standards"[Mesh]) OR ("Tooth Movement Techniques/statistics and numerical data"[Mesh]) OR ("Tooth Movement Techniques/therapeutic use"[Mesh]) OR ("Tooth Movement Techniques/therapy"[Mesh]) OR ("Tooth Movement Techniques/trends"[Mesh]) OR (orthodon*) OR (periodont*) OR (tooth movement) OR (Teeth Movement*)) | Language: English  Species Other Animals |
| --- | --- | --- | --- |
|  | #2 | (("Long Term Adverse Effects/chemically induced"[Mesh]) OR ("Long Term Adverse Effects/classification"[Mesh]) OR ("Long Term Adverse Effects/analysis"[Mesh]) OR ("Long Term Adverse Effects/diagnosis"[Mesh]) OR ("Long Term Adverse Effects/pathology"[Mesh]) OR ("Long Term Adverse Effects/physiology"[Mesh]) OR ("Long Term Adverse Effects/physiopathology"[Mesh]) OR ("Long Term Adverse Effects/prevention and control"[Mesh]) OR ("Long Term Adverse Effects/therapy"[Mesh]) OR ("adverse effects" [Subheading]) OR ("Biomechanical Phenomena/chemistry"[Mesh]) OR ("Biomechanical Phenomena/classification"[Mesh]) OR ("Biomechanical Phenomena/drug effects"[Mesh]) OR ("Biomechanical Phenomena/etiology"[Mesh]) OR ("Biomechanical Phenomena/genetics"[Mesh]) OR ("Biomechanical Phenomena/methods" [Mesh]) OR ("Biomechanical Phenomena/physiology"[Mesh]) OR ("Biomechanical Phenomena/standards"[Mesh]) OR ("Biomechanical Phenomena/statistics and numerical data"[Mesh]) OR ("Biomechanical Phenomena/trends"[Mesh]) OR (inflammatory) OR (external) OR (induce*) OR (associated) OR (complicat*) OR (mechanism) OR (pathophysiolog*) Or (physiology*) OR (biolog*) OR (adverse effect*) OR (disadvantage) or (side effect*) or (deterior* effect*) or (unpleasant effect)) |  |
|  | #3 | (("Risk Factors"[Mesh]) OR (risk factor*)) OR (risk agent*) OR (risk effect*) OR (risk emergent*) OR (risk reaction) OR (risk outcome*) OR (affecting factor*) OR (affecting agent*) OR (affecting effect*) OR (affecting event) OR (affecting emergent) OR (affecting reaction) OR (affecting outcome*) OR (inducing factors) OR (inducing agent) OR (inducing effect) OR (inducing emergent) OR (inducing reaction) OR (inducing outcome) OR (related factor) OR (related agent) OR (related effect*) OR (related event*) OR (related emergent*) OR (related reaction*) OR (related outcome*) OR (increasing factor*) OR (increasing agent*) OR (increasing effects) OR (increasing event*) OR (increasing emergent) OR (increasing reaction*) OR (increasing outcome*) OR (decreasing factor*) OR (decreasing agent*) OR (decreasing effect*) OR (decreasing event*) OR (decreasing emergent) OR (decreasing reaction*) OR (decreasing outcome*) OR (modif* factor*) OR (modif* agent*) OR (modif* effect*) OR (modif* event*) OR (modif* emergent) OR (modif* reaction*) OR (modif* outcome*) OR (sys* disease) OR (medication*) Or (supplement*) OR (drug*) OR (hormon*)) |  |
|  | #4 | (("Root Resorption"[Mesh]) OR ("Tooth Resorption"[Mesh]) OR (root* resor?tion*) OR (root* crack*) OR (root* dissolution) OR (root* shortening) OR (root blunting) OR (root erosion) OR (tooth* resor?tion*) OR (tooth* crack*) OR (tooth* dissolution) OR (tooth* shortening) OR (tooth blunting) OR (tooth erosion) OR (teeth* resor?tion*) OR (teeth crack*) OR (teeth dissolution) OR (teeth shortening) OR (teeth blunting) OR (teeth erosion) OR (dent* resorption*) OR (dent* blunting) OR (dent* erosion) OR (dent* crack*) OR (dent* dissolution) OR (dent* shortening) OR (apex resorption*) OR (apex crack*) OR (apex dissolution) OR (apex shortening) OR (apex blunting) OR (apex erosion) OR (apical resor?tion*) OR (apical crack*) OR (apical dissolution) OR (apical shortening) OR (apical blunting) OR (apical erosion) OR (ortho* resor?tion*) OR (ortho* crack*) OR (ortho* dissolution) OR (ortho* shortening) OR (ortho* blunting) OR (ortho* erosion)) |  |
|  | #5 | ((in vitro[MeSH Terms]) OR (maxillary sinus[MeSH Terms]) OR (sinus[Title/Abstract]) OR (case reports[MeSH Terms]) OR (case study[Title/Abstract]) OR (case studies[Title/Abstract]) OR (case series[Title/Abstract]) OR (orthop*[Title/Abstract]) OR (zygomatic*[Title/Abstract]) OR (syndrome[Title/Abstract]) OR (osteonecrosis[Title/Abstract]) OR (osteoradionecrosis[Title/Abstract]) OR (osteoclastogenesis[Title/Abstract]) OR (bleaching[Title/Abstract]) OR (whiten*[Title/Abstract]) OR (traumat*[Title/Abstract]) OR (surg*[Title/Abstract]) OR (prothetic*[Title/Abstract]) OR (peridont* lesion[Title/Abstract]) Or (internal resorption[Title/Abstract]) OR (screw*[Title/Abstract]) OR (periimplant*[Title/Abstract]) OR (peri-implant*[Title/Abstract]) OR (reimplant[Title/Abstract]) OR (implant*[Title/Abstract]) OR (cleft lip[Title/Abstract]) OR (cleft palate[Title/Abstract]) OR (in vitro[Title/Abstract])) |  |
|  | #6 | #1 AND #2 AND #3 AND #4 NOT #5 |  |

**4. Scopus**

| **DataBase Search: Scopus** | | | | **Limits** |
| --- | --- | --- | --- | --- |
| PICO  1a |  | ( TITLE-ABS ( orthodon* ) OR TITLE-ABS ( "orthodontic device*" ) OR TITLE-ABS ( periodont* ) OR TITLE-ABS ( "tooth movement" ) OR TITLE-ABS ( "teeth movement" ) OR TITLE-ABS ( "root movement" ) OR TITLE-ABS ( "teeth biomencahnic" ) OR TITLE-ABS ( "root biomechanics" ) OR TITLE-ABS ( "dent* movement" ) OR TITLE-ABS ( "dent* biomechanic*" ) OR TITLE-ABS ( "periodont* movement" ) OR TITLE-ABS ( "apex movement" ) OR TITLE-ABS ( "apical movement" ) )  AND  ( TITLE-ABS ( *mechanic* AND force ) OR TITLE-ABS ( *mechanic* AND stress ) OR TITLE-ABS ( *mechanic* AND pressure ) OR TITLE-ABS ( *mechanic* AND strain ) OR TITLE-ABS ( *mechanic* AND compressi* ) OR TITLE-ABS ( *mechanic* AND tensi* ) OR TITLE-ABS ( *orthodont* AND force ) OR TITLE-ABS ( *orthodont* AND stress ) OR TITLE-ABS ( *orthodont* AND pressure ) OR TITLE-ABS ( *orthodont* AND strain ) OR TITLE-ABS ( *orthodont* AND compressi* ) OR TITLE-ABS ( orthodont* AND tensi* ) OR TITLE-ABS ( tooth AND force ) OR TITLE-ABS ( tooth AND stress ) OR TITLE-ABS ( tooth AND pressure ) OR TITLE-ABS ( tooth AND strain ) OR TITLE-ABS ( tooth AND compressi* ) OR TITLE-ABS ( tooth AND tensi* ) OR TITLE-ABS ( teeth AND force ) OR TITLE-ABS ( teeth AND stress ) OR TITLE-ABS ( teeth AND pressure ) OR TITLE-ABS ( teeth AND strain ) OR TITLE-ABS ( teeth AND compressi* ) OR TITLE-ABS ( teeth AND tensi* ) OR TITLE-ABS ( biolog* AND force ) OR TITLE-ABS ( biolog* AND stress ) OR TITLE-ABS ( biolog* AND pressure ) OR TITLE-ABS ( biolog* AND strain ) OR TITLE-ABS ( biolog* AND compressi* ) OR TITLE-ABS ( biolog* AND tensi* ) )  AND  ( TITLE-ABS ( root* AND resor?tion* ) OR TITLE-ABS ( root* AND crack* ) OR TITLE-ABS ( root* AND dissolution ) OR TITLE-ABS ( root* AND shortening ) OR TITLE-ABS ( "root blunting " ) OR TITLE-ABS ( "root erosion " ) OR TITLE-ABS ( tooth* AND resor?tion ) OR TITLE-ABS ( tooth* AND crack ) OR TITLE-ABS ( tooth* AND dissolution ) OR TITLE-ABS ( "tooth* shortening " ) OR TITLE-ABS ( "tooth blunting" ) OR TITLE-ABS ( teeth* AND resor?tion* ) OR TITLE-ABS ( teeth AND crack* ) OR TITLE-ABS ( teeth AND dissolution ) OR TITLE-ABS ( teeth AND shortening ) OR TITLE-ABS ( teeth AND blunting ) OR TITLE-ABS ( dent* AND resorption* ) OR TITLE-ABS ( dent* AND blunting ) OR TITLE-ABS ( dent* AND erosion ) OR TITLE-ABS ( dent* AND crack* ) OR TITLE-ABS ( dent* AND dissolution ) OR TITLE-ABS ( dent* AND shortening ) OR TITLE-ABS ( apex AND resorption* ) OR TITLE-ABS ( apex AND crack* ) OR TITLE-ABS ( apex AND dissolution ) OR TITLE-ABS ( apex AND shortening ) OR TITLE-ABS ( apex AND blunting ) OR TITLE-ABS ( apex AND erosion ) OR TITLE-ABS ( apical AND resor?tion* ) OR TITLE-ABS ( apical AND crack* ) OR TITLE-ABS ( apical AND dissolution ) OR TITLE-ABS ( apical AND shortening ) OR TITLE-ABS ( apical AND blunting ) OR TITLE-ABS ( apical AND erosion ) )  AND  (TITLE-ABS (“cell culture”) OR TITLE-ABS (“tissue culture”) OR TITLE-ABS (genetics) OR TITLE-ABS (biology) OR TITLE-ABS (biomarker) OR TITLE-ABS (molecular) OR TITLE-ABS (cell*) OR TITLE-ABS (laboratory) OR TITLE-ABS (“cell model”) OR TITLE-ABS (genotype) OR TITLE-ABS (RNA) OR TITLE-ABS (cell* count*) OR TITLE-ABS (gene expression) OR TITLE-ABS (cell scaffold) OR TITLE-ABS (“in vitro“) OR TITLE-ABS (preclinical) OR TITLE-ABS (subclinical) OR TITLE-ABS (tissue scaffold) OR TITLE-ABS(molecular genetics) OR TITLE-ABS(molecular biology) OR TITLE-ABS(biological marker) OR TITLE-ABS (tooth movement model*) OR TITLE-ABS (teeth movement model*) OR TITLE-ABS(simulat* tooth movement) OR TITLE-ABS(simulat* teeth movement) OR TITLE-ABS(culture model)  OR TITLE-ABS (tissue model *) OR TITLE-ABS (simulate* ortho* force) OR TITLE-ABS (cell research) OR TITLE-ABS (molecular research) OR TITLE-ABS (lab* research) OR TITLE-ABS (inflamm* markers))  AND NOT  ( TITLE-ABS (case stud*) OR TITLE-ABS (case report*) OR TITLE-ABS (case series) OR TITLE-ABS (zygomatic*) OR TITLE-ABS (syndrome) OR TITLE-ABS(bleaching) OR TITLE-ABS(screw*)  OR TITLE-ABS (periimplant*) OR TITLE-ABS (peri-implant*) OR TITLE-ABS (reimplant*) OR TITLE-ABS (implant*) OR TITLE-ABS (cleft lip) OR TITLE-ABS(cleft palate) OR TITLE-ABS (case reports) OR TITLE-ABS (experimental) OR TITLE-ABS (rat*) OR TITLE-ABS (rabbit*) OR TITLE-ABS ( "mouse" ) OR TITLE-ABS ( "mice" ) OR TITLE-ABS ( "dog*" ) OR TITLE-ABS(sheep) OR TITLE-ABS(veterinary) OR TITLE-ABS ( animal model*) OR TITLE-ABS ( "animal experiment*" ) OR TITLE-ABS ( "animal review" ) OR TITLE-ABS (preclinic* stud*) OR TITLE-ABS ( "animal trial") OR TITLE-ABS(animal stud*)) | | Subject area:  -All health science  Language:  -English |
| PICO  1b |  | ( TITLE-ABS ( orthodon* ) OR TITLE-ABS ( "orthodontic device*" ) OR TITLE-ABS ( periodont* ) OR TITLE-ABS ( "tooth movement" ) OR TITLE-ABS ( "teeth movement" ) OR TITLE-ABS ( "root movement" ) OR TITLE-ABS ( "teeth biomencahnic" ) OR TITLE-ABS ( "root biomechanics" ) OR TITLE-ABS ( "dent* movement" ) OR TITLE-ABS ( "dent* biomechanic*" ) OR TITLE-ABS ( "periodont* movement" ) OR TITLE-ABS ( "apex movement" ) OR TITLE-ABS ( "apical movement" ) ) AND ( TITLE-ABS ( risk AND factor* ) OR TITLE-ABS ( risk AND agent* ) OR TITLE-ABS ( risk AND effect* ) OR TITLE-ABS ( risk AND event* ) OR TITLE-ABS ( "risk emergent*" ) OR TITLE-ABS ( "risk reaction*" ) OR TITLE-ABS ( risk AND outcome* ) OR TITLE-ABS ( affecting AND factor* ) OR TITLE-ABS ( "Affecting agent*" ) OR TITLE-ABS ( "Affecting effect*" ) OR TITLE-ABS ( "Affecting event*" ) OR TITLE-ABS ( "Affecting emergent*" ) OR TITLE-ABS ( "Affecting reaction*" ) OR TITLE-ABS ( affecting AND outcome* ) OR TITLE-ABS ( inducing AND factor* ) OR TITLE-ABS ( "inducing agent*" ) OR TITLE-ABS ( "inducing effect*" ) OR TITLE-ABS ( "inducing event*" ) OR TITLE-ABS ( "inducing emergent*" ) OR TITLE-ABS ( "inducing reaction*" ) OR TITLE-ABS ( "inducing outcome*" ) OR TITLE-ABS ( related AND factor* ) OR TITLE-ABS ( related AND agent* ) OR TITLE-ABS ( related AND effect* ) OR TITLE-ABS ( "related event*" ) OR TITLE-ABS ( "related emergent*" ) OR TITLE-ABS ( "related reaction*" ) OR TITLE-ABS ( related AND outcome* ) OR TITLE-ABS ( increasing AND factor* ) OR TITLE-ABS ( "increasing agent*" ) OR TITLE-ABS ( increasing AND effect* ) OR TITLE-ABS ( "increasing event*" ) OR TITLE-ABS ( "increasing emergent" ) OR TITLE-ABS ( increasing AND reaction* ) OR TITLE-ABS ( increasing AND outcome* ) OR TITLE-ABS ( decreasing AND factor* ) OR TITLE-ABS ( "decreasing agent*" ) OR TITLE-ABS ( decreasing AND effect* ) OR TITLE-ABS ( "decreasing event*" ) OR TITLE-ABS ( "decreasing emergent" ) OR TITLE-ABS ( "decreasing reaction*" ) OR TITLE-ABS ( decreasing AND outcome* ) OR TITLE-ABS ( medication ) OR TITLE-ABS ( drug ) OR TITLE-ABS ( systemic AND disease* ) OR TITLE-ABS ( diabetes ) OR TITLE-ABS ( hormone* ) OR TITLE-ABS ( infection ) OR TITLE-ABS ( inflammation ) ) AND ( TITLE-ABS ( root* AND resor?tion* ) OR TITLE-ABS ( root* AND crack* ) OR TITLE-ABS ( root* AND dissolution ) OR TITLE-ABS ( root* AND shortening ) OR TITLE-ABS ( "root blunting " ) OR TITLE-ABS ( "root erosion " ) OR TITLE-ABS ( tooth* AND resor?tion ) OR TITLE-ABS ( tooth* AND crack ) OR TITLE-ABS ( tooth* AND dissolution ) OR TITLE-ABS ( "tooth* shortening " ) OR TITLE-ABS ( "tooth blunting" ) OR TITLE-ABS ( teeth* AND resor?tion* ) OR TITLE-ABS ( teeth AND crack* ) OR TITLE-ABS ( teeth AND dissolution ) OR TITLE-ABS ( teeth AND shortening ) OR TITLE-ABS ( teeth AND blunting ) OR TITLE-ABS ( dent* AND resorption* ) OR TITLE-ABS ( dent* AND blunting ) OR TITLE-ABS ( dent* AND erosion ) OR TITLE-ABS ( dent* AND crack* ) OR TITLE-ABS ( dent* AND dissolution ) OR TITLE-ABS ( dent* AND shortening ) OR TITLE-ABS ( apex AND resorption* ) OR TITLE-ABS ( apex AND crack* ) OR TITLE-ABS ( apex AND dissolution ) OR TITLE-ABS ( apex AND shortening ) OR TITLE-ABS ( apex AND blunting ) OR TITLE-ABS ( apex AND erosion ) OR TITLE-ABS ( apical AND resor?tion* ) OR TITLE-ABS ( apical AND crack* ) OR TITLE-ABS ( apical AND dissolution ) OR TITLE-ABS ( apical AND shortening ) OR TITLE-ABS ( apical AND blunting ) OR TITLE-ABS ( apical AND erosion ) ) AND ( TITLE-ABS ( "cell culture" ) OR TITLE-ABS ( "tissue culture" ) OR TITLE-ABS ( genetics ) OR TITLE-ABS ( biology ) OR TITLE-ABS ( biomarker ) OR TITLE-ABS ( molecular ) OR TITLE-ABS ( cell* ) OR TITLE-ABS ( laboratory ) OR TITLE-ABS ( "cell* model*" ) OR TITLE-ABS ( genotype ) OR TITLE-ABS ( rna ) OR TITLE-ABS ( cell* AND count* ) OR TITLE-ABS ( gene AND expression ) OR TITLE-ABS ( cell AND scaffold ) OR TITLE-ABS ( "in vitro" ) OR TITLE-ABS ( preclinical ) OR TITLE-ABS ( subclinical ) OR TITLE-ABS ( tissue AND scaffold ) OR TITLE-ABS ( molecular AND genetics ) OR TITLE-ABS ( molecular AND biology ) OR TITLE-ABS ( biological AND marker ) OR TITLE-ABS ( tooth AND movement AND model* ) OR TITLE-ABS ( teeth AND movement AND model* ) OR TITLE-ABS ( simulat* AND tooth AND movement ) OR TITLE-ABS ( simulat* AND teeth AND movement ) OR TITLE-ABS ( culture AND model ) OR TITLE-ABS ( tissue AND model* ) OR TITLE-ABS ( simulate* AND ortho* AND force ) OR TITLE-ABS ( cell AND research ) OR TITLE-ABS ( molecular AND research ) OR TITLE-ABS ( lab* AND research ) OR TITLE-ABS ( inflamm* AND markers ) ) AND NOT ( TITLE-ABS ( case AND stud* ) OR TITLE-ABS ( case AND report* ) OR TITLE-ABS ( case AND series ) OR TITLE-ABS ( zygomatic* ) OR TITLE-ABS ( syndrome ) OR TITLE-ABS ( bleaching ) OR TITLE-ABS ( screw* ) OR TITLE-ABS ( periimplant* ) OR TITLE-ABS ( peri-implant* ) OR TITLE-ABS ( reimplant* ) OR TITLE-ABS ( implant* ) OR TITLE-ABS ( cleft AND lip ) OR TITLE-ABS ( cleft AND palate ) OR TITLE-ABS ( experimental ) OR TITLE-ABS ( rat* ) OR TITLE-ABS ( rabbit* ) OR TITLE-ABS ( "mouse" ) OR TITLE-ABS ( "mice" ) OR TITLE-ABS ( "dog*" ) OR TITLE-ABS ( sheep ) OR TITLE-ABS ( veterinary ) OR TITLE-ABS ( animal AND model* ) OR TITLE-ABS ( "animal experiment*" ) OR TITLE-ABS ( "animal review" ) OR TITLE-ABS ( preclinic* AND stud* ) OR TITLE-ABS ( "animal trial" ) OR TITLE-ABS ( animal AND stud* ) ) | | Subject area:  -All health science  Language:  -English |
| PICO  2a |  | ( TITLE-ABS ( orthodon* ) OR TITLE-ABS ( "orthodontic device*" ) OR TITLE-ABS ( periodont* ) OR TITLE-ABS ( "tooth movement" ) OR TITLE-ABS ( "teeth movement" ) OR TITLE-ABS ( "root movement" ) OR TITLE-ABS ( "teeth biomencahnic" ) OR TITLE-ABS ( "root biomechanics" ) OR TITLE-ABS ( "dent* movement" ) OR TITLE-ABS ( "dent* biomechanic*" ) OR TITLE-ABS ( "periodont* movement" ) OR TITLE-ABS ( "apex movement" ) OR TITLE-ABS ( "apical movement" ) )  AND  ( TITLE-ABS ( *mechanic* AND force ) OR TITLE-ABS ( *mechanic* AND stress ) OR TITLE-ABS ( *mechanic* AND pressure ) OR TITLE-ABS ( *mechanic* AND strain ) OR TITLE-ABS ( *mechanic* AND compressi* ) OR TITLE-ABS ( *mechanic* AND tensi* ) OR TITLE-ABS ( *orthodont* AND force ) OR TITLE-ABS ( *orthodont* AND stress ) OR TITLE-ABS ( *orthodont* AND pressure ) OR TITLE-ABS ( *orthodont* AND strain ) OR TITLE-ABS ( *orthodont* AND compressi* ) OR TITLE-ABS ( orthodont* AND tensi* ) OR TITLE-ABS ( tooth AND force ) OR TITLE-ABS ( tooth AND stress ) OR TITLE-ABS ( tooth AND pressure ) OR TITLE-ABS ( tooth AND strain ) OR TITLE-ABS ( tooth AND compressi* ) OR TITLE-ABS ( tooth AND tensi* ) OR TITLE-ABS ( teeth AND force ) OR TITLE-ABS ( teeth AND stress ) OR TITLE-ABS ( teeth AND pressure ) OR TITLE-ABS ( teeth AND strain ) OR TITLE-ABS ( teeth AND compressi* ) OR TITLE-ABS ( teeth AND tensi* ) OR TITLE-ABS ( biolog* AND force ) OR TITLE-ABS ( biolog* AND stress ) OR TITLE-ABS ( biolog* AND pressure ) OR TITLE-ABS ( biolog* AND strain ) OR TITLE-ABS ( biolog* AND compressi* ) OR TITLE-ABS ( biolog* AND tensi* ) )  AND  ( TITLE-ABS ( root* AND resor?tion* ) OR TITLE-ABS ( root* AND crack* ) OR TITLE-ABS ( root* AND dissolution ) OR TITLE-ABS ( root* AND shortening ) OR TITLE-ABS ( "root blunting " ) OR TITLE-ABS ( "root erosion " ) OR TITLE-ABS ( tooth* AND resor?tion ) OR TITLE-ABS ( tooth* AND crack ) OR TITLE-ABS ( tooth* AND dissolution ) OR TITLE-ABS ( "tooth* shortening " ) OR TITLE-ABS ( "tooth blunting" ) OR TITLE-ABS ( teeth* AND resor?tion* ) OR TITLE-ABS ( teeth AND crack* ) OR TITLE-ABS ( teeth AND dissolution ) OR TITLE-ABS ( teeth AND shortening ) OR TITLE-ABS ( teeth AND blunting ) OR TITLE-ABS ( dent* AND resorption* ) OR TITLE-ABS ( dent* AND blunting ) OR TITLE-ABS ( dent* AND erosion ) OR TITLE-ABS ( dent* AND crack* ) OR TITLE-ABS ( dent* AND dissolution ) OR TITLE-ABS ( dent* AND shortening ) OR TITLE-ABS ( apex AND resorption* ) OR TITLE-ABS ( apex AND crack* ) OR TITLE-ABS ( apex AND dissolution ) OR TITLE-ABS ( apex AND shortening ) OR TITLE-ABS ( apex AND blunting ) OR TITLE-ABS ( apex AND erosion ) OR TITLE-ABS ( apical AND resor?tion* ) OR TITLE-ABS ( apical AND crack* ) OR TITLE-ABS ( apical AND dissolution ) OR TITLE-ABS ( apical AND shortening ) OR TITLE-ABS ( apical AND blunting ) OR TITLE-ABS ( apical AND erosion ) )  AND  ( TITLE-ABS (experimental) OR TITLE-ABS (rat*) OR TITLE-ABS (rabbit*) OR TITLE-ABS ( "mouse" ) OR TITLE-ABS ( "mice" ) OR TITLE-ABS ( "dog*" ) OR TITLE-ABS ( "canis canis" ) OR TITLE-ABS(sheep) OR TITLE-ABS(veterinary) OR TITLE-ABS ( animal model*) OR TITLE-ABS ( "animal experiment*" ) OR TITLE-ABS ( "animal review" ) OR TITLE-ABS (preclinic* stud*) OR TITLE-ABS ( "animal trial") OR TITLE-ABS(animal stud*) OR TITLE-ABS(nonhuman)) | | Subject area:  -All health science  Language:  -English |
| PICO  2b |  | | ( TITLE-ABS ( orthodon* ) OR TITLE-ABS ( "orthodontic device*" ) OR TITLE-ABS ( periodont* ) OR TITLE-ABS ( "tooth movement" ) OR TITLE-ABS ( "teeth movement" ) OR TITLE-ABS ( "root movement" ) OR TITLE-ABS ( "teeth biomencahnic" ) OR TITLE-ABS ( "root biomechanics" ) OR TITLE-ABS ( "dent* movement" ) OR TITLE-ABS ( "dent* biomechanic*" ) OR TITLE-ABS ( "periodont* movement" ) OR TITLE-ABS ( "apex movement" ) OR TITLE-ABS ( "apical movement" ) ) AND ( TITLE-ABS ( risk AND factor* ) OR TITLE-ABS ( risk AND agent* ) OR TITLE-ABS ( risk AND effect* ) OR TITLE-ABS ( risk AND event* ) OR TITLE-ABS ( "risk emergent*" ) OR TITLE-ABS ( "risk reaction*" ) OR TITLE-ABS ( risk AND outcome* ) OR TITLE-ABS ( affecting AND factor* ) OR TITLE-ABS ( "Affecting agent*" ) OR TITLE-ABS ( "Affecting effect*" ) OR TITLE-ABS ( "Affecting event*" ) OR TITLE-ABS ( "Affecting emergent*" ) OR TITLE-ABS ( "Affecting reaction*" ) OR TITLE-ABS ( affecting AND outcome* ) OR TITLE-ABS ( inducing AND factor* ) OR TITLE-ABS ( "inducing agent*" ) OR TITLE-ABS ( "inducing effect*" ) OR TITLE-ABS ( "inducing event*" ) OR TITLE-ABS ( "inducing emergent*" ) OR TITLE-ABS ( "inducing reaction*" ) OR TITLE-ABS ( "inducing outcome*" ) OR TITLE-ABS ( related AND factor* ) OR TITLE-ABS ( related AND agent* ) OR TITLE-ABS ( related AND effect* ) OR TITLE-ABS ( "related event*" ) OR TITLE-ABS ( "related emergent*" ) OR TITLE-ABS ( "related reaction*" ) OR TITLE-ABS ( related AND outcome* ) OR TITLE-ABS ( increasing AND factor* ) OR TITLE-ABS ( "increasing agent*" ) OR TITLE-ABS ( increasing AND effect* ) OR TITLE-ABS ( "increasing event*" ) OR TITLE-ABS ( "increasing emergent" ) OR TITLE-ABS ( increasing AND reaction* ) OR TITLE-ABS ( increasing AND outcome* ) OR TITLE-ABS ( decreasing AND factor* ) OR TITLE-ABS ( "decreasing agent*" ) OR TITLE-ABS ( decreasing AND effect* ) OR TITLE-ABS ( "decreasing event*" ) OR TITLE-ABS ( "decreasing emergent" ) OR TITLE-ABS ( "decreasing reaction*" ) OR TITLE-ABS ( decreasing AND outcome* ) OR TITLE-ABS ( medication ) OR TITLE-ABS ( drug ) OR TITLE-ABS ( systemic AND disease* ) OR TITLE-ABS ( diabetes ) OR TITLE-ABS ( hormone* ) OR TITLE-ABS ( infection ) OR TITLE-ABS ( inflammation ) ) AND ( TITLE-ABS ( root* AND resor?tion* ) OR TITLE-ABS ( root* AND crack* ) OR TITLE-ABS ( root* AND dissolution ) OR TITLE-ABS ( root* AND shortening ) OR TITLE-ABS ( "root blunting " ) OR TITLE-ABS ( "root erosion " ) OR TITLE-ABS ( tooth* AND resor?tion ) OR TITLE-ABS ( tooth* AND crack ) OR TITLE-ABS ( tooth* AND dissolution ) OR TITLE-ABS ( "tooth* shortening " ) OR TITLE-ABS ( "tooth blunting" ) OR TITLE-ABS ( teeth* AND resor?tion* ) OR TITLE-ABS ( teeth AND crack* ) OR TITLE-ABS ( teeth AND dissolution ) OR TITLE-ABS ( teeth AND shortening ) OR TITLE-ABS ( teeth AND blunting ) OR TITLE-ABS ( dent* AND resorption* ) OR TITLE-ABS ( dent* AND blunting ) OR TITLE-ABS ( dent* AND erosion ) OR TITLE-ABS ( dent* AND crack* ) OR TITLE-ABS ( dent* AND dissolution ) OR TITLE-ABS ( dent* AND shortening ) OR TITLE-ABS ( apex AND resorption* ) OR TITLE-ABS ( apex AND crack* ) OR TITLE-ABS ( apex AND dissolution ) OR TITLE-ABS ( apex AND shortening ) OR TITLE-ABS ( apex AND blunting ) OR TITLE-ABS ( apex AND erosion ) OR TITLE-ABS ( apical AND resor?tion* ) OR TITLE-ABS ( apical AND crack* ) OR TITLE-ABS ( apical AND dissolution ) OR TITLE-ABS ( apical AND shortening ) OR TITLE-ABS ( apical AND blunting ) OR TITLE-ABS ( apical AND erosion ) ) AND ( TITLE-ABS ( experimental ) OR TITLE-ABS ( rat* ) OR TITLE-ABS ( rabbit* ) OR TITLE-ABS ( "mouse" ) OR TITLE-ABS ( "mice" ) OR TITLE-ABS ( "dog*" ) OR TITLE-ABS ( sheep ) OR TITLE-ABS ( veterinary ) OR TITLE-ABS ( animal AND model* ) OR TITLE-ABS ( "animal experiment*" ) OR TITLE-ABS ( "animal review" ) OR TITLE-ABS ( preclinic* AND stud* ) OR TITLE-ABS ( "animal trial" ) OR TITLE-ABS ( animal AND stud* ) OR TITLE-ABS ( nonhuman ) ) AND NOT ( TITLE-ABS ( case AND stud* ) OR TITLE-ABS ( case AND report* ) OR TITLE-ABS ( case AND series ) OR TITLE-ABS ( zygomatic* ) OR TITLE-ABS ( syndrome ) OR TITLE-ABS ( bleaching ) OR TITLE-ABS ( screw* ) OR TITLE-ABS ( periimplant* ) OR TITLE-ABS ( peri-implant* ) OR TITLE-ABS ( reimplant* ) OR TITLE-ABS ( implant* ) OR TITLE-ABS ( cleft AND lip ) OR TITLE-ABS ( cleft AND palate ) OR TITLE-ABS ( case AND reports ) OR TITLE-ABS ( human ) OR TITLE-ABS ( patient ) OR TITLE-ABS ( participant* ) ) SUBJAREA ( medi OR nurs OR vete OR dent OR heal OR mult ) AND ( EXCLUDE ( SUBJAREA , "ENGI" ) ) AND ( EXCLUDE ( EXACTKEYWORD , "Human" ) OR EXCLUDE ( EXACTKEYWORD , "Humans" ) ) | Subject area:  -All health science  Language:  -English |
| PICO  3a |  | | ( TITLE-ABS ( orthodon* ) OR TITLE-ABS ( "orthodontic device*" ) OR TITLE-ABS ( periodont* ) OR TITLE-ABS ( "tooth movement" ) OR TITLE-ABS ( "teeth movement" ) OR TITLE-ABS ( "root movement" ) OR TITLE-ABS ( "teeth biomencahnic" ) OR TITLE-ABS ( "root biomechanics" ) OR TITLE-ABS ( "dent* movement" ) OR TITLE-ABS ( "dent* biomechanic*" ) OR TITLE-ABS ( "periodont* movement" ) OR TITLE-ABS ( "apex movement" ) OR TITLE-ABS ( "apical movement" ) )  AND  ( TITLE-ABS ( *mechanic* AND force ) OR TITLE-ABS ( *mechanic* AND stress ) OR TITLE-ABS ( *mechanic* AND pressure ) OR TITLE-ABS ( *mechanic* AND strain ) OR TITLE-ABS ( *mechanic* AND compressi* ) OR TITLE-ABS ( *mechanic* AND tensi* ) OR TITLE-ABS ( *orthodont* AND force ) OR TITLE-ABS ( *orthodont* AND stress ) OR TITLE-ABS ( *orthodont* AND pressure ) OR TITLE-ABS ( *orthodont* AND strain ) OR TITLE-ABS ( *orthodont* AND compressi* ) OR TITLE-ABS ( orthodont* AND tensi* ) OR TITLE-ABS ( tooth AND force ) OR TITLE-ABS ( tooth AND stress ) OR TITLE-ABS ( tooth AND pressure ) OR TITLE-ABS ( tooth AND strain ) OR TITLE-ABS ( tooth AND compressi* ) OR TITLE-ABS ( tooth AND tensi* ) OR TITLE-ABS ( teeth AND force ) OR TITLE-ABS ( teeth AND stress ) OR TITLE-ABS ( teeth AND pressure ) OR TITLE-ABS ( teeth AND strain ) OR TITLE-ABS ( teeth AND compressi* ) OR TITLE-ABS ( teeth AND tensi* ) OR TITLE-ABS ( biolog* AND force ) OR TITLE-ABS ( biolog* AND stress ) OR TITLE-ABS ( biolog* AND pressure ) OR TITLE-ABS ( biolog* AND strain ) OR TITLE-ABS ( biolog* AND compressi* ) OR TITLE-ABS ( biolog* AND tensi* ) )  AND  ( TITLE-ABS ( root* AND resor?tion* ) OR TITLE-ABS ( root* AND crack* ) OR TITLE-ABS ( root* AND dissolution ) OR TITLE-ABS ( root* AND shortening ) OR TITLE-ABS ( "root blunting " ) OR TITLE-ABS ( "root erosion " ) OR TITLE-ABS ( tooth* AND resor?tion ) OR TITLE-ABS ( tooth* AND crack ) OR TITLE-ABS ( tooth* AND dissolution ) OR TITLE-ABS ( "tooth* shortening " ) OR TITLE-ABS ( "tooth blunting" ) OR TITLE-ABS ( teeth* AND resor?tion* ) OR TITLE-ABS ( teeth AND crack* ) OR TITLE-ABS ( teeth AND dissolution ) OR TITLE-ABS ( teeth AND shortening ) OR TITLE-ABS ( teeth AND blunting ) OR TITLE-ABS ( dent* AND resorption* ) OR TITLE-ABS ( dent* AND blunting ) OR TITLE-ABS ( dent* AND erosion ) OR TITLE-ABS ( dent* AND crack* ) OR TITLE-ABS ( dent* AND dissolution ) OR TITLE-ABS ( dent* AND shortening ) OR TITLE-ABS ( apex AND resorption* ) OR TITLE-ABS ( apex AND crack* ) OR TITLE-ABS ( apex AND dissolution ) OR TITLE-ABS ( apex AND shortening ) OR TITLE-ABS ( apex AND blunting ) OR TITLE-ABS ( apex AND erosion ) OR TITLE-ABS ( apical AND resor?tion* ) OR TITLE-ABS ( apical AND crack* ) OR TITLE-ABS ( apical AND dissolution ) OR TITLE-ABS ( apical AND shortening ) OR TITLE-ABS ( apical AND blunting ) OR TITLE-ABS ( apical AND erosion ) )  AND  (TITLE-ABS (patient*) OR TITLE-ABS (individual*) OR TITLE-ABS (human) OR TITLE-ABS (clinical) OR TITLE-ABS (subjects) OR TITLE-ABS (participant*) OR TITLE-ABS(men) OR TITLE-ABS(women) OR TITLE-ABS(male) OR TITLE-ABS(female) OR TITLE-ABS(volunteer)) | Subject area:  -All health science  Language:  -English |
| PICO  3b |  | | ( TITLE-ABS ( orthodon* ) OR TITLE-ABS ( "orthodontic device*" ) OR TITLE-ABS ( periodont* ) OR TITLE-ABS ( "tooth movement" ) OR TITLE-ABS ( "teeth movement" ) OR TITLE-ABS ( "root movement" ) OR TITLE-ABS ( "teeth biomencahnic" ) OR TITLE-ABS ( "root biomechanics" ) OR TITLE-ABS ( "dent* movement" ) OR TITLE-ABS ( "dent* biomechanic*" ) OR TITLE-ABS ( "periodont* movement" ) OR TITLE-ABS ( "apex movement" ) OR TITLE-ABS ( "apical movement" ))  AND  (TITLE-ABS (patient*) OR TITLE-ABS (individual*) OR TITLE-ABS (human) OR TITLE-ABS (clinical) OR TITLE-ABS (subjects) OR TITLE-ABS (participant*) OR TITLE-ABS(men) OR TITLE-ABS(women) OR TITLE-ABS(male) OR TITLE-ABS(female) OR TITLE-ABS(volunteer))  AND  ( TITLE-ABS ( root* AND resor?tion* ) OR TITLE-ABS ( root* AND crack* ) OR TITLE-ABS ( root* AND dissolution ) OR TITLE-ABS ( root* AND shortening ) OR TITLE-ABS ( "root blunting " ) OR TITLE-ABS ( "root erosion " ) OR TITLE-ABS ( tooth* AND resor?tion ) OR TITLE-ABS ( tooth* AND crack ) OR TITLE-ABS ( tooth* AND dissolution ) OR TITLE-ABS ( "tooth* shortening " ) OR TITLE-ABS ( "tooth blunting" ) OR TITLE-ABS ( teeth* AND resor?tion* ) OR TITLE-ABS ( teeth AND crack* ) OR TITLE-ABS ( teeth AND dissolution ) OR TITLE-ABS ( teeth AND shortening ) OR TITLE-ABS ( teeth AND blunting ) OR TITLE-ABS ( dent* AND resorption* ) OR TITLE-ABS ( dent* AND blunting ) OR TITLE-ABS ( dent* AND erosion ) OR TITLE-ABS ( dent* AND crack* ) OR TITLE-ABS ( dent* AND dissolution ) OR TITLE-ABS ( dent* AND shortening ) OR TITLE-ABS ( apex AND resorption* ) OR TITLE-ABS ( apex AND crack* ) OR TITLE-ABS ( apex AND dissolution ) OR TITLE-ABS ( apex AND shortening ) OR TITLE-ABS ( apex AND blunting ) OR TITLE-ABS ( apex AND erosion ) OR TITLE-ABS ( apical AND resor?tion* ) OR TITLE-ABS ( apical AND crack* ) OR TITLE-ABS ( apical AND dissolution ) OR TITLE-ABS ( apical AND shortening ) OR TITLE-ABS ( apical AND blunting ) OR TITLE-ABS ( apical AND erosion ) )  AND  (TITLE-ABS ( risk factor*) OR TITLE-ABS (risk agent*) OR TITLE-ABS (risk effect*) OR TITLE-ABS (risk event*) OR TITLE-ABS ("risk emergent*") OR TITLE-ABS ("risk reaction*") OR TITLE-ABS (risk outcome*) OR TITLE-ABS (Affecting factor*) OR TITLE-ABS ("Affecting agent*") OR TITLE-ABS ("Affecting effect*") OR TITLE-ABS ("Affecting event*") OR TITLE-ABS ("Affecting emergent*") OR TITLE-ABS ("Affecting reaction*") OR TITLE-ABS (Affecting outcome*) OR TITLE-ABS (inducing factor*) OR TITLE-ABS ("inducing agent*") OR TITLE-ABS ("inducing effect*") OR TITLE-ABS( "inducing event*") OR TITLE-ABS ("inducing emergent*") OR TITLE-ABS ("inducing reaction*") OR TITLE-ABS ("inducing outcome*") OR TITLE-ABS (related factor*) OR TITLE-ABS (related agent*) OR TITLE-ABS (related effect*) OR TITLE-ABS ("related event*") OR TITLE-ABS ("related emergent*") OR TITLE-ABS ("related reaction*") OR TITLE-ABS (related outcome*) OR TITLE-ABS (increasing factor*) OR TITLE-ABS ("increasing agent*") OR TITLE-ABS (increasing effect*) OR TITLE-ABS ("increasing event*") OR TITLE-ABS ("increasing emergent") OR TITLE-ABS (increasing reaction*) OR TITLE-ABS (increasing outcome*) OR TITLE-ABS (decreasing factor*) OR TITLE-ABS ("decreasing agent*") OR TITLE-ABS (decreasing effect*) OR TITLE-ABS ("decreasing event*") OR TITLE-ABS ("decreasing emergent") OR TITLE-ABS ("decreasing reaction*”) OR TITLE-ABS (decreasing outcome*) OR TITLE-ABS (medication) OR TITLE-ABS (drug) OR TITLE-ABS (systemic disease*) OR TITLE-ABS (diabetes) OR TITLE-ABS (hormone*) OR TITLE-ABS (infection) OR TITLE-ABS (inflammation))  AND NOT  ( TITLE-ABS (case stud*) OR TITLE-ABS (case report*) OR TITLE-ABS (case series) OR TITLE-ABS (zygomatic*) OR TITLE-ABS (syndrome) OR TITLE-ABS(bleaching) OR TITLE-ABS(screw*)  OR TITLE-ABS (periimplant*) OR TITLE-ABS (peri-implant*) OR TITLE-ABS (reimplant*) OR TITLE-ABS (implant*) OR TITLE-ABS (cleft lip) OR TITLE-ABS(cleft palate) OR TITLE-ABS (case reports) OR TITLE-ABS (experimental) OR TITLE-ABS (rat*) OR TITLE-ABS (rabbit*) OR TITLE-ABS ( "mouse" ) OR TITLE-ABS ( "mice" ) OR TITLE-ABS ( "dog*" ) OR TITLE-ABS(sheep) OR TITLE-ABS(veterinary) OR TITLE-ABS ( animal model*) OR TITLE-ABS ( "animal experiment*" ) OR TITLE-ABS ( "animal review" ) OR TITLE-ABS (preclinic* stud*) OR TITLE-ABS ( "animal trial") OR TITLE-ABS(animal stud*)) | Subject area:  -All health science  Language:  -English |
