# Supplementary material for: Under pressure—mechanisms and risk factors for orthodontically induced inflammatory root resorption: a systematic review
Source: Eur J Orthod. 2023 Jun 27;45(5):612–26. doi: 10.1093/ejo/cjad011 (PMC10505745; doi:10.1093/ejo/cjad011)
Supplement: cjad011_suppl_Supplementary_Table_S4 [file cjad011_suppl_supplementary_table_s4.docx]

| **Supplementary Table 4: PICO 1A, Studies' Summary** | | | | | | | | |  |
| --- | --- | --- | --- | --- | --- | --- | --- | --- | --- |
| **Authors** | **Design** | **Age**  **Mean (Range) or mean ± SD in years** | **Gender Distribution (% females)** | | **Groups** | **Force Protocol** | **Loading time in hours** | **Results (Sig=Significant Difference)** |  |
| Kang et al., 2013 | 4 Gps / 2D and 3D cultures / | 24 (21, 24, 27) | 100 | | Test | CSC/2 g/cm2 | 2, 48 | Sig test Gp compared to control Gp ( fold change ≥ 2, P < 0.05): 2 Fold up: 2D Gp: 127 3D Gp: 379; 0.5 fold down: 2D Gp: 64 3D Gp: 154 Total: 2D Gp: 191 genes 3D Gp:533 genes |  |
|  |  |  |  |  | Control | No force |  |  |  |
| Pinkerton et al., 2008 | 2 Gps / 1 type of force / 2 periods | NI | NI | | Test | ISCY (6s/90s) / 12% deformation | 6 | T1: Sig test Gp compared to control Gp (fold change ≥ 2): UpRe genes 20; DoRe genes: 21  Sig p < 0.05; UpRe genes: 3 ; DoRe genes: 3 |  |
|  |  |  |  |  |  |  | 12 | T2: Sig test Gp compared to control Gp (fold change ≥ 2): UpRe genes: 20; DoRe genes: 16 Sig p < 0.05: UpRe genes: 4; DoRe genes: 1 |  |
|  |  |  |  |  | Control | No force | 24 | T3: Sig test Gp compared to control Gp (fold change ≥ 2): UpRe genes: 24; DoRe genes: 17 Sig p < 0.05: UpRe genes: 7; DoRe genes: 0 |  |
| Chen et al., 2015 | 3 Gps/ 2 types of force / 1 period | (18 - 25) | Ni | | Test 1 | ISCY (6cycles/min) /12% deformation | 24 | T1: Sig test Gp compared to control Gp (P < 0.05): decreased expression of miR-29 family member miRs about 0.5-fold, UpRe major ECM genes |  |
|  |  |  |  |  | Test 2 | CSC force / 2g/cm2 |  | T2: Sig test Gp compared to control Gp (P < 0.05) increased expression of miR-29 family (1.8–4 folds) fold, UpRe major ECM genes |  |
|  |  |  |  |  | Control | No force |  |  |  |
| Lee et al., 2007 | 2 Gps / 3D culture/ 1 type of force / 2 periods | NI | NI | | Test | CSC force / 2g/cm2 | 2 | T1: Sig test Gp compared to control Gp (fold change ≥ 1.63): UpRe genes= 28; DoRe genes= 10 Among these UpRe genes, ALP and IL-6 (potent osteoclast activator) |  |
|  |  |  |  |  | Test 2 | CSC force / 2g/cm2 | 12 | T2: Sig test Gp compared to control Gp (fold change ≥ 1.63): UpRe genes= 8; DoRe genes= 30 Among these UpRe genes, ALP and IL-6 (potent osteoclast activator) |  |
|  |  |  |  |  | Control | No force | 2,12 |  |  |
| Li et al., 2019 | 3 Gps / 1 type of force /1 period | NI | NI | | Test | CSC force / 1g/cm^2^ | 24 | Sig test Gp compared to control Gp (P < 0.05): 41 factors of forkhead family, Only FOXM1 show Sig changes; Inhibition of FOXM1 in compressed PDLCs Promotes RANKL/OPG ratio and Osteoclast Differentiation |  |
|  |  |  |  |  | Control | No force |  |  |  |
| Wu et al., 2019 | 2 Gps/ 1 type of force / 1 period | 14-25 | NI | | Test | ISCY (60 cycles/min) force / 10% equibiaxial strain | 24 | Sig test Gp Compared to control Gp (P< 0.05 and A fold change ≥ 2): A Sig higher expression level of osteogenic/cementoblastic genes and proteins Sig UpRe: 31 miRNAs; Sig DoRe: 16 miRNAs Sig Enriched pathways of the target genes: 20, Among them, the MAPK signalling pathway, cAMP signalling pathway, and Hippo signalling pathway (regulation of osteogenesis). |  |
|  |  |  |  |  | Control | No force |  |  |  |
| De Araujo et al., 2007 | 2 Gps / 3D culture / 6 periods | NI | NI | | Test | CSC/3.6,6.0,7.2, 9.5g/cm2 | 6 | Sig test Gp compared to control Gp (Sig P< 0.05 and fold change ≥ 2) : Sig 108 of 30,000 genes tested, UpRe: 85 genes DoRe: 23 genes |  |
|  |  |  |  |  | Test | CSC force / 6.0 g/cm2 | 1,3,6,13,24,72 |  |  |
|  |  |  |  |  | Control | No force |  |  |  |
| Wei et al., 2014 | 2 Gps / 1 force / 1 period | 12-16 | NI | | Test | ISCY (60 cycles/min)/ 10% equibiaxial strain | 12 | A total of 748 miRNA was expressed in stretch Sig Compared to control (P < 0.01): 53 miRNAs; UpRe: 26; DoRe: 27 |  |
|  |  |  |  |  | Control | No force |  | A total of 841 miRNAs was expressed in normal |  |
| Fleissig et al., 2018 | 4 Gps/ 1 force type/ 1 period | 12-30 | 0 | | Test | HyCP / 100 g/cm2 | 24 | Sig test compared to control (P < 0.05 and fold change ≥ 2):  UpRe: 68 genes (out of 131) DoRe: 19 genes (out of 41) |  |
|  |  |  |  |  | Control | No force |  |  |  |
| Ma et al., 2015 | 4 Gps /1 force type/ 2 periods | 11 | 100 | | Test | ISCY (6 cycles/min) / 10% stretch strain | 6 | T1: Sig test compared to control (P < 0.05 and fold change ≥ 1.5):UpRe: 6 genes; DoRe: 3 genes |  |
|  |  |  |  |  | Control | No force | 24 | T2: Sig test compared to control (P < 0.05 and fold change ≥ 2): UpRe: 12 genes; DoRe: 2 genes |  |
| Saminathan et al, 2012 | 4 Gps / 1 force type / 3 periods | NI | NI | | Test | ICY tensile strain (5s cycle/1.5min) / 12% | 6 | T1: Sig test compared to control (P < 0.05): Sig in 16 mRNAs genes; UpRe:21 (1 Sig); DoRe: 52 (4 Sig) |  |
|  |  |  |  |  | Control | No force | 12 | T2: UpRe: 24 (Sig 2); DoRe: 49 (Sig 5) |  |
|  |  |  |  |  |  |  | 24 | T3: UpRe: 29 (Sig 2); DoRe: 43 (Sig 3) |  |
| Memmert et al.,2020 | 5 Gps / 3 force type / 2 period | 15 (11-19) | NI | | Test | CST / 3% | 1 | T1: NI |  |
|  |  |  |  |  | Test | CST / 20% | 24 | T2: Sig test compared to control (P < 0.05 and fold change ≥ 1.5): UpRe 9 ; DoRe 3 Ingenuity Pathway Analysis (IPA) revealed a complex gene network. |  |
|  |  |  |  |  | Control | No force |  |  |  |
| Manokawinchoke et al., 2019 | 4 Gps / 2typs of force / 1 period | 18 ‒ 35 | NI | | Test | CSC, /1.5 g/cm2 | 24 | T1: Sig test compared to control (P < 0.05 and fold change ≥ 2): Overall 482 among these DoRe genes calcium signalling pathway and UpRe genes are ECM-receptor interaction, focal adhesion, and TGF-β signalling pathway |  |
|  |  |  |  |  | Test | ICCY (13.8 cycle/min)/1.5 g/cm2 |  | T2: Sig test compared to control (P < 0.05 and fold change ≥ 2): Overall 2290 among these down-regulated genes, calcium signalling pathway and UpRe genes are ECM-receptor interaction, focal adhesion, and TGF-β signalling pathway |  |
|  |  |  |  |  | Control | No force |  |  |  |
| Yamashiro et al., 2007 | 2 Gps / 1 type of force / 3 periods | 21 (17, 21, 22, 24) | 75 | | Test | ISCY (6 cycles/min) / 18% (36 kPa) | 0.5 | T1: Sig test compared to control ( >2-fold, P < 0.05):Sig changes of mRNA accumulation for 122 CT genes down-regulated all nuclear transcription factors except v-fos FBJ murine osteosarcoma viral oncogene homolog (FOS) in 0.5h: UpRe: 50 genes; DoRe: 25 genes |  |
|  |  |  |  |  |  |  | 1 | T2: Sig test compared to control ( >2-fold, P < 0.05): UpRe: 70 genes; DoRe: 52 genes |  |
|  |  |  |  |  | Control | No force | 2 | T3: Sig test compared to control ( >2-fold, P < 0.05): UpRe: 46 genes; DoRe: 29 genes |  |
|  |  |  |  |  |  |  | 16 | T4: Sig test compared to control ( >2-fold, P < 0.05): UpRe: 23 genes; DoRe: 68 genes |  |
| Wu et al., 2010 | 2 Gps/ 1 force / 1 period | 12-14 | NI | | Test | ICCY(30c/min)/4kµstr | 2 | Sig test compared to control (>2-fold, P < 0.01): 217 Sig expressed; UpRe: 207; DoRe: 10 10 signalling pathways associated with genes differentially expressed ( p<0.01) |  |
|  |  |  |  |  | Control | No force |  |  |  |
| Liu et al., 2011 | 2 Gps / 1 type of force / 1 period | 12-15 | NI | | Test | ISCY (6 cycles/min) / 12% deformation | 24 | Overall expression of the 96 genes Sig test compared to control (>2-fold): 23 genes; UpRe: 21 genes, DoRe: 2 genes |  |
|  |  |  |  |  | Control | No force |  |  |  |
| Chang et al., 2014 | 2 Gps / 1 type of force / 1 period | NI | NI | | Test | ISCY (6 cycles/min) / 12% deformation | 72 | Sig test compared to control ( fold change > 2, P < 0.05): UpRe: 17 miRNAs, DoRe: 15 miRNAs mRNA: Overall, 818 screened mRNAs  Sig test compared to control(≥3.5 fold change or P ≤0.05): UpRe: 818 mRNAs,DoRe:344 mRNAs |  |
|  |  |  |  |  | Control | No force |  |  |  |
| Huang et al., 2015 | 2 Gps / 1 type of force / 1 period | 15 (12-18) | 66.67 | | Test | CSC force / 2g/cm2 | 12 | Control Gp: 4029 lncRNAs, 10 231 mRNAs; Test Gp: 4065 lncRNAs, 10 254 mRNAs Sig (>1.5fold or<0.6667 or FDR<0.05):90 of the lncRNAs Sig:UpRe:72 lncRNAs,DoRe:18 lncRNAs Differentially expressed 519 mRNAs: UpRe: 373 mRNAs, DoRe: 146 mRNAs 30 pathways were Sig affected in terms of differentially expressed genes |  |
|  |  |  |  |  | Control | No force |  |  |  |
| Wang et al., 2020 | 2 Gps / 1 type of force / 1 period | 22-25 | NI | | Test | ISCY (60 cycles/min) / 10% equibiaxial strain | 12 | Stretch Gp: Detection of 11,810 unique lncRNAs in a stretch, 6,096 novel lncRNAs with 3,664 intergenic, 61 sense, 307 intronic, and 2,064 antisense lncRNAs  Non-stretched: Detection of 11,531 unique lncRNAs, 5,649 lncRNAs were novel, including 3,385 intergenic, 55 sense, 316 intronic, and 1,893 antisense lncRNAs  Sig and fold > 2: 1,339 lncRNAs, UpRe 799, DoRe 540; mRNAs: 1,426, UpRe 757, DoRe 669 |  |
|  |  |  |  |  | Control | No force |  |  |  |
| Lin et al., 2021 | 2 Gp/1type of force/1 period | 18-33 | 66.67 | | Test | CST force/12% strain | 12, 72 | Tension Gp relative to control Gp: mRNAs, UpRe: 860, DoRe 88, lncRNAs, UpRe:107, DoRe 88 |  |
|  |  |  |  |  | Control | No force |  |  |  |
| Liu et al., 2021 | 2 Gp / 1 type of force/ 1 period | 38.9±7 | NI | Test PPDLs | | ISCY (6 cycles/min) / 12% elongation | 12 | Sig lncRNAs HPDLs:8,847;1,624 of them only in HPDLs;PPDLs:9,772; 2,549 only in PPDLs Sig mRNAs HPDLs:11,937;2,170 of them only in HPDLs; PPDLs:12,410; 2,643 only in PPDLs |  |
|  |  | 37.9±7 |  | Test HPDL | |  |  |  |  |
|  |  |  |  | Control | | No force |  |  |  |
