# Supplementary material for: Under pressure—mechanisms and risk factors for orthodontically induced inflammatory root resorption: a systematic review
Source: Eur J Orthod. 2023 Jun 27;45(5):612–26. doi: 10.1093/ejo/cjad011 (PMC10505745; doi:10.1093/ejo/cjad011)
Supplement: cjad011_suppl_Supplementary_Table_S9 [file cjad011_suppl_supplementary_table_s9.docx]

| **Supplementary Table 9: PICO 2A, Studies’ Summary** | | | | | | | |
| --- | --- | --- | --- | --- | --- | --- | --- |
| **Author** | **Groups** | **Animals counts (n)** | **Age at the start (Mean ± SD (range)) m=months**  **W= weeks** | **Type of orthodontic device** | **Amount of force** | **Follow-up loading period in days** | **Main outcomes in**  **(Mean ± SD (range); or Number (%))** **, (Sig=Significant Difference)**  **Length in μm; area in μm^2^ , Volume in μm^3^** |
| Aghili et al., 2015 | Test | 15 | 3m | Close coil spring | 50g | 14 | RR (mean length of the root periphery to the sum of the length of resorptive lacunae) |
|  | Control | 15 |  |  | 50g | 14 |  |
|  | Control | 15 CL side |  |  | 0g | 14 |  |
| Aghili et al., 2013 | Test | 15 | 3m | Close coil spring | 50g | 14 | No Sig in RR area 24.4 ± 8.5 |
|  | Control | 15 |  |  | 0g | 14 | 17.2 ± 16.6 |
| Asefi et al., 2018 | Test | 10 | 6m | Close coil spring | 30g | 21 | No of RR lacunae: 4±0.055; Area of RR lacunae: 0.270×10^−3^±0.013 |
|  | Control | 10 |  |  | 0g | 21 | Number of RR lacunae: 0±0.002; Area of RR lacunae: 0.003×10^−3^±0.000 |
| Brunet et al., 2016 | Test | 30 then 10 in 14d | 9w | Close coil spring | 30.59g(30cN) | 14 | No of animals (% of RR): 10 (80) |
|  | Control | 30 then 10 in 14d |  |  | 0g | 14 | No of animals (% of RR): 10 (0) |
| Chung et al., 2008 | Test | 11 | 48 ± 3.6w | Close coil spring | 10g | 21 | Sig more Change in root thickness % (plot digitizer): Gp1: 52 ± 4; Gp2: 68 ± 3 |
|  | Control | 11 |  |  | 0g | 21 | Gp3: 80 ± 3; Gp4: 76 ± 3 |
| King et al., 1997 | Test | 72 | NI | Close coil spring | 40gm | 16 | Sig: RR in test Gp is 10.4 times that of control Gp. |
|  | Control | 72 |  |  | No force | 16 |  |
| Konoo et al., 2001 | Test | Intermittent Gp=48 | 10-11w | Close coil spring | 40g/1h/day | 14 | Sig RR in continuous force after 14d |
|  | Test | Continuous Gp=48 |  |  | 40g/24h/day | 14 |  |
|  | Control | Control Gp=48 |  |  | 0g | 14 |  |
| Miyoshi et al., 2001 | Test | 30 (10 in each subGp) | 6w | Expansion spring | 16.8g (165mN) | 21 | Mean RR area: CGp 2.958 X 10^4^ LGp 2.499 X 10^4^ DGp 1.816 X 10^4^ Sig RR in the whole day Gp, then light period Gp then dark period lastly the control Gp |
|  | Control | 10 |  |  | 0g | 21 |  |
| Verna et al., 2003 | Test | Gp1:19; Gp2:16; Gp3:17 | 6m | Close coil spring | 25g | 21 | Root length: Gp1 2495 (423); Gp2 2556 (227); Gp3 2451 (213) |
|  | Control | Gp1:19; Gp2:16; Gp3:17 |  |  | 0g | 21 | Root length: Gp1 2532 (248); Gp2 2527 (189); Gp3 2386 (172) |
| Yeoh et al., 2017 | Test | 11 | 10w | Close coil spring | 100g | 14 | Mean (SE) mm3 30 Hz non-loaded & 30 Hz loaded 17.584 (5.343) 60 Hz non-loaded & 60 Hz loaded 16.524 (4.786) Control non-loaded & positive control 28.4330(6.7867) |
|  | Control | 11 |  |  | 0g | 14 |  |
| Gul Amuk et al.,2020 | Test | 20 | 12w | Helical expansion spring | 50g | 14 | Sig RR volume bw Gps B-RR. vol. 0.016 ± 0.004; MB-RR. vol. 0.047 ± 0.021 |
|  | Control | 20 |  |  | 0g | 14 | B-RR. vol. 0.001 ± 0.000; MB-RR. vol. 0.017 ± 0.005 |
| Kameyama et al., 2003 | Test | Continuous force  Gp: 12; 1h Gp: 12 4h Gp: 12; 9h Gp: 12 | 3m | Close coil spring | 10g | 14 | Sig RR area bw Gps 0h =8x10^3^; 1h =7x10^3^ 4h = 0.8x10^3^; 9h =0.5x10^3^ |
|  | Control | 12 |  |  | 0g | 14 | No Sig in RR area between control Gp |
| Tyrovola et al.,2010 | Test | 14 | 10w | Close coil spring | 25g | 21 | Sig more RR area = 37233.18 ± 9848.12; Ratio of RR = 0.0488 ± 0.01156 |
|  | Control | 14 |  |  | 0g | 21 | RR= 12023.6 ± 2285.56; Ratio of RR = 0.0157 ± 0.00331 |
| Gonzales et al., 2010 | Test | 10 | 10w | Close coil spring | 50g | 14 | Sig higher RR area DB root;1.2±0.0; DP root;1.1 ± 0.2; M root 0.3 ± 0.2 RR volume, DB root 22.0 ± 3.8; DP root 13.2 ± 2.3; Mesial root 0.3 ± 0.1 |
|  | Control | 10 |  |  | 0g | 14 | RR volume: DB root 35.6 ± 4.6; DP root 32.8 ± 5.1; M root 4.2 ± 1.4;  RR volume: DB root 0.9±0.1; DP root 0.8±0.1; Mesial root 0.3±0.2 |
| Gonzales et al., 2011 | Test | Gp1: 10 Gp2: 10 Gp3: 10 GP4: 10 | 4w | Close coil spring | 50g | Gp1:14 SF+14force Gp2:28 SF+28force Gp3:82 SF+14force GP4:o SF+14 force | Sig more RR volume and area% in test Gp RR volume DB root: GP1: 8.4 ± 1.2 X 10^6^ ; Gp2: 7.9 ± 1.7 X 10^6^ ; Gp3: 5.1 ± 0.6 X 106; GP4: 11.9 ± 1 X 106 DP root: Gp1: 7.2 ± 1.5 X 10^6^; Gp2: 5.8 ± 0.5 X 10^6^;Gp3: 2.7 ± 0.8 X 10^6^; Gp4: 8.3 ± 1.5 X 10^6^ M root: Gp1: 1.2 ± 0.1X 10^6^; Gp2: 0.8 ± 0.3 X 10^6^;Gp3: 0.5 ± 0.2X 10^6^; Gp4: 1.4 ± 0.10 X 10^6^ RR area %:DB root:GP1: 27.8±3.8;Gp2:27.8±3.8;Gp3:27.84.6; GP4:28.5±5 DP root: Gp1:23.2±6.3; Gp2:23.1±5.5; Gp3:22±4.4;Gp4:23.8±5.9 M root: Gp1:4.7±3.2;Gp2:4.1±1.7;Gp3:4.6±1.9; Gp4:5.7±3.2 |
|  | Control | Gp5: 10 |  |  | 0 | Gp 5: 0 SF + 0 force |  |
| Li et al., 2021 | Test | 20 | 4w, 11w | Close coil spring | 10g | 21 | Sig less RR volume 0.0869 ± 0.0244 |
|  | Control | 2 Gps 10 each |  |  | 0g | 21 | 0.1218 ± 0.0123 |
| Seifi et al., 2016 | Test | 10 | 4m | Close coil spring | 60g | 21 | Sig RR area percentage:0.277±0.001×10^-2^; Sig No of resorptive lacunae: 4.1667±0.892 |
|  | Control | 10 |  |  | 0g | 21 | RR area percentage 0.004 ± 0.001×10^-2^; No of resorptive lacunae: 0.2133 ± 0.417 |
| Seifi et al., 2017 | Test | 24 | 8w | Close coil spring | 60g | 21 | Sig RR area: 4.8830×10^-6^ ± 0.163×10^-6^ |
|  | Control | 24 |  |  | 0g | 21 | 1.7637×10^-6^ ± 0.503×10^-6^ |
| Sperl et al., 2020 | Test | 20 | 7w | Close coil spring | 25.5g(0.25N) | 35cetirizine,28force | Sig Both investigated cetirizine dosages had no impact on RR,  Sig increased RR at the OTM side of control animals |
|  | Test | 19 |  |  | 25.5g(0.25N) | 35cetirizine,28force |  |
|  | Control | 18 |  |  | 25.5g(0.25N) | 35tapwater,28force |  |
|  | Control | 58 |  |  | 0g | Cl side of the 3 Gp |  |
| Kirschneck et al., 2020 | Test | 10 | 7w | Close coil spring | 25.5g(0.25N) | 7 treatment, then 28 force+treatment | Sig increase in RR compared to un-treated CL side after 28days in all Gps: non-medicated group (p = 0.0242)  normal three days (p = 0.0110) normal seven days (p = 0.0500)  high dose etoricoxib (p = 0.0362) |
|  | Test | 10 |  |  | 25.5g(0.25N) |  |  |
|  | Test | 10 |  |  | 25.5g(0.25N) |  |  |
|  | Control | 10 |  |  | 25.5g(0.25N) |  |  |
|  | Control | 40 CL |  |  | 0g |  |  |
| Verna et al., 2006 | Test | 22 | NI | Close coil spring | 25g | 21 | Sig more RR in the acute group at the mesio-coronal level compared with the control and the chronic group. (P < 0.05): Sig more RR in the Force sides com-pared to the zero force CL side. Mean ± SD % of M and D sides: AC Gp: Coronal: 23.88 ± 14.32; Apical: 22.4 ± 17.61 |
|  | test | 23 |  |  | 25g |  | CC Gp: Coronal: 15.75 ± 11.78; Apical: 14.33 ± 10.33 |
|  | Control | 19 |  |  | 25g |  | NC Gp: Coronal: 15.16 ± 12.19; Apical: 12.83 ± 10.45 |
|  | Control | 19 |  |  | 0g |  | Control Gp: Coronal: 7.61 ± 7.69; Apical: 4.855 ± 5.9 |
| Lin et al., 2020 | Test | 15 | 8w | Close coil spring | 50g | 21 | Sig lower RR in non-treated Gp than both tested Gps; Sig lower RR area ratio in the baicalin Gp than normal saline Control  RR area%: 8.73±1.45 |
|  | Control | 15 |  |  | 50g |  | RR area%: 29.55 ± 4.61 |
|  | Control | 15 |  |  | 0g |  | No RR observed |
| Ullrich et al, 2021 | Test | 10 | 7w | Close coil spring | 25.5g | 14 | No Sig RR % between Gps(plot digitizer) ≈ 0.6 |
|  | Test | 10 |  |  | 25.5g | 14 | ≈0.2% |
|  | Control | 10 |  |  | 0g | 14 | ≈ 0.04 |
|  | Control | 10 |  |  | 0g | 14 | ≈0.04 |
| Yang et al., 2015 | Test | 12 | 6-8w | Close coil spring | 25g | 14,28 | Sig decreases in root volume and mesial root length in both 14 and 28d Gps than control Gp |
|  | Control | 12 |  |  | 0g | 14,28 |  |
| Zhuang et al., 2011 | Test | 11 | 11w | Close coil spring | 30g, 100g | 14 | Sig RR volume: 30g: 16 ± 2 ×10^-7^ 100g: 16.5 ± 1.5 x10^-7^ |
|  | Control | 11 |  |  | 0g | 14 | 13.5 ± 1.5×10^-7^ |
