# Supplementary material for: Under pressure—mechanisms and risk factors for orthodontically induced inflammatory root resorption: a systematic review
Source: Eur J Orthod. 2023 Jun 27;45(5):612–26. doi: 10.1093/ejo/cjad011 (PMC10505745; doi:10.1093/ejo/cjad011)
Supplement: cjad011_suppl_Supplementary_Table_S12 [file cjad011_suppl_supplementary_table_s12.docx]

| Supplementary Table 12: GRADE summary of findings for OIIRR outcome for Animal studies PI/ECO 2A and 2B | | | | | | | | | | |
| --- | --- | --- | --- | --- | --- | --- | --- | --- | --- | --- |
| **Authors** | **Study design** | **The initial quality of evidence** | **Risk of Bias** | **Inconsistency** | **indirectness** | **Imprecision** | **Publication bias** | **Other factors** | **Overall quality of evidence** | **Comments** |
| Aghili et al., 2013 | 4 Gps / SMD | High ⊗⊗⊗⊗ | Serious | Not Serious | Serious | Not Serious | Unlikely | No | Low ⊗⊗◯◯ | RoB and exposure indirectness |
| Aghili et al., 2015 | 2 Gps / SMD | High ⊗⊗⊗⊗ | Serious | Not Serious | Serious | Not Serious | Unlikely | No | Low ⊗⊗◯◯ | RoB and exposure indirectness |
| Akhoundi et al., 2016 | 4 Gps | High ⊗⊗⊗⊗ | Not Serious | Not Serious | Not Serious | Not Serious | Unlikely | No | High ⊗⊗⊗⊗ |  |
| Akhoundi et al., 2020 | 3 Gps | High ⊗⊗⊗⊗ | Not Serious | Not Serious | Not Serious | Not Serious | Unlikely | No | High ⊗⊗⊗⊗ |  |
| Asefi et al., 2018 | 4 Gps / SMD | High ⊗⊗⊗⊗ | Very Serious | Not Serious | Not Serious | No | Unlikely | No | Low ⊗⊗◯◯ | High RoB downgrade it by 2 |
| Bielaczyc et al., 1997 | 2 Gps | High ⊗⊗⊗⊗ | Very Serious | Not Serious | Very Serious | Serious | Unlikely | No | Very low ⊗◯◯◯ | insufficient reporting for results, indirectness with missing data and dichotomous outcome with high RoB |
| Brunet et al., 2016 | 4 Gps | High ⊗⊗⊗⊗ | Serious | Not Serious | Serious | Not Serious | Unlikely | No | Very low ⊗◯◯◯ | Short reporting for results and dichotomous as having or not having RR |
| Chung et al., 2008 | 2Gps / SMD | High ⊗⊗⊗⊗ | Serious | Not Serious | Not Serious | Not Serious | Unlikely | No | Moderate ⊗⊗⊗◯ | High RoB downgrade it by 1 |
| Crowther et al., 2017 | 2 Gps | High ⊗⊗⊗⊗ | Not Serious | Not Serious | Serious | Not Serious | Unlikely | No | Moderate ⊗⊗⊗◯ | Missing information in the outcome might downgrade it by 1 |
| Foo et al., 2007 | 4 Gps | High ⊗⊗⊗⊗ | Very Serious | Not Serious | Serious | Not Serious | Unlikely | No | Very low ⊗◯◯◯ | Reporting on crater volume and not the total RR, and very serious RoB |
| Gonzales et al., 2010 | 6 Gps / SMD | High ⊗⊗⊗⊗ | Very Serious | Not Serious | Not Serious | Not Serious | Unlikely | No | Low ⊗⊗◯◯ | High RoB downgrade it by 2 |
| Gonzales et al., 2011 | 5 Gps | High ⊗⊗⊗⊗ | Not Serious | Not Serious | Not Serious | Not Serious | Unlikely | No | High ⊗⊗⊗⊗ |  |
| Gul Amuk et al., 2020 | 3 Gps / SMD | High ⊗⊗⊗⊗ | Serious | Not Serious | Not Serious | Not Serious | Unlikely | No | Moderate ⊗⊗⊗◯ | RoB downgrade it by 1 |
| Jang et al., 2020 | 2 Gps | High ⊗⊗⊗⊗ | Very Serious | Not Serious | Serious | Not Serious | Unlikely | No | Very low ⊗◯◯◯ | Very serious RoB and indirectness |
| Kameyama et al., 2003 | 4 Gps | High ⊗⊗⊗⊗ | Very Serious | Not Serious | Not Serious | Not Serious | Unlikely | No | Low ⊗⊗◯◯ | High RoB downgrade it by 2 |
| King et al., 1997 | 2 Gps each subdivided into 6 Gps. | High ⊗⊗⊗⊗ | Very Serious | Not Serious | Very Serious | Not Serious | Unlikely | No | Very low ⊗◯◯◯ | Missing important information on outcome, reporting indirectly on the percentage of osteoclast and markers |
| Kirschneck et al., 2014 | 4 Gps | High ⊗⊗⊗⊗ | Not Serious | Not Serious | Not Serious | Serious | Unlikely | No | Low ⊗⊗◯◯ | Serious RoB and outcome reporting imprecision |
| Kirschneck et al., 2020 | 4 Gps / SMD | High ⊗⊗⊗⊗ | Serious | Not Serious | Not Serious | Not Serious | Unlikely | No | High ⊗⊗⊗⊗ |  |
| Konoo et al., 2001 | 3 Gps | High ⊗⊗⊗⊗ | Very Serious | Not Serious | Very Serious | Not Serious | Unlikely | No | Very low ⊗◯◯◯ | High RoB with outcome missing information and indirect reporting |
| Li et al., 2021 | 2Gps/three subGps | High ⊗⊗⊗⊗ | Very Serious | Not Serious | Not Serious | Not Serious | Unlikely | No | Low ⊗⊗◯◯ | RoB downgraded it by 2 |
| Lim et al., 2011 | 2 Gps | High ⊗⊗⊗⊗ | Very Serious | Not Serious | Very Serious | Not Serious | Unlikely | No | Moderate ⊗⊗⊗◯ | The outcome is missing important information and reports mostly on mineral contents |
| Lin et al., 2020 | 3 Gps | High ⊗⊗⊗⊗ | Serious | Not Serious | Not Serious | Not Serious | Unlikely | No | Moderate ⊗⊗⊗◯ | RoB downgraded it by 1 |
| MirHashemi et al., 2013 | 3 Gps | High ⊗⊗⊗⊗ | Serious | Not Serious | Not Serious | Not Serious | Unlikely | No | Moderate ⊗⊗⊗◯ | RoB downgraded it by 0 |
| Miyoshi et al., 2001 | 4 Gps | High ⊗⊗⊗⊗ | Very Serious | Not Serious | Not Serious | Not Serious | Unlikely | Dose-response gradient/ application-outcome relationship | Moderate ⊗⊗⊗◯ | High RoB downgraded it by 2, But dose-related effect upgraded it by 1 |
| Rafiei et al., 2015 | 2 Gps | High ⊗⊗⊗⊗ | Very Serious | Not Serious | Serious | Not Serious | Unlikely | No | Low ⊗⊗◯◯ | very serious and indirectness degrade it by 1 |
| Ru et al., 2016 | 3 Gps | High ⊗⊗⊗⊗ | Very Serious | Not Serious | Not Serious | Not Serious | Unlikely | No | Low ⊗⊗◯◯ | High RoB downgrade it by 1 |
| Seifi et al., 2015 | 4 Gps | High ⊗⊗⊗⊗ | Serious | Not Serious | Serious | Not Serious | Unlikely | No | Very low ⊗◯◯◯ | Measuring lacunae area, not total RR and high RoB downgraded it by 1 |
| Seifi et al., 2016 | 5 Gps | High ⊗⊗⊗⊗ | Serious | Not Serious | Not Serious | Not Serious | Unlikely | No | Low ⊗⊗◯◯ | High RoB downgrade it by 1 |
| Seifi et al., 2017 | SMD | High ⊗⊗⊗⊗ | Serious | Not Serious | Not Serious | Not Serious | Unlikely | No | Moderate ⊗⊗⊗◯ | Moderate RoB downgrade it by 1 |
| Shirazi et al., 2017 | 4 Gps | High ⊗⊗⊗⊗ | Very Serious | Not Serious | Serious | Not Serious | Unlikely | No | Low ⊗⊗◯◯ | Measuring lacunae depth, not total RR and high RoB downgraded it by 1 |
| Sperl et al., 2020 | 4 Gps/ SMD | High ⊗⊗⊗⊗ | Not Serious | Not Serious | Serious | Serious | Unlikely | No | Low ⊗⊗◯◯ | Outcome surrogate and dichotomous measures |
| Sringkarnboriboon et al., 2003 | 2 Gps | High ⊗⊗⊗⊗ | Very Serious | Not Serious | Not Serious | Not Serious | Unlikely | No | Low ⊗⊗◯◯ | Very serious RoB downgrade by 2 |
| Tyrovola et al., 2010 | SMD | High ⊗⊗⊗⊗ | Serious | Not Serious | Not Serious | Not Serious | Unlikely | No | Moderate ⊗⊗⊗◯ | RoB downgrade it by 1 |
| Ullrich et al., 2021 | 4 Gps / SMD | High ⊗⊗⊗⊗ | Not Serious | Not Serious | Serious | Not Serious | Unlikely | No | Moderate ⊗⊗⊗◯ | Outcome surrogate and need more information regarding RR |
| Verna et al., 2003 | 3 Gps / SMD | High ⊗⊗⊗⊗ | Very Serious | Not Serious | Not Serious | Not Serious | Unlikely | No | Low ⊗⊗◯◯ | High RoB downgrade it by 1 |
| Verna et al., 2006 | 3 Gps / SMD | High ⊗⊗⊗⊗ | Very Serious | Not Serious | Not Serious | Not Serious | Unlikely | No | Moderate ⊗⊗⊗◯ | RoB downgraded it by 0 |
| Yang et al., 2015 | SMD | High ⊗⊗⊗⊗ | Very Serious | Not Serious | Serious | Not Serious | Unlikely | No | Low ⊗⊗◯◯ | High RoB and reporting short |
| Yeoh et al., 2017 | 3 Gps / SMD | High ⊗⊗⊗⊗ | Not Serious | Not Serious | Not Serious | Not Serious | Unlikely | No | High ⊗⊗⊗⊗ |  |
| Yu et al., 2019 | 2 Gps | High ⊗⊗⊗⊗ | Not Serious | Not Serious | Serious | Not Serious | Unlikely | No | Moderate ⊗⊗⊗◯ | Outcome surrogate and indirect method of measurement |
| Zhuang et al., 2011 | 2 Gps / SMD | High ⊗⊗⊗⊗ | Very Serious | Not Serious | Not Serious | Not Serious | Unlikely | No | Low ⊗⊗◯◯ | High RoB downgrade it by 2 |

The outcome of interest is the externally induced inflammatory root resorption because meta-analyses were not conducted due to high heterogenicity between studies. Therefore, a single pooled effect estimate was unavailable, but a narrative synthesis of the evidence was provided).

Because the outcome for all interventions is the amount of OIIRR, some studies show more than 1 PI/ECOS. Therefore, for publication purposes, the individual GRADE tables for 2A and 2B PI/ECOs were collated into this single table for publication purposes.

The GRADE of evidence are; High quality: Further research is very unlikely to change our confidence in the estimate of effect; Moderate quality: Further research is likely to have an important impact on our confidence in the estimate of effect and may change the estimate; Low quality: Further research is very likely to have an important impact on our confidence in the estimate of effect and is likely to change the assessment; Very low quality: Any estimate of effect is very uncertain.
