# Supplementary material for: Under pressure—mechanisms and risk factors for orthodontically induced inflammatory root resorption: a systematic review
Source: Eur J Orthod. 2023 Jun 27;45(5):612–26. doi: 10.1093/ejo/cjad011 (PMC10505745; doi:10.1093/ejo/cjad011)
Supplement: cjad011_suppl_Supplementary_Table_S15 [file cjad011_suppl_supplementary_table_s15.docx]

| **Supplementary Table 15: PICO 3A, Studies' Summary** | | | | | | | | | | | |
| --- | --- | --- | --- | --- | --- | --- | --- | --- | --- | --- | --- |
| **Authors** | **Groups** | **Pt(N)** | **Age at the start of Treatment Mean ± SD (range) in years** | | **Intervention** | **Amount of force** | **Follow-up, mean ± SD (range)**  **m=months**  **w= weeks**  **d= days** | | **What to measure relevant to this study** | **Main outcomes ((Mean ± SD (range) Length in μm; area in μm^2^ , Volume in μm^3^ or N(%)** | **Main outcomes Finding (Sig=Significant Difference)** |
| Brin et al.,1991 | Test | 57 | 11.3 + 2.0 | | Tipping force | NI | Varied | | RR percentage | Sever RR 9 (7.8%); Absence106(91.4%) | Sig in: RR has more prevalence in test Gp than control Gp. |
|  | Control |  | NI | | Non | NI | Varied | |  | Absence 103 (99.0%) |  |
| Chan et al., 2004 | Test | 10 | NI | | Light tipping force Heavy tipping force | 25g 225g | 28d | | RR craters volumes | Total: 557.21 X 10^5^; Light: 291.69 X 10^5^; Heavy: 898.62 X 10^5^ | Sig in RR volume between experimental and control Gps |
|  | Control | 10 (CL) |  |  | no force | 0 gm | 28d | |  | Mean volume X 10^5^ μm^3^: 2.2 |  |
| Dermaut and Munck, 1986 | Test | 15 | 15 (11-37)y | | 3.6mm intrusion | 100g | 29(8-64)w | | Root length | 18% RR / original Root length RR in length: 2.5mm | Sig between mean root length between the Gps Low correlations of coefficients between the amounts of intrusion and RR; No relation between the duration of intrusion and RR |
|  | Control | 15 | 22(16-25)y | | no force | 0g | 28(24-32w | |  | 0% |  |
| Dudic, 2017 | Test | 30 | 16.7 (11.3-43) | | Tipping force | 101.97g | 8w | | RR volume | 0.00055 ± 0.00037) | Sig in RR between the two groups |
|  | Control | 30 | 16.7 (11.3-43) | | Non | 0g | 8w | |  | 0.00003 ± 0.00010 |  |
| Giannopoulou et al., 2008 | Test | 16(29 premolar) | 17.7 (11.3-43.0) | | Buccal tipping force | 101.97g (1 N) | 8w | | RR percentage, PI, GI, BOP, PPD | Severity of cervical resorption: Sever resorption: 93.02%; Moderate resorption: 3.88%; No resorption: 3.81% | Sig increase in resorption in test Gp than control Gp, No Sig between RR and patient hygiene level No Sig in Periodontal parameter(PI, GI, BOP, PPD) between the Gps, |
|  | Control | 16(CL 18 premol) |  |  | no force | 0g | 8w | |  | Severity of cervical resorption: Sever resorption: 5.8%; Moderate resorption: 31.22%; No resorption: 62.98% |  |
| Fontana et al., 2012 | Test | Gp1 160,  Gp 2 179 | 15.33 ± 2.64 (9.9-20) 14.50 ± 3.01 | | Variable force | NI | 6 m | | MeanEARR proportion (treated/untreated) | Gp1: 0.81 mm Gp2: 2.24 mm | Higher proportion of EARR in the treated patients (EARR ≤1.43 mm, 0.81 mm; EARR >1.43 mm, 2.24 mm) compared with the untreated subjects (EARR, 0.05 mm). A weak protective effect of allele C against EARR (CC 1 CT 3 TT). |
|  | Control | 38 | 16.64±1.93(11-19) | | Non | NI | 6 m | |  | Gp3: 0.05mm |  |
| Dalaie et al., 2021 | Test | 32 | (10-13) | | Alignment force | NI | 18 ± 3 m | | Change in root length | T2 Mean mm(SD): 13.64(0.51) T3 Mean mm(SD) : 14.42(0.63) | Sig increase in root length in the test group as compared to the control group |
|  | Control |  |  |  | Non | NI | 18 ± 3 m | |  | T2 Mean mm(SD): 13.17(0.54) T3 Mean mm(SD) : 14.36 (0.67) |  |
| Huang et al., 2021 | Test | 11 | 23.5 (18.3-37.7) | | Tipping force | 100g | 8w | | GCF, PI, GI | RR, lacunae volume: 0.72 ± 0.51 | Sig higher number and greater volume of resorption lacunae of test Gp than control Gp. Sig increased CEMP-1, CTX-I and decreased DPP secretion after 8w |
|  | Control |  |  |  | Non | 0g | 8w | |  | RR, lacunae volume: 0.33 ± 0.56 |  |
| Yilmaz et al, 2021 | Test1 | 20 | 16.77(15.08-18.58) | Orthodontic speed App (B force)+ Oral-B HummingBird device (vibration) | | 150g+50Hz vibration(10min/day) | | 12w | RR crater volume | RR volume: 0.476 ± 0.236 mm3 | Sig increase in RR volume in:  -Vibration and control no-force Gp -Force non-vibration Gp and control non-vibration Gp No Sig between RR volume in: -Vibration with force and force only gps. -Vibration with force and vibration only Gps. |
|  | Test2 |  |  | Orthodontic speed App (B force) | | 150g | | 12w |  | RR volume: 0.462 ± 0.337 mm4 |  |
|  | Test3 |  |  | Oral-B HummingBird device (vibration) | | 10m/day 50Hzvibration | | 12w |  | RR volume: 0.017 ± 0.337 mm4 |  |
|  | Control |  |  | No orthodontic movement | | 0g | | 12w |  | RR volume: 0.031 ± 0.085 mm5 |  |
