# Supplementary material for: Under pressure—mechanisms and risk factors for orthodontically induced inflammatory root resorption: a systematic review
Source: Eur J Orthod. 2023 Jun 27;45(5):612–26. doi: 10.1093/ejo/cjad011 (PMC10505745; doi:10.1093/ejo/cjad011)
Supplement: cjad011_suppl_Supplementary_Table_S17 [file cjad011_suppl_supplementary_table_s17.docx]

| Supplementary Table 17: GRADE summary of findings for OIIRR outcome for Human studies PI/ECO 3A and 3B | | | | | | | | | | |
| --- | --- | --- | --- | --- | --- | --- | --- | --- | --- | --- |
| **Authors** | **Study design** | **The initial quality of evidence** | **Risk of Bias** | **Inconsistency** | **indirectness** | **Imprecision** | **Publication bias** | **Other factors** | **Overall quality of evidence** | **Comments** |
| A. Iglesias-Linares et al., 2012 (Int Endod J) B. Iglesias-Linares et al., 2012 (J Endod) C. Iglesias-Linares et al., 2013 | A, Retrospective cohort B. Retrospective case-control C. Retrospective case-control | Low ⊗⊗◯◯ | Not Serious | Not Serious | Very Serious | Very Serious | Unlikely | No | Very low ⊗◯◯◯ | Participant differences and intervention are not uniform for all participants (different orthodontics mechanics), and outcomes are whether or not participants have more or less than 2mm of RR |
| Llamas-Carreras et al., 2010 | Retrospective cohort / SMD | Low ⊗⊗◯◯ | Not Serious | Not Serious | Not Serious | Not Serious | Unlikely | No | Low ⊗⊗◯◯ |  |
| Llamas-Carreras et al., 2012 | RCT/ SMD | High ⊗⊗⊗⊗ | Serious | Not Serious | Not Serious | Not Serious | Unlikely | No | Moderate ⊗⊗⊗◯ | The Risk of Bias downgrades it by 1 level |
| A. Pereira et al., 2016 B. Pereira et al., 2014 | Retrospective cohort | Low ⊗⊗◯◯ | Serious | Not Serious | Very Serious | Not Serious | Unlikely | No | Very low ⊗◯◯◯ | Reporting on different events of the same population for the same outcome |
| Al-Qawasmi et al., 2003a | Retrospective case-control | Low ⊗⊗◯◯ | Serious | Not Serious | Serious | Serious | Unlikely | No | Very low ⊗◯◯◯ | outcome reporting is missing important data about RR and surrogate and contain little information |
| Al-Qawasmi et al., 2003b | Retrospective case-control | Low ⊗⊗◯◯ | Not Serious | Serious | Very Serious | Serious | Unlikely | No | Very low ⊗◯◯◯ | The control group duration of treatment was not reported, outcome reporting is missing important data about RR, surrogate and contains little information |
| Behnaz et al., 2020 | Retrospective case-control | Low ⊗⊗◯◯ | Not Serious | Serious | Very Serious | Not Serious | Unlikely | No | Very low ⊗◯◯◯ | Different clinical cases with outcomes are dichotomous, either more or less 2mm of RR |
| Brin et al.,1991 | Retrospective cohort | Low ⊗⊗◯◯ | Not Serious | Serious1 | Very Serious | Not Serious | Unlikely | No | Very low ⊗◯◯◯ | clinical heterogeneity for the trauma type to the teeth results in inconsistency and indirectness. Also, reporting was short, and excluding was different among groups which affected the outcome |
| Brusveen et al., 2012 | Retrospective Cohort | Low ⊗⊗◯◯ | Not Serious | Not Serious | Not Serious | Not Serious | Unlikely | Plausible Confounders likely spurious the effect | Moderate ⊗⊗⊗◯ | The study found no association between impacted canine and incisors RR despite the confounders, which can upgrade the survey by 1 level |
| Chan et al., 2004 | RCT / SMD | High ⊗⊗⊗⊗ | Serious | Not Serious | Not Serious | Serious^1^ | Unlikely | Dose-response gradient/ application-outcome relationship | High ⊗⊗⊗⊗ | the imprecision would reduce quality by one level, but an increase in RR volume with an increase in the force would level it up by 1 |
| Ciurla et al., 2021 | Retrospective case-control | Low ⊗⊗◯◯ | Not Serious | Very Serious | Very Serious | Not Serious | Unlikely | No | Very low ⊗◯◯◯ | clinical heterogeneity and wide variety for treated patients and treatment mechanics and reporting on multiple factors on the same samples. |
| Dalaie et al., 2021 | RCT / SMD | High ⊗⊗⊗⊗ | Serious | Not Serious | Not Serious | Not Serious | Unlikely | No | High ⊗⊗⊗⊗ |  |
| de Castilhos et al., 2019 | Retrospective case-control study | Low ⊗⊗◯◯ | Not Serious | Not Serious | Not Serious | Not Serious | Unlikely | No | Low ⊗⊗◯◯ |  |
| Dermaut and Munck, 1986 | NRCI (CCT) | Low ⊗⊗◯◯ | Serious | Serious1 | Very Serious | Not Serious | Unlikely | No | very low⊗◯◯◯ | One very wide intrusion period between groups and within the groups different measuring periods and diff in population |
| Dudic, 2017 | RCT / SMD | High ⊗⊗⊗⊗ | Serious | Not Serious | Not Serious | Not Serious | Unlikely | No | High ⊗⊗⊗⊗ |  |
| Esteves et al., 2007 | Retrospective cohort/SMD | Low ⊗⊗◯◯ | Serious | Not Serious | Not Serious | Not Serious | Unlikely | No | Low ⊗⊗◯◯ |  |
| Fontana et al., 2012 | Retrospective case-control study | Low ⊗⊗◯◯ | Not Serious | Not Serious | Very Serious | Not Serious | Unlikely | No | Very low ⊗◯◯◯ | Outcome surrogate measures and indirectness as well dichotomous outcome above or below 1.43 mm of RR, lowering it |
| Fontana et al., 2012 | Retrospective case-control study | Low ⊗⊗◯◯ | Not Serious | Serious1 | Very Serious | Not Serious | Unlikely | No | very low⊗◯◯◯ | clinical heterogeneity for the trauma type to the teeth results in inconsistency and indirectness. Also, reporting was short, and excluding was different among groups, affecting the outcome |
| Giannopoulou et al., 2008 | RCT / SMD | High ⊗⊗⊗⊗ | Not Serious | Not Serious | Serious1 | Not Serious | Unlikely | No | Moderate ⊗⊗⊗◯ | Indirectness in outcome level it down by 1 |
| Gülden et al., 2009 | Retrospective case-control study | Low ⊗⊗◯◯ | Serious | Not Serious | Very Serious | Not Serious | Unlikely | No | Very low ⊗◯◯◯ | Different gps sizes and times were not reported |
| Guo et al., 2015 | Prospective case-control | Low ⊗⊗◯◯ | Serious | Not Serious | Serious | Not Serious | Unlikely | No | Very low ⊗◯◯◯ | Reporting multiple interrelated exposures of risk factors |
| Harris and Baker, 1990 | Retrospective cohort | Low ⊗⊗◯◯ | Not Serious | Not Serious | Serious | Not Serious | Unlikely | No | Low ⊗⊗◯◯ |  |
| Hendrix et al., 1994 | Retrospective Cohort | Low ⊗⊗◯◯ | Very Serious | Not Serious | Not Serious | Not Serious | Unlikely | No | Low ⊗⊗◯◯ |  |
| Huang et al., 2021 | RCT / SMD | High ⊗⊗⊗⊗ | Serious | Not Serious | Serious1 | Not Serious | Unlikely | No | Moderate ⊗⊗⊗◯ | Indirectness of evidence and outcomes lower it down by one level |
| Iber-Díaz et al, 2020 | Retrospective cohort | Low ⊗⊗◯◯ | Not Serious | Not Serious | Very Serious | Serious | Unlikely | No | Very low ⊗◯◯◯ | Population difference, studying multiple exposure and risk factors and reporting dichotomous either or not having RR more than 5mm |
| Iglesias-Linares et al, 2012 | Retrospective Case-control | Low ⊗⊗◯◯ | Not Serious | Not Serious | Very Serious | Very Serious | Unlikely | No | Very low ⊗◯◯◯ | Participant differences and intervention are not uniform for all participants (different orthodontics mechanics), and outcomes are whether or not participants have more or less than 2mm of RR |
| Iglesias-Linares et al., 2014 | Retrospective Case-control | Low ⊗⊗◯◯ | Not Serious | Not Serious | Very Serious | Very Serious | Unlikely | No | Very low ⊗◯◯◯ | Participant differences and intervention are not uniform for all participants (different orthodontics mechanics), and outcomes are whether or not participants have more or less than 2mm of RR |
| Karadeniz et al., 2011 | RCT | High ⊗⊗⊗⊗ | Not Serious | Not Serious | Not Serious | Not Serious | Unlikely | Dose-response gradient/application-outcome relationship | High ⊗⊗⊗⊗ | Dose-related difference |
| Kolcuoglu and Zeynep Oz, 2019 | NRCI(CCT)/ SMD | Low ⊗⊗◯◯ | Serious | Very Serious | Not Serious | Not Serious | Unlikely | No | Low ⊗⊗◯◯ |  |
| Kook et al., 2003 | Retrospective cohort | Low ⊗⊗◯◯ | Not Serious | Not Serious | Not Serious | Not Serious | Unlikely | No | Low ⊗⊗◯◯ |  |
| Lee and Lee, 2015 | Retrospective Cohort / SMD | Low ⊗⊗◯◯ | Serious | Not Serious | Not Serious | Not Serious | Unlikely | No | Very low ⊗◯◯◯ | The risk of Bias downgraded the evidence by 1 level |
| Li et al., 2020 | Retrospective cohort | Low ⊗⊗◯◯ | Not Serious |  | Not Serious | Not Serious | Unlikely | The large magnitude of the effect | ⊗⊗⊗◯ Moderate | The large magnitude of the effect upgraded it by I level |
| Linhartova et al., 2013 | Retrospective case-control study | Low ⊗⊗◯◯ | Not Serious | Not Serious | Serious | Serious | Unlikely | No | Very low ⊗◯◯◯ | Different population sizes between groups, reporting the dichotomous outcome of either more than 2mm or less than 2mm of RR |
| Linhartova et al., 2017 | Retrospective case-control study | Low ⊗⊗◯◯ | Not Serious | Not Serious | Serious | Serious | Unlikely | No | Very low ⊗◯◯◯ | Different population sizes between groups, reporting dichotomous outcomes of either more than 2mm or less than 2mm of RR |
| Malmgren et al., 1982 | Retrospective cohort /SMD | Low ⊗⊗◯◯ | Very Serious | Very Serious | Very Serious | Not Serious | Unlikely | Plausible Confounders spurious the effect | Very low ⊗◯◯◯ | Clinical heterogeneity, different populations and treatment time, and reporting on multiple factors downgraded the study even though the result does not support the hypothesis that traumatized teeth have more RR. |
| Mann et al., 2022 | CCT | Low ⊗⊗◯◯ | Serious | Not Serious | Not Serious | Not Serious | Unlikely | No | Moderate ⊗⊗⊗◯ | High RoB downgrade it by 1 level |
| Marques et al., 2010 | Retrospective case-control | Low ⊗⊗◯◯ | Serious | Serious | Very Serious | Serious | Unlikely | No | Very low ⊗◯◯◯ | multiple exposures being studies, outcomes are short with missing important information |
| McNab et al., 1999 | Retrospective Cohort | Low ⊗⊗◯◯ | Not Serious | Not Serious | Serious | Not Serious | Unlikely | No | Very low ⊗◯◯◯ | Differences in population size between groups |
| Melo et al., 2018 | Retrospective case-control study | Low ⊗⊗◯◯ | Not Serious | Serious | Very Serious | Not Serious | Unlikely | No | Very low ⊗◯◯◯ | Different treatment times, population size and reporting on other factors not related to asthma |
| Mirabella and Årtun, 1995 | Prospective case-control | Low ⊗⊗◯◯ | Very Serious | Serious | Serious | Serious | Unlikely | No | Very low ⊗◯◯◯ | With high RoB multiple exposures being studied, outcomes are short with missing important information |
| Motokawa et al., 2013 | Retrospective cohort | Low ⊗⊗◯◯ | Not Serious | Very Serious | Not Serious | Not Serious | Unlikely | No | Very low ⊗◯◯◯ | clinical heterogeneity for treated patients and treatment mechanics can lead to different outcomes. |
| Nanekrungsan et al., 2012 | Retrospective Case-control | Low ⊗⊗◯◯ | Serious | Serious | Very Serious | Serious | Unlikely | No | Very low ⊗◯◯◯ | Clinical heterogenicity and methodology variables for a patient treated a long time before the study conducted, outcomes are dichotomous whether or not having RR, multiple variable factors affecting the same population |
| Nigul and Jagomagi, 2006 | Retrospective cohort | Low ⊗⊗◯◯ | Serious | Very Serious | Very Serious | Serious | Unlikely | No | Very low ⊗◯◯◯ | Clinical heterogenicity and methodology variables for a different patient, multiple variable factors affecting the same population |
| Nishioka et al., 2006 | Retrospective case-control | Low ⊗⊗◯◯ | Not Serious | Serious | Very Serious | Serious | Unlikely | No | Very low ⊗◯◯◯ | multiple exposures being studies, outcomes are short with missing important information |
| Owman-Moll and Kurol, 2000 | Retrospective Case-control | Low ⊗⊗◯◯ | Very Serious | Not Serious | Very Serious | Serious | Unlikely | No | Very low ⊗◯◯◯ | High RoB with multiple predisposing factors being studied, outcomes are short with missing important information |
| Picanço et al., 2013 | Retrospective cohort | Low ⊗⊗◯◯ | Not Serious | Very Serious | Very Serious | Not Serious | Unlikely | No | Very low ⊗◯◯◯ | Participant heterogenicity, different treatment mechanics, and multiple exposure factors that intervene with each other affect the quality of the outcome |
| Sameshima and Sinclair, 2004 | Retrospective, case-control | Low ⊗⊗◯◯ | Very Serious | Serious | Serious | Not Serious | Unlikely | No | Very low ⊗◯◯◯ | population variation with multiple variable risk factors downgrade it by one |
| Sharab et al., 2015 | Retrospective case-control study | Low ⊗⊗◯◯ | Not Serious | Not Serious | Very Serious | Serious | Unlikely | No | Very low ⊗◯◯◯ | Incostenciency in methodology, different populations and treatment time between control and test groups, reporting on multiple possible interconnected risk factors downgraded the evidence to very low |
| Spurrier et al., 1990 | Retrospective cohort/ SMD | Low ⊗⊗◯◯ | Not Serious | Not Serious | Not Serious | Not Serious | Unlikely | No | Low ⊗⊗◯◯ |  |
| Taner et al., 1999 | Retrospective Cohort | Low ⊗⊗◯◯ | Not Serious | Not Serious | Serious | Not Serious | Unlikely | No | Very low ⊗◯◯◯ | The intervention and mechanics for orthodontic appliances might differ for patients with Cl II than Cl I |
| Villa et al., 2005 | NRCI(CCT) / SMD | Low ⊗⊗◯◯ | Very Serious | Not Serious | Very Serious | Not Serious | Unlikely | No | Very low ⊗◯◯◯ | Very Serious RoB and outcomes need to be more specific for measuring the RR amount and not the number of resorption lacunae |
| Yilmaz et al., 2021 | RCT / SMD | High ⊗⊗⊗⊗ | Not Serious | Not Serious | Not Serious | Serious^1^ | Unlikely | No | Moderate ⊗⊗⊗◯ | Imprecision results in lowering the quality by 1 level |

The outcome of interest: is the externally induced inflammatory root resorption because meta-analyses were not conducted due to high heterogenicity between studies. Therefore, single pooled effect estimate was unavailable, but a narrative synthesis of the evidence was provided).

Because the outcome for all interventions is the amount of OIIRR, some studies show more than 1 PI/ECOS. Therefore, for publication purposes, the individual GRADE tables for 3A and 3B PI/ECOs were collated into this single table for publication purposes.

The GRADE of evidence are; High quality: Further research is very unlikely to change our confidence in the estimate of effect; Moderate quality: Further research is likely to have an important impact on our confidence in the estimate of effect and may change the estimate; Low quality: Further research is very likely to have an important impact on our confidence in the estimate of effect and is likely to change the estimate; Very low quality: Any estimate of effect is very uncertain.
